# T-World Virtual Human Cardiomyocyte. I. Development, Validation, and Cell Arrhythmogenesis

**DOI:** 10.1161/CIRCRESAHA.125.328073

**Published:** 2026-04-07

**Authors:** Jakub Tomek, Maxx Holmes, Thomas Bury, Marketa Tomkova, Heeseung Jo, Norbert Nagy, Ambre Bertrand, Alfonso Bueno-Orovio, Michael A. Colman, Blanca Rodriguez, Donald M. Bers, Jordi Heijman

**Affiliations:** Department of Anatomy, Physiology and Genetics (J.T., H.J.), University of Oxford, United Kingdom.; Department of Computer Science (M.H., A.B., A.B.-O., B.R.), University of Oxford, United Kingdom.; Ludwig Cancer Research (M.T.), University of Oxford, United Kingdom.; Department of Pharmacology, University of California, Davis (J.T., D.M.B.).; Department of Physiology, McGill University, Montreal, CA (T.B.).; Department of Pharmacology and Pharmacotherapy, University of Szeged, Hungary (N.N.).; HUN-REN-SZTE Research Group for Cardiovascular Pharmacology, Szeged, Hungary (N.N.).; School of Biomedical Sciences, University of Leeds, United Kingdom (M.A.C.).; Gottfried Schatz Research Center, Division of Medical Physics and Biophysics, Medical University of Graz, Austria (J.H.).; Department of Cardiology, Cardiovascular Research Institute Maastricht (CARIM), Faculty of Health, Medicine and Life Sciences, Maastricht University, the Netherlands (J.H.).

**Keywords:** action potentials, arrhythmias, cardiac, cardiovascular diseases, morbidity, myocytes, cardiac

## Abstract

**BACKGROUND:**

Cardiovascular disease is the leading global cause of morbidity and mortality. New technologies are needed to improve mechanistic understanding and inform therapeutic strategies. Human-centric cardiac simulations show great promise; however, existing cellular models can reproduce only a few arrhythmia-driving behaviors and show important discrepancies with experimental data. We aimed to develop a new model overcoming this lack of generality, which markedly limits the predictivity and translational utility of virtual cardiomyocytes.

**METHODS:**

We developed T-World, a novel virtual human cardiomyocyte, using data-driven differential equations to describe sex-specific excitation-contraction coupling, mechanical contraction, β-adrenergic signaling, and its effects on cellular targets. The model contains several key innovations, including a new approach to coupling L-type calcium channels and ryanodine receptors, with updated calcium-dependent-inactivation of the former and novel calcium-induced refractoriness and complete reparameterization of the latter. We also redeveloped the sodium-potassium pump and made major improvements to the sodium-calcium exchanger formulation.

**RESULTS:**

T-World shows broad agreement with experimental data on rate-dependent action potential (AP), calcium handling, and contraction properties. Extensively validated on independent data, T-World demonstrates strong predictive performance, for example, in drug-induced AP changes. The model reproduces the effects of sympathetic stimulation, including AP duration shortening and increased calcium-transient amplitude and contractility. Importantly, it recapitulates for the first time all key cellular mechanisms driving life-threatening arrhythmias (early and delayed afterdepolarizations, alternans, and steep S1-S2 restitution), including experimentally observed responses to interventions such as sympathetic activation, SERCA (sarco/endoplasmic reticulum Ca^2+^ ATPase) inhibition, and AP prolongation. Combined with the model’s ability to simulate physiological sex-specific differences in electrophysiology, this revealed increased proclivity of female cardiomyocytes to early afterdepolarizations and steep restitution of AP duration.

**CONCLUSIONS:**

T-World is a highly general and predictive open-source computer model of a human ventricular cardiomyocyte, suitable for multiscale research studies investigating determinants of arrhythmogenesis.

**Novelty and Significance**

**What Is Known?**

- Computer models of human cardiomyocytes (virtual cardiomyocytes) are an increasingly prioritized technology in academia and industry.
- Existing virtual cardiomyocytes can only reproduce a limited number of cellular arrhythmia mechanisms and show major discrepancies with experimental knowledge.
- This markedly limits their utility for studying interactions of different mechanisms and disease pathways.

**What New Information Does This Article Contribute?**

- We developed T-World, a human ventricular myocyte showing comprehensive agreement with human physiology, which can simulate all key arrhythmia-driving behaviors within a single framework.
- T-World integrates excitation-contraction coupling, sympathetic nervous signaling, and sex differences, indicating a novel sex-specific arrhythmia risk in females.
- The model was extensively validated on unseen data, showing strong predictive performance against independent experimental data sets, including drug responses and modulation of SERCA (sarco/endoplasmic reticulum Ca^2+^ ATPase) activity.

Computational models of human ventricular cardiomyocytes are increasingly used to study arrhythmia mechanisms and to support drug development, yet existing models typically capture only isolated aspects of cardiac pathophysiology. This study introduces T-World, a highly general computer model of the human cardiomyocyte that integrates electrophysiology, calcium handling, mechanical contraction, β-adrenergic signaling, and sex differences within a single coherent framework. For the first time, one model reproduces all major cellular arrhythmogenic mechanisms under experimentally relevant conditions while maintaining quantitative agreement with independent human data. By combining mechanistic rigor with extensive validation, T-World overcomes longstanding trade-offs between physiological accuracy and arrhythmia capability that limited prior models. The model also provides new insight into sex-specific arrhythmic vulnerability, suggesting steeper restitution and greater early afterdepolarization susceptibility in female myocytes. These advances establish T-World as a broadly applicable platform for mechanistic discovery, drug safety testing, disease modeling, and future multiscale simulations, thereby enhancing the translational potential of human-centric cardiac simulations and potentially reducing reliance on animal experimentation.

**Meet the First Author, see p e000754 | Editorial, see Article by Kucera**

Computational modeling and simulations of cardiac cellular and organ physiology have become an integral part of contemporary cardiovascular research, providing insights into basic physiological mechanisms and mechanisms of arrhythmia.^1^ Moreover, these models of human cardiac (patho)physiology are increasingly used in therapy guidance,^2^ and drug safety assessment.^3,4^

Computational modeling and simulations also hold tremendous potential for reducing, replacing, and refining the use of animals in research (3R principles). Although some animal-based studies in cardiac research are indispensable, simulations can minimize animal use by guiding experimental design, predicting outcomes, and aiding interpretation. Human-specific virtual cells can also predict functional implications of animal data in the context of human physiology, addressing critical species differences that may, for example, make a drug safe in mice but dangerous in humans.^5^ Correspondingly, the European Medicines Agency, Food and Drug Administration, and UK government have recognized computational modeling as a key trend in advancing 3R principles.^6–8^

Multiple successful models of human ventricular cardiomyocytes have been developed to investigate specific mechanisms of cardiac (patho)physiology and arrhythmogenesis. The Rudy-family models (eg, ORd and ToR-ORd) excel at predicting drug responses and generating early afterdepolarizations (EADs) under realistic conditions, making them valuable for drug safety and efficacy assessment.^3,4^ The Bers/Grandi family models, for example, the recent model by Morotti et al (Morotti2021) used to study interspecies differences, are known for realistic calcium handling.^9–11^ The Ten Tusscher-Panfilov 2006 model (TP06) is widely used to study arrhythmia related to restitution properties.^12^ Despite their strengths, each model family lacks generality, capturing only a small subset of arrhythmic behaviors and manifesting important discrepancies with experimental data on fundamental physiology. This limits their utility for mechanistic studies, analyzing multifactorial drug effects, modeling complex diseases such as type 2 diabetes and heart failure, or integrative arrhythmia studies. Cardiomyocytes and their models are highly complex and include numerous components connected through nonlinear feedback loops. As a result, flaws in one model component can cascade, leading to incorrect predictions in other components and behaviors. This limits the predictive power and usefulness of previous models beyond their original focus. At the same time, the most innovative and relevant applications often arise precisely in these out-of-domain contexts. The lack of generality is in part also why different cellular models have typically been used to study aspects of arrhythmogenesis at cellular versus organ levels.^1^

The absence of a comprehensive and physiologically accurate virtual cardiomyocyte impedes progress toward translational applications and expanding the context of use of cardiac simulations. To address this gap and unlock the full potential of cardiac simulations in research, industry, and clinics, we aimed to develop a unified, highly general virtual cardiomyocyte. The generality should include: (1) Accurate recapitulation of human cellular cardiac physiology and its modulation by drugs or physiological changes. (2) The capability to manifest all key arrhythmogenic behaviors in conditions used to provoke them experimentally; this comprises EADs and delayed afterdepolarizations (DADs),^13,14^ alternans,^15^ and steep restitution of action potentials (APs).^16^ (3) Suitability for multiscale modeling, enabling organ-level simulations.

In the first of 2 back-to-back articles, we present T-World, a novel virtual human cardiomyocyte that reproduces for the first time all key arrhythmogenic mechanisms while maintaining physiological accuracy across diverse conditions. Comparison to independent data not used in model creation demonstrates robust predictive accuracy at a broad range of tasks. T-World integrates electrophysiology, calcium handling, cardiomyocyte contraction, sympathetic stimulation, and sex differences, enabling comprehensive studies of their interactions. T-World is freely available via Matlab and CellML, with a free-to-use online graphical interface for noncoders (also runnable in Python). The second article on T-World demonstrates its utility for organ-scale simulations, pharmacological studies, and novel insights into disease mechanisms.^17^

## Methods

### Data and Source Code Availability

T-World is distributed as open-source code and is available at https://github.com/jtmff/TWorld, including sample scripts demonstrating its functionality. An online graphical user interface enabling running T-World simulations is available at https://t-world-simulator-multipage-production.up.railway.app/. Background of the T-World development, including the description of various dead ends that we encountered during development, will be provided at the blog underlid.blogspot.com.

### Overview of Methodology

T-World is a virtual cell model using sets of ordinary differential equations to describe, based on experimental data, the dynamics of ionic currents, fluxes, and subcellular signaling. The overall cell architecture and calcium handling were mainly inspired by the Bers/Grandi family of models,^9–11^ with most ionic current formulations inspired by the ToR-ORd model.^4^

To enable all key arrhythmic behaviors under relevant experimental conditions, and to avoid limitations of preceding frameworks with regard to basic physiological behaviors and response to (patho)physiological changes, we introduced developments across nearly every component of the cell, including ionic currents, calcium handling, and autonomic signaling. Among other changes, T-World includes (1) a new scheme of coupling L-type calcium channels and RyRs (ryanodine receptors), (2) addition of a calcium-dependent-inactivation gate for the L-type calcium channel and reformulation of the model around this change, including adaptations to the modeling of the driving force for calcium ions passing the membrane; (3) new calcium-induced refractoriness state and extensive reparameterization of the RyR model; (4) altered voltage dependence of the sodium-potassium pump, based on reassessment of data sources; (5) major improvement of a prior, well-established NCX (sodium-calcium exchanger) formulation, enabling appropriate simulation of the effects of sodium overload; and (6) development of a representation of the effects of sympathetic nervous signaling on the heart, for example, adding modulation of the contractile apparatus by this pathway. These core innovations enable realistic calcium dynamics and arrhythmogenic behaviors (see Results), solving limitations that persisted in the field for decades.

It should be noted that these advances are not superficial updates and that obtaining a model that combines the strengths of previous models without their limitations represents a major technical and conceptual difficulty. In complex, nonlinear systems like cardiac cells, direct combinations often introduce critical errors because models of cellular components are pushed beyond their intended use. T-World succeeds because of innovative integration strategies, rigorous validation, and the development of mechanistic modules, described in detail in the Supplemental Methods.

The model was developed through iterative interactions between human-driven biophysical understanding and automated multiobjective optimization. For example, model structures for new components were initially guided by experimental insights, followed by numerical optimization of the structure’s parameters and subsequent analysis of model performance, leading to further adjustments and optimization of the model structure based on our understanding of the strengths and weaknesses of the formulation. Multiobjective genetic algorithms were used to fit parameters of single model components, as well as for integrating components. In these algorithms, a range of protocols would be simulated with a candidate model, with the simulation outputs compared with reference values based on experimental data. Detailed considerations, including the dimension of the objective function, population size, and number of iterations, as well as the exact formulation of the final fitness functions used, are described in the Supplemental Methods.

The World in the model’s name reflects the fact that model designs and expertise from the whole world were essential in its creation, and it goes beyond outputs of a single group, while the T stands for the last name of the first author, who brought this expertise together and was the driving force behind the model’s development.

Please see Supplemental Methods for a detailed description of the following:

1. Model architecture.
2. Calibration and validation criteria for T-World development and evaluation.
3. Description of equations describing the ionic currents and fluxes (Figures S1 through S3).
4. Contractility representation (Figure S4).
5. CaMKII (calcium/calmodulin-dependent protein kinase-II) and βARS (β-adrenergic) signaling (Figure S5).
6. Sex differences.
7. Methodology for studies on arrhythmogenic behaviors.
8. Graphical user interface.
9. Notes on implementation.
10. Sources of implementation of other models.

### Statistics

The computational models used in this study are deterministic models that, for a given set of conditions, will always produce the same steady-state output. As such, no error bars can be shown for baseline model output, and statistical comparisons between the output of 2 model instances are not possible. Similarly, no statistical comparisons of model populations are provided, as the number of model instances that can be evaluated is arbitrarily large, resulting in arbitrarily small confidence intervals for the point estimates of the model output. Assessment of the relative performance of different models and variants is based on whether key qualitative behaviors and features are reproduced or not, and on visual assessment of the provided model outputs and experimental data.

## Results

### Model Overview and Key Outputs: AP, Calcium Transient, and Force

**Figure 1.**
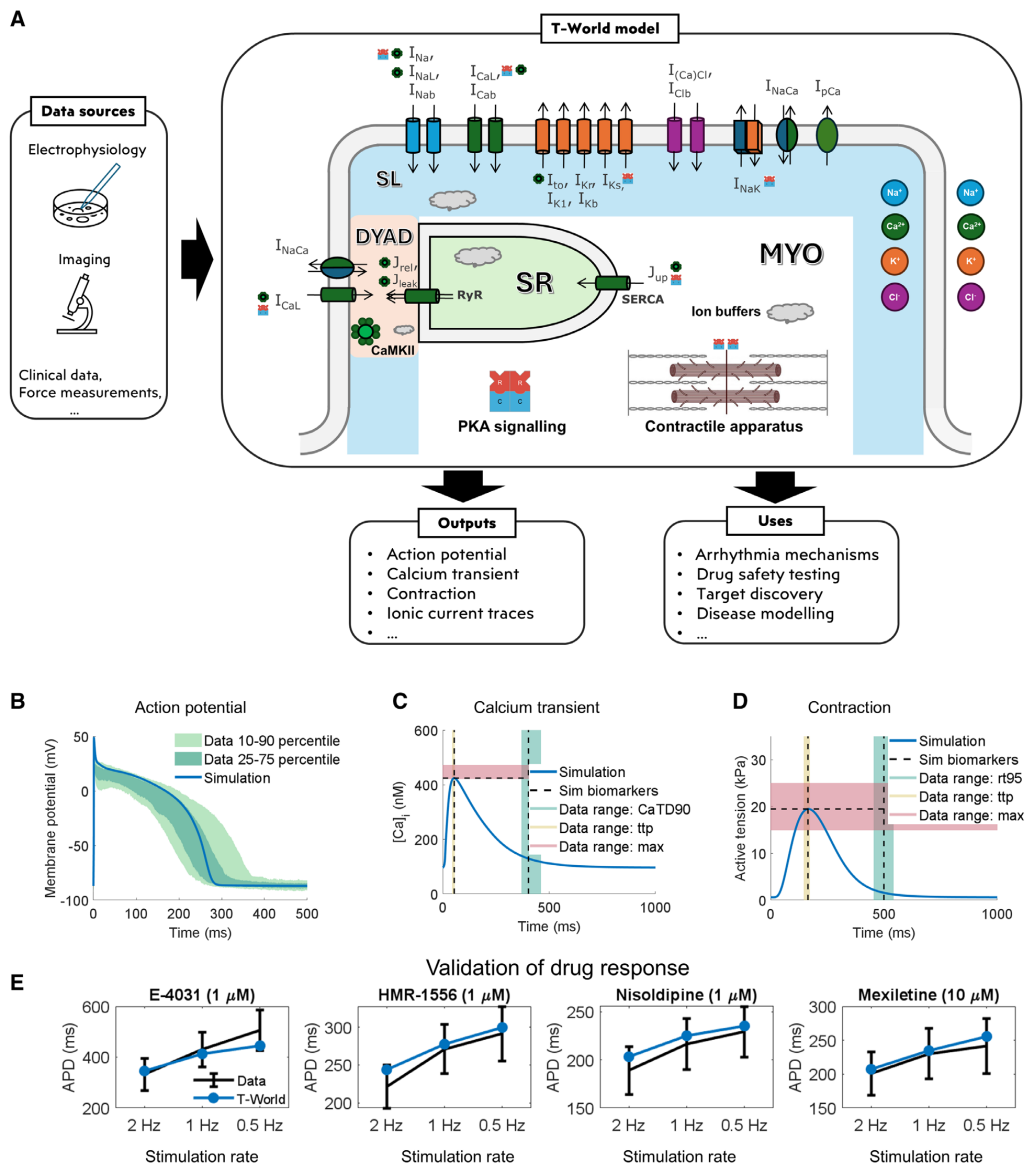
**Model structure and key outputs. A**, Conceptual diagram of the T-World model and its potential applications. Model diagram shows the membrane with ionic currents (color-coded by ionic species). The inside of the cell is separated into the following compartments: junctional dyad (DYAD, orange), subsarcolemmal (SL, blue), bulk myoplasm (MYO), and sarcoplasmic reticulum (SR, green). CaMKII (calcium/calmodulin-dependent protein kinase-II), and PKA (protein kinase-A) signaling pathways are included, with small icons adjacent to names of ionic currents, fluxes, or contractile apparatus indicating the site as a target of a given signaling pathway. Gray clouds indicate ionic buffers. **B**, Endocardial action potential (AP) of T-World vs experimental ranges.^18^ A slightly higher peak membrane potential in simulation vs data was chosen, given that our model is a single cell, whereas the experimental data are in small tissue samples, which show a reduced peak due to cell-to-cell coupling. **C**, Calcium transient (CaT) of T-World with highlighted biomarkers vs experimental ranges for: CaT duration at 90% recovery level (CaTD90, shown in green), time to peak (ttp, shown in yellow), and CaT amplitude (shown as amplitude+diastolic level to facilitate comparison, in red), based on SE of mean ranges by Coppini et al.^19^ **D**, Active tension developed by T-World with highlighted biomarkers vs experimental ranges for: time from peak to 95% recovery (rt95, shown in green), ttp (shown in yellow), and maximum active tension (max, shown in red), based on Margara et al.^20^ **E**, Validation of the simulated AP duration (APD) in the presence of 1 µmol/L E-4031 (70% I_Kr_ block), 1 µmol/L HMR-1556 (90% I_Ks_ block), 1 µmol/L nisoldipine (90% I_CaL_ block), and 10 µmol/L mexiletine (54% I_NaL_, 9% I_Kr_, 20% I_CaL_ block) at 0.5, 1.0, and 2.0 Hz pacing, using independent experimental data not used in model creation. Drug concentrations and their inhibitory effects on different currents are based on O’Hara et al.^18^ Please note the distinct *y* axes for the 4 drugs. [Ca]_i_ indicates cytosolic calcium concentration; I_(Ca)Cl_, calcium-activated chloride current; I_Cab_, background calcium current; I_CaL_, L-type calcium current; I_Clb_, background chloride current; I_K1_, basal inward-rectifier potassium current; I_Kb_, background potassium current; I_Kr_, rapid delayed-rectifier potassium current; I_Ks_, slow delayed-rectifier potassium current; I_Na_, fast sodium current; I_Nab_, background sodium current; I_NaCa_, sodium-calcium exchanger current; I_NaK,_ sodium-potassium pump current; I_NaL_, late sodium current; I_pCa_, sarcolemmal calcium pump current; I_to_, transient outward potassium current; J_leak_, SR calcium leak flux; J_rel_, SR calcium release flux; J_up_, SR calcium uptake flux; RyR, ryanodine receptor; and SERCA, sarco/endoplasmic reticulum Ca^2+^ ATPase.

Based on extensive experimental data, the T-World model incorporates a broad range of ionic currents and fluxes across distinct cellular compartments, as well as subcellular signaling pathways and contractility (see Figure 1A for a high-level overview). Distinct model components are described by sets of ordinary differential equations, constructed to recapitulate baseline experimental data on single ionic currents and other cellular elements. Coupling all those components together yields a virtual cardiomyocyte.

The 3 key outputs of a cardiomyocyte model are its AP, calcium transient (CaT), and the resulting active tension during contraction. T-World shows a strong agreement with human AP data^18^ with regards to AP duration (APD), resting membrane potential, and the overall shape of the AP during plateau and recovery (Figure 1B). It is in better agreement with human AP shape than most prior state-of-the-art models (Figure S6). The T-World CaT is also in excellent agreement with human data on time to peak, duration, and amplitude^19^ (Figure 1C). T-World incorporates the Land model of contraction^21^ as in the work of Margara et al,^20^ and its mechanics outputs are fully consistent with experimental data on time to peak force, amplitude, and time to 95% recovery of contraction in human myocardium^20^ (Figure 1D) while maintaining fundamental properties such as the cellular equivalent of the Frank-Starling law present in the Land model.

A given AP shape can be achieved through various combinations and balances of underlying currents.^22^ The balance of key ionic currents in T-World was validated by simulating its exposure to 4 channel-blocking drugs at 3 pacing rates (Figure 1E). T-World retains the strong predictive performance of ToR-ORd, showing excellent agreement with human data despite having a different cell architecture and calcium handling (Figure S7), predisposing T-World to applications in safety pharmacology. The Morotti2021 model is also mostly in agreement with the data at the more physiological heart rates (1–2 Hz), whereas the TP06 model shows substantial discrepancies with the experimental data (Figure S7).

### Excitation-Contraction Coupling

Calcium-induced calcium release in cardiomyocytes involves calcium entering the cell via L-type calcium channels (I_CaL_), which activate RyRs on the sarcoplasmic reticulum (SR) to release additional calcium. Different models represent this process in distinct ways, each with specific strengths and limitations.

Models like ORd and ToR-ORd couple RyR release flux directly to I_CaL_, yielding a realistic CaT shape, but this is mechanistically unrealistic and prevents spontaneous calcium release and DADs arising from RyR dysfunction or calcium overload. In contrast, Bers/Grandi–like models, including Morotti2021, base RyR opening on local calcium concentrations, which is mechanistically accurate, but causes delayed SR calcium release, as shown below. To address these limitations, T-World uses a hybrid approach. A small subset of RyR channels is I_CaL_-dependent (representing the most closely associated I_CaL_ and RyR clusters), providing early calcium influx that primes the larger, calcium-sensitive RyR population, that are based on a revised version of the Shannon/Grandi RyR. This yields well-timed calcium-induced calcium release that is both calcium-sensitive and capable of generating DADs.

**Figure 2.**
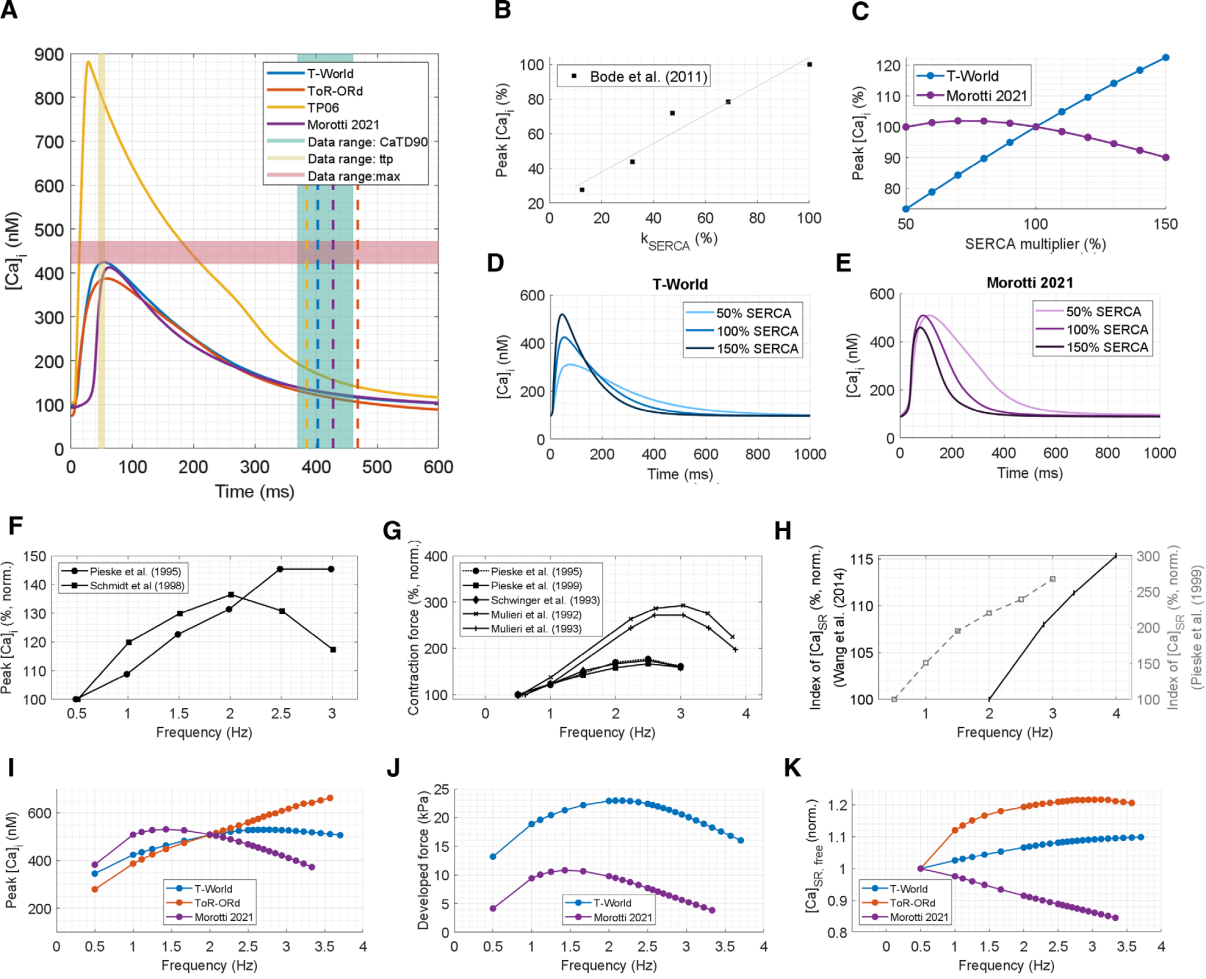
**Properties of excitation-contraction coupling. A**, Comparison of 4 models (T-World, ToR-ORd, Ten Tusscher-Panfilov 2006 [TP06], and Morotti 2021) and experimental ranges for calcium transient (CaT) properties; dashed lines give the CaT duration at 90% recovery for each model. **B** and **C**, Experimental data^23^ (**B**) and model results (**C**) on the relationship between SERCA (sarco/endoplasmic reticulum Ca^2+^ ATPase) activity and peak calcium concentration. **D** and **E**, Sample traces of CaTs at 3 different SERCA levels. **F** through **H**, Experimental data for rate-dependence of peak calcium concentration,^24,25^ developed force,^24,26–29^ and sarcoplasmic reticulum (SR) calcium concentration.^26,30^ In **H**, the Pieske et al^26^ study in human samples used rapid cooling contractures, which is a less direct estimate of calcium concentration in the SR ([Ca]_SR_) than the direct measurement of normalized (norm.) fluorescence by Wang et al^30^ in rabbit. **I** through **K**, Corresponding simulations of rate-dependence in computational models. In **J**, only the models that include force generation are shown. The rate dependence of ToR-ORd depends on prepacing duration (Figure S10), whereas TP06 rate-dependence is very steep and is thus shown separately in Figure S11. [Ca]_i_ indicates cytosolic calcium concentration.

Despite different calcium-handling mechanisms, T-World’s CaT kinetics are similar to ToR-ORd (Figure 2A). Both show a linear CaT upstroke with a small delay relative to the AP upstroke, matching experimental recordings of simultaneous calcium and membrane potential changes.^31,32^ However, T-World achieves this through physiologically realistic calcium-mediated calcium-induced calcium release. The Morotti2021 model also has calcium-mediated calcium-induced calcium release, but manifests an unrealistically slow initial CaT rise phase due to late SR release (Figure 2A). The TP06 model manifests a very large and early peaking CaT (Figure 2A), inconsistent with human data biomarkers, and order-of-magnitude too high dyadic calcium concentrations (Figure S8).

The biphasic CaT upstroke in Morotti2021 and its predecessors arises from SR release peaking around 30 to 40 ms, compared with the ≈10 to 15 ms peak seen in T-World, ToR-ORd, TP06 (Figure S9), and experimental data.^32–35^ This delayed release not only slows early CaT rise but also affects the timing of calcium-dependent I_CaL_ inactivation, which should occur rapidly after calcium influx. Finally, late release generates depolarizing currents via NCX and I_(Ca)Cl_ at 30 to 40 ms, distorting early AP morphology. Accelerating the SR release while retaining calcium sensitivity was therefore a major goal for T-World development.

Another major development target was the cell’s response to changes in SERCA activity. Experimentally, SERCA activity is closely associated with CaT amplitude (Figure 2B), with SERCA potentiation increasing CaT amplitude and contractility,^36^ and vice versa for reduced SERCA activity.^23,37^ Capturing this relationship is key for simulating disease or sympathetic stimulation. The baseline Morotti2021 model and its predecessors show a limited agreement with data (Figure 2C and 2E), whereas the revised calcium-handling system in T-World provides good correspondence (Figure 2C and 2D).

In humans, CaT amplitude^24,25^ and contraction force^24,26–29^ rise with increasing stimulation frequency before declining at very high heart rates (Figure 2F through 2G), whereas SR calcium content increases monotonically in human^26^ and rabbit^30^ data (Figure 2H). T-World is in good agreement with these data, showing a nonmonotonic calcium- and force-frequency relationship, as well as a positive relationship between frequency and calcium concentration in the SR ([Ca]_SR_) (Figure 2I through 2K). This is nontrivial because the SR calcium load-release relationship is positive and steep in experiments^38^ and computer models. The model, therefore, requires sufficient refractoriness of I_CaL_ and RyR to counterbalance this coupling at high pacing rates.

T-World’s results quantitatively match several experimental data sets: peak calcium concentration rises to ≈150% of the 0.5 Hz level (Figure 2I and 2F), and developed force increases to ≈170%, closely matching human studies (Figure 2J and 2G). Other models show clear discrepancies: ToR-ORd exhibits, in steady state (Figure S10), a fully positive calcium–frequency relationship, while Morotti2021 shows a flat or negative calcium, force, and [Ca]_SR_ rate-dependence at rates over 1 Hz, rather resembling a heart failure phenotype.^26,28^ TP06 displays overly steep rate dependence and a biphasic SR calcium curve inconsistent with experiments (Figure S11).

I_CaL_ properties critically determine calcium handling. T-World manifests: (1) a data-like I_CaL_ current-voltage relationship (Figure S12A), which follows from (in)activation properties and maximum current amplitude, (2) a data-like recovery from refractoriness (substantially improving on ToR-ORd; Figure S12B), and (3) a good balance between voltage-dependent and calcium-dependent inactivation (Figure S12C and S12D).

Furthermore, calcium clearance during an AP is distributed as follows: SERCA 79.6%, NCX 20.15%, and pCa (sarcolemmal calcium pump) 0.25%, in agreement with data from large mammals indicating SERCA dominance, 20% to 30% clearance by NCX, and pCa involvement.^39^ Finally, further validation confirms that T-World reproduces the negative inotropic effects of sodium channel blockers and that sodium concentration changes with pacing rate qualitatively match experiments (Figure S13).

### Sympathetic Nervous System Stimulation

Excitation-contraction coupling and electrophysiology are strongly modulated by the βARS pathway, which mediates the myocardial response to sympathetic nervous stimulation. Our model includes the Heijman et al^40^ βAR description, with modifications to account for updates to ionic currents and inclusion of the contractile apparatus in the model (Supplemental Methods). We calibrated T-World to achieve APD shortening with saturating activation of βARS, subsequently validating it by comparing the model outputs to human data on βARS-induced changes in CaT and contraction.

**Figure 3.**
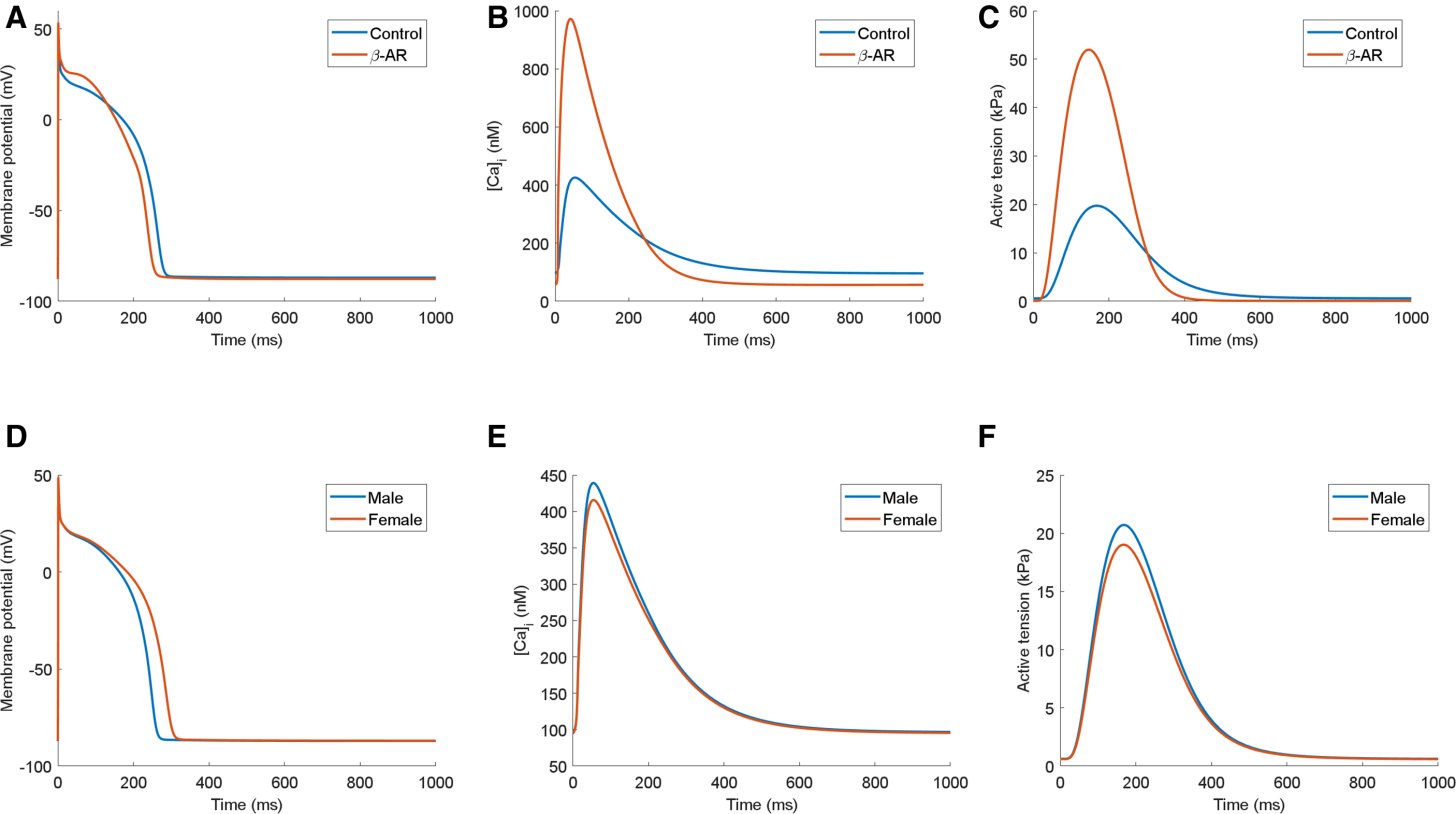
**Effect of sympathetic nervous stimulation and sex differences in T-world. A** through **C**, Action potential, calcium transient, and contractility in the absence or presence of simulated β-AR (β-adrenergic) activation. **D** through **F**, Action potential, calcium transient, and contractility in male (blue) and female (orange) model versions. [Ca]_i_ indicates cytosolic calcium concentration.

Activation of βARS in T-World causes a ≈7% APD shortening (Figure 3A), in line with human studies indicating APD shortening after isoproterenol exposure.^41,42^ The elevation of early plateau potentials in the model, resulting from the increased I_CaL_, was also consistent with data from canine cardiomyocytes exposed to isoproterenol.^43^ βARS activation induces a large 2.68-fold increase in CaT amplitude (Figure 3B), consistent with studies in humans and rabbits^44,45^ that show a several-fold increase, although direct quantitative comparison is difficult, given the uncertain mapping of calcium-induced fluorescence changes to calcium concentrations. The 24% shortening of CaT at half-amplitude is similar to the ≈30% shortening observed in human samples.^45^ Our model predicts a 2.63-fold increase in contractility (Figure 3C), which is generally in line with the highly heterogeneous human data that report an increase of 1.5-fold,^46^ 3.0-fold or 4.5-fold (depending on βARS-agonist^45^), and 5.0-fold.^47^ Changes in kinetics of upstroke and recovery of contraction are also highly heterogeneous across different studies, although there is general agreement that βARS accelerates development and recovery of contraction,^45–47^ as predicted by our model.

Similar predictions of substantially increased CaT amplitude and contraction are made by the Morotti2021 model, which also includes βARS, although APD does not shorten visibly in this model, and there is almost no plateau elevation (Figure S14).

### Sex Differences

Pronounced differences exist between hearts from females and males, which subsequently translate into differential risk of various adverse cardiac outcomes.^48^ Given the extent and importance of sex differences in cardiovascular physiology, we constructed a male and a female version of T-World, based on available experimental data and prior simulation approaches.^49–51^ The female version of T-World has a prolonged APD (Figure 3D), and a slightly smaller CaT and peak developed force (Figure 3E and 3F), in agreement with available data.^52,53^ Thus, sex-specific formulations of T-World correctly translate differences in ionic currents and buffering into key differences in overall phenotype, enabling studies into sex differences in arrhythmic risk (discussed below).

### Cellular Arrhythmic Behaviors

There are 4 key arrhythmogenic behaviors at the cellular level: EADs, alternans, DADs, and steep restitution. Although previous models have been used to simulate subsets of these mechanisms, each of the available models recapitulates only a few mechanisms with (patho)physiologically relevant parameter values, indicating major limitations in the representation of underlying physiology and predictivity. As such, in-depth assessment of cellular arrhythmic behaviors represented a major focus of the present study.

### Early Afterdepolarizations

**Figure 4.**
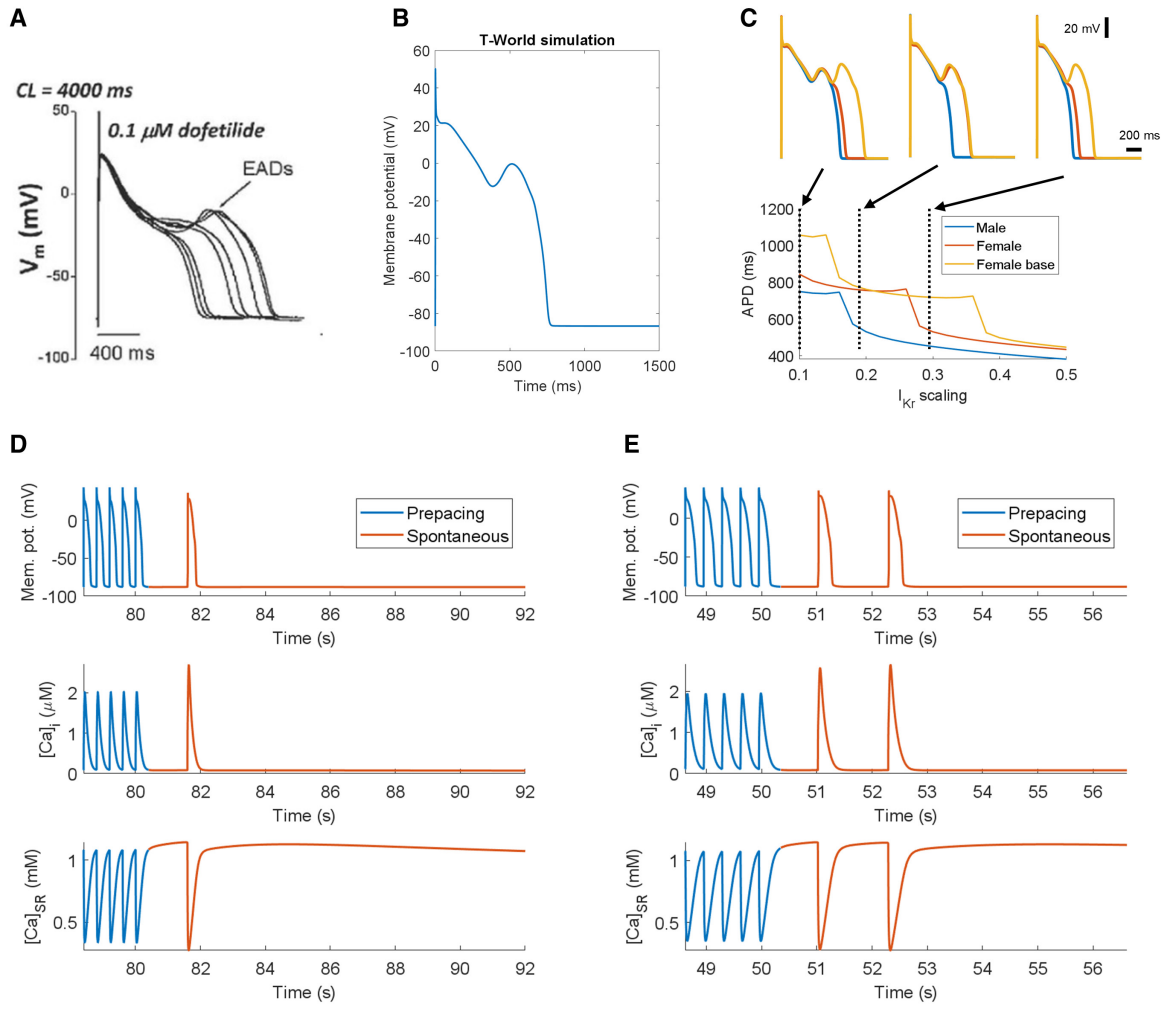
**Early afterdepolarizations (EADs) and delayed afterdepolarizations (DADs) in T-World. A** and **B**, Experimental data (**A**) and model (**B**) showing EADs at 0.25 Hz pacing, with 85% block of rapid delayed-rectifier potassium current (I_Kr_) via dofetilide.^54^ **C**, Demonstration of differential EAD formation under varying degrees of I_Kr_ availability in male and female model versions, as well as female+increased L-type calcium channel (I_CaL_), reflecting the basal part of the female heart in a rabbit study.^55^ The *y* axis shows action potential (AP) duration for a range of I_Kr_ scaling factors (fraction of current vs baseline) on the *x* axis, with sharp transitions corresponding to changes in the number of EADs. Insets show APs at corresponding dashed lines. **D**, Examples of triggered activity resulting from DADs (prepacing for 200 beats at 2.5 Hz, with fully active β-adrenergic signaling and extracellular calcium of 3.25 mmol/L). The end of the prepacing train is shown in blue, with the spontaneous activity given in red. **E**, Similar to **D** with 3 Hz, rather than 2.5 Hz prepacing rate for 150 beats, showing the generation of multiple triggered APs. [Ca]_i_ indicates cytosolic calcium concentration; [Ca]_SR_, calcium concentration in the SR; CL, cycle length; and Mem. pot., membrane potential. Figure 4A is used from O'Hara et al,^18^ which published under CC BY 4.0.

EADs, extrasystolic depolarizations during an AP, contribute to arrhythmogenesis and are commonly linked to drug-induced cardiotoxicity and long QT syndromes, being typically driven by the reactivation of I_CaL_ during prolonged APD^13^ (Figure 4A). T-World replicates EADs under realistic conditions of drug-induced long QT (Figure 4B), similar to ToR-ORd and ORd models,^4,18^ with a 13-mV amplitude, similar to experimental observations.^54^ At the same time, the good EAD-capability of T-World is not straightforward, given considerably greater and more data-like refractoriness of I_CaL_ compared with predecessors (Figure S12B). In contrast, the TP06 model requires nearly tripled I_CaL_ to manifest EADs,^56^ likely due to excessive I_Ks_ providing strong repolarization reserve. The Morotti2021 model^11^ similarly requires a 2.5-fold I_CaL_ to induce EADs (Figure S15).

T-World also highlights sex differences in EAD vulnerability. The female T-World variant requires less I_Kr_ inhibition to induce EADs compared with the male variant, indicating greater EAD vulnerability (Figure 4C), supporting data showing higher risk of drug-induced arrhythmia in female hearts.^49,57^ In addition, female-specific apicobasal I_CaL_ gradients linked to estrogen^58^ suggest an additional risk of EADs in basal regions of the heart in female models. In agreement, T-World shows increased EAD susceptibility in the female model version with increased I_CaL_ (Figure 4C).

### Delayed Afterdepolarizations

DADs are arrhythmia triggers occurring during diastole. They result from spontaneous SR calcium release, generating inward currents (primarily via NCX) that depolarize the cell^59^ and are particularly prominent in diseased hearts, for example, in heart failure.^60^ DADs arise from stochastic subcellular calcium sparks best simulated by models with spatial calcium handling and stochastic gating.^61^ However, given the high computational cost of such models, common pool models with similar complexity as T-World, especially Bers/Grandi–like models, such as Morotti2021,^9–11^ are often used to emulate DAD generation efficiently. By contrast, ToR-ORd cannot produce any DADs due to its RyR activation mechanism, whereas TP06 can yield DADs after parametric changes, but these differ substantially from experimental recordings.^62^

T-World manifests spontaneous calcium releases and DADs, and it can generate DAD trains, as observed in certain experiments^63^ (Figure 4D and 4E). Spontaneous releases are terminated when the SR content becomes sufficiently low after the spontaneous releases, similar to simultaneous measurements of intracellular and SR calcium.^64^ In this regard, our model differs from Morotti2021, where DADs stop occurring even when SR calcium keeps increasing (Figure S16).

To validate DADs in the model, we confirmed that faster prepacing and RyR sensitization promote DADs in T-World, as seen experimentally (Figure S17). Furthermore, for applications requiring stochasticity of DADs, we developed a version of T-World that includes stochastic store-overload-dependent RyR release akin to the method by Colman et al^65^ (Figure S18).

### Calcium and AP Alternans

Cardiac alternans, a periodic oscillation between long and short APDs, creates a proarrhythmic substrate, promoting conduction block^66^ and increasing arrhythmia risk.^67^ APD alternans is typically driven by underlying CaT oscillations and occurs at rapid heart rates.^68^ Although common at high pacing rates in living hearts, many computer models do not recapitulate it readily, including the Bers/Grandi family, which provided the basis for the T-World calcium handling.

**Figure 5.**
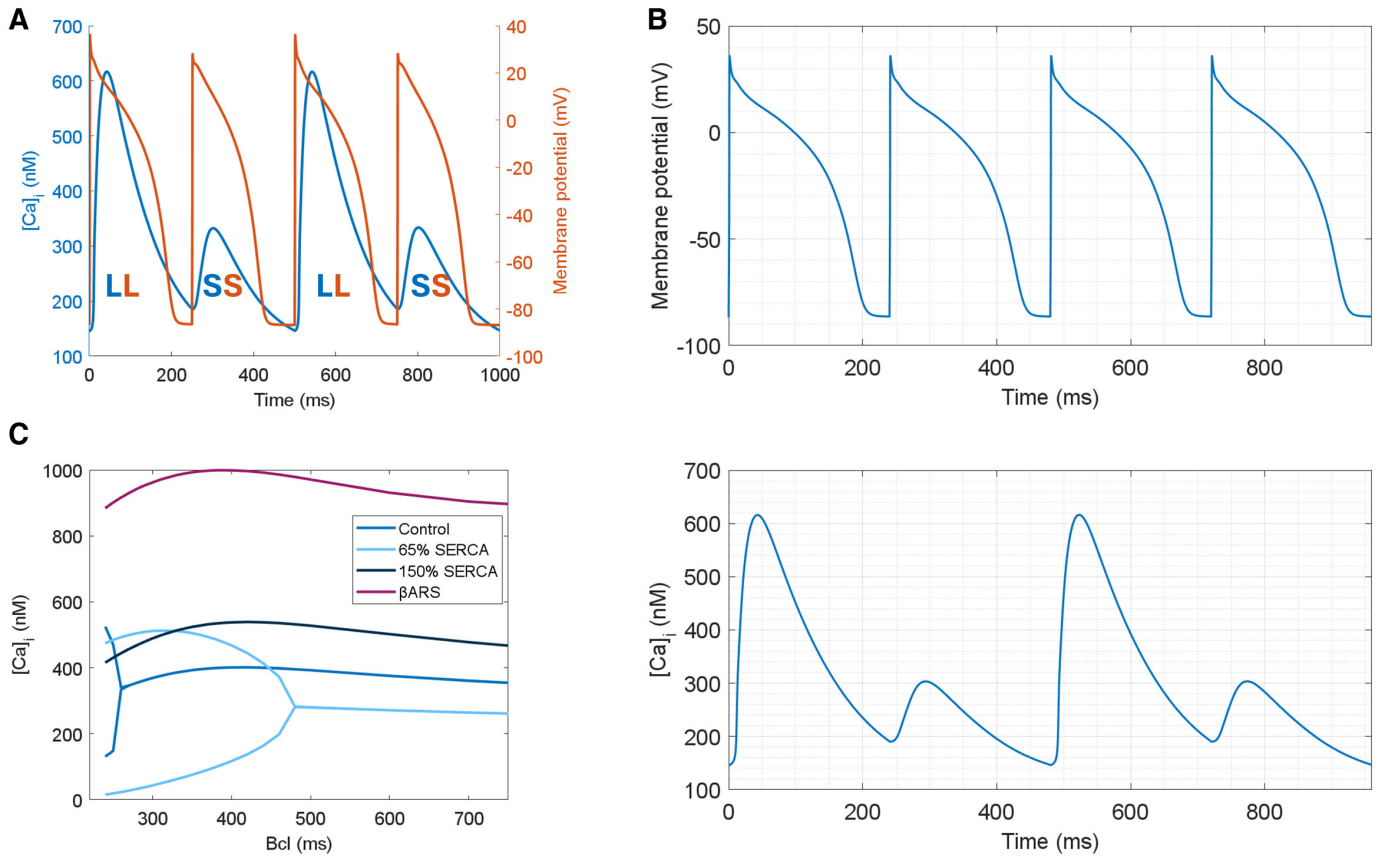
**Alternans in T-World. A**, Illustration of concurrent oscillations in calcium transient ([Ca]_i_, blue) and action potential (AP) duration (orange). **B**, Calcium alternans in T-World under AP clamp based on a fixed-shape AP applied 250× at a basic cycle length (bcl) of 240 ms. **C**, Modulation of calcium alternans by reduced or increased SERCA (sarco/endoplasmic reticulum Ca^2+^ ATPase) activity, as well as by βAR (beta-adrenergic) stimulation. [Ca]_i_ indicates cytosolic calcium concentration; LL, large/long calcium transient and AP duration, respectively; and SS, small/short.

Thanks to its improved calcium handling, T-World produces AP and CaT alternans at realistic frequencies,^69^ with mild alternans at 260 ms and pronounced alternans at 240 to 250 ms (Figure 5A; Figure S19). Alternans is electromechanically concordant (long APD corresponds to large CaT), matching experimental data in human-relevant species.^30,70,71^ T-World shows CaT alternans even when a fixed AP shape is imposed, confirming calcium oscillations as the primary driver, rather than AP restitution (Figure 5B).

A major improvement of T-World compared with prior state-of-the-art is the correct response in alternans properties to SERCA pump changes. Consistent with conditions like heart failure and pharmacological or transcriptional SERCA reduction,^72–74^ T-World shows increased alternans vulnerability with SERCA reduction, with alternans appearing at slower pacing rates (Figure 5C). This is in contrast with ToR-ORd, where SERCA inhibition suppresses alternans (Figure S20). Finally, we validated T-World by showing that an increase in SERCA function or βAR activation suppresses alternans (Figure 5C), in line with experimental data.^75,76^

### Steep S1-S2 Restitution

**Figure 6.**
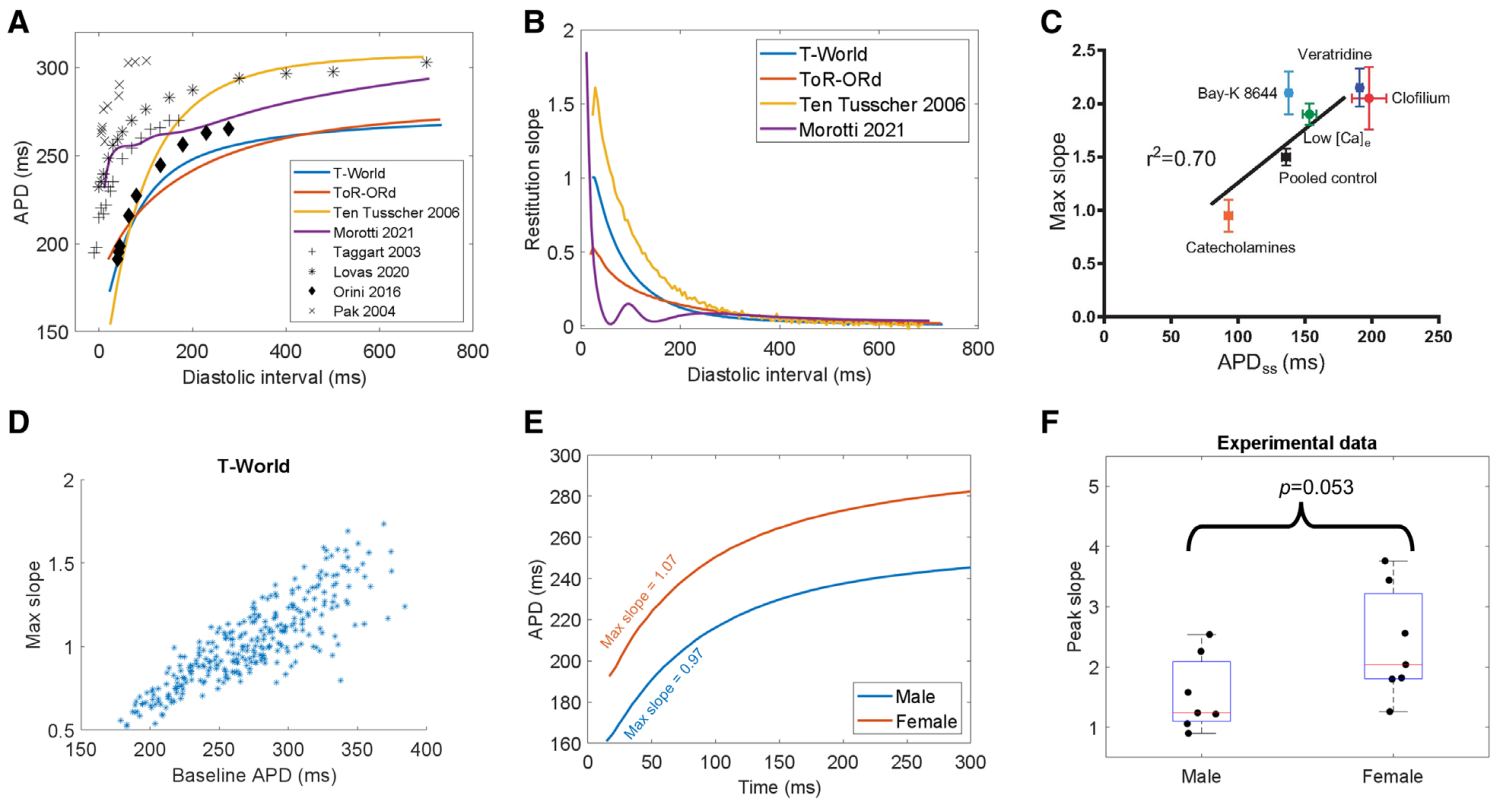
**S1-S2 restitution. A**, S1-S2 restitution curve in the T-World, ToR-ORd, Morotti2021, and Ten Tusscher-Panfilov 2006 model (TP06) models, and a range of human studies.^78,80–82^ **B**, Comparison of S1-S2 restitution slopes across T-World, ToR-ORd, Morotti2021, and TP06 models. **C**, Positive relationship between AP duration (APD) of a cell and its peak S1-S2 restitution slope, observed experimentally.^16^ **D**, Corresponding simulation in T-World showing that when a range of ion channel conductances are varied (see Methods for details), cells with longer APD generally show a steeper slope of restitution. **E**, Steepening of restitution in female vs male myocytes in baseline T-World. **F**, Experimental human data comparing peak restitution slope in males vs females (n=7 in both groups, *P* value obtained using Mann-Whitney *U* test). APD_SS_ indicates steady state APD; and low [Ca]_e_, low extracellular calcium. Figure 6C is used from Shattock et al,^16^ which published under CC BY 4.0.

Steep APD restitution (ie, a slope of the restitution curve >1) promotes arrhythmias by facilitating reentry and spiral wave breakup in cardiac tissue.^12,77^ Human studies show that maximum curve slopes slightly above 1 are not uncommon.^78,79^ The TP06 model has been historically popular because of its steep restitution properties, whereas, for example, the ToR-ORd model, on which most of T-World’s electrophysiology is based, has a relatively flat restitution (peak slope ≈0.5). However, our revised I_CaL_ and other developments lead T-World to exhibit good agreement with experimental restitution data (Figure 6A), with an S1-S2 slope >1 for a subset of S2 intervals at an S1 interval of 1000 ms (Figure 6B). Restitution of APD is paralleled by the restitution of conduction velocity (Figure S21).

Shattock et al^16^ have shown that the maximum slope of the restitution curve is largely determined by the steady-state APD of a cell. Specifically, the longer the APD, the steeper the restitution (Figure 6C), which was corroborated by multiple experimental studies using different means of changing APD.^83–85^ Importantly, T-World is the only model among those capable of steep restitution that recapitulates this feature (Figure 6D), with the TP06 model showing a weakly inverse APD-slope relationship, and the Morotti2021 a strongly inverse one (Figure S22). This makes T-World uniquely suitable for studying how APD changes due to disease or drugs modulate arrhythmic risk via restitution changes.

One notable exception to the observation by Shattock et al^16^ is the effect of βAR stimulation, which shortens APD but steepens the S1-S2 slope in humans.^78^ As an independent validation, we simulated the effect of βAR activation in the T-World model, which produced the correct phenotype (Figure S23). Furthermore, we validated that shortening of the S1 interval indeed flattens the S1-S2 restitution (Figure S24).

Measuring restitution slope separately for the male and female versions of T-World, we observed a steeper slope in the female myocyte (Figure 6E; Figure S25). This would point to an increased risk of arrhythmia in female hearts through steeper restitution, but intriguingly, we were unable to find any experimental study addressing this hypothesis. However, we were able to obtain human ventricular data from the study by Árpádffy-Lovas et al^80^ and reanalyzed them for sex differences in peak slope. Mean (± standard deviation) peak slope in males was 1.54 (±0.63), increasing to a mean of 2.38 (±0.92) in females (*P*=0.053, Mann-Whitney *U* test, Figure 6F). This suggests that females may have steeper restitution properties, a previously underappreciated sex-specific hazard.

### Stability of Arrhythmic Behaviors

**Figure 7.**
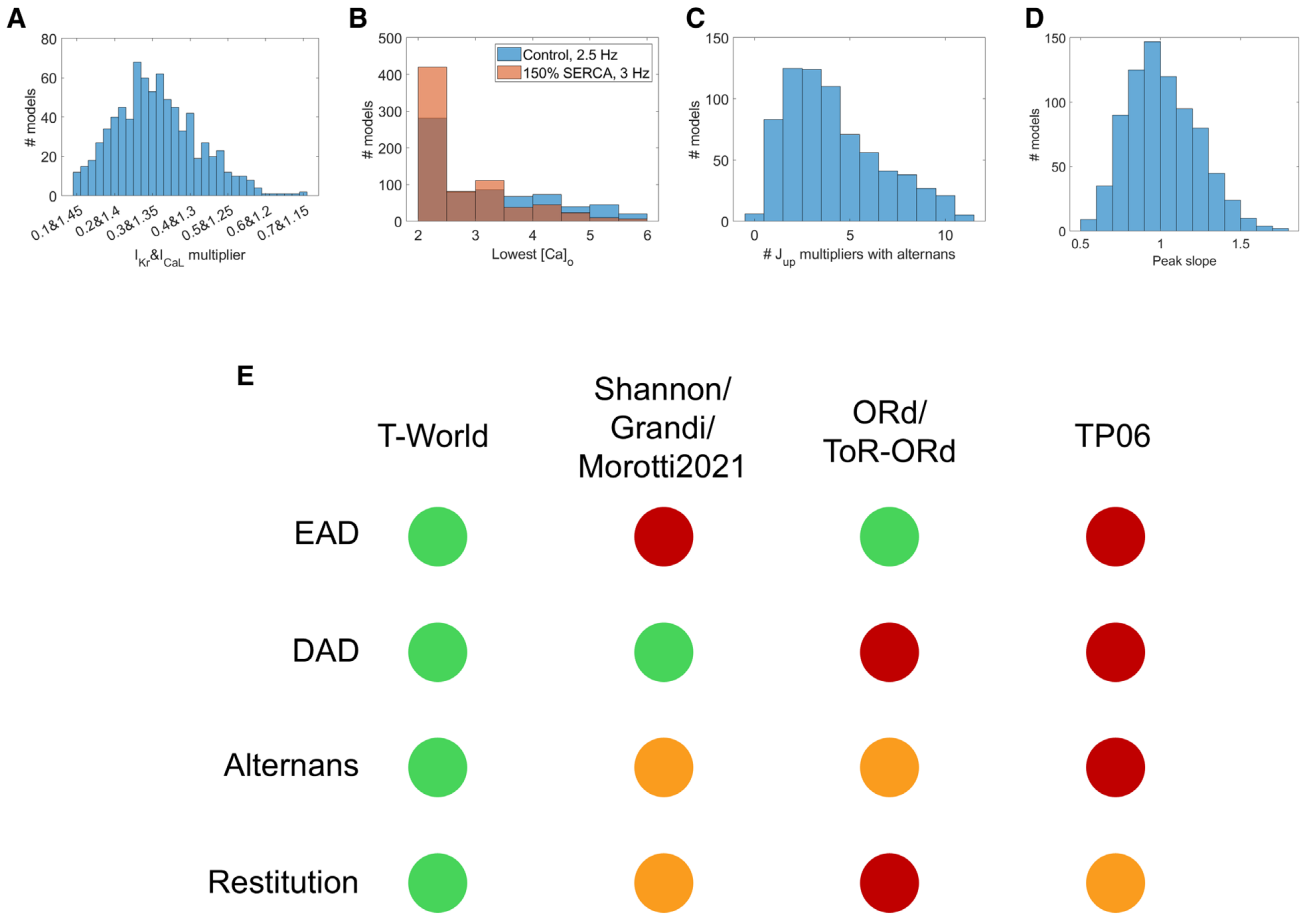
**Stability of arrhythmic behaviors in T-World. A**, Histogram describing the degree of pro-early afterdepolarization (EAD) alteration to the baseline cell, increasing L-type calcium current (I_CaL_) and reducing rapid delayed rectifier potassium current (I_Kr_), that is sufficient to trigger EADs in each model in a calibrated population of models (see Arrhythmia Studies in Supplemental Methods for details). **B**, Histogram of the level of extracellular calcium ([Ca]_o_) that is sufficient to trigger delayed afterdepolarizations (DADs) after rapid prepacing in the presence of βARS (beta-adrenergic signalling) stimulation in the baseline T-World model and a version with 50% higher SERCA (sarco/endoplasmic reticulum Ca^2+^ ATPase) activity. **C**, Histogram showing how many models among those with J_up_ multipliers of 0.5, 0.6, …, 1.5 (11 in total) manifest alternans at 260 ms basic cycle length. **D**, Distribution of peak S1-S2 slopes in the calibrated population. **E**, Visual comparison of T-World and prior modeling families, depicting whether an arrhythmogenic behavior is stable and satisfactory (green), present in principle with caveats (eg, requiring substantial parameter changes or via unphysiological mechanisms; orange), or almost completely or completely absent (red). See Figure S27 for further details on simulations and criteria underlying the diagram. J_up_ indicates SR calcium uptake flux.

To validate the generality and robustness of T-World, we investigated the stability of the cellular arrhythmic behaviors under parameter perturbation using a population-of-models approach with key ionic currents and fluxes varied between 67% and 150%. Although it is natural for cells, living and simulated alike, to manifest arrhythmogenic behaviors under slightly different conditions, the majority of cells should be fundamentally capable of manifesting them. Overall, 786 out of 1000 models passed all the calibration criteria derived from human data (Supplemental Methods for details) and were used subsequently. In total, 99.5% of the 786 models manifest EADs for a sufficient I_Kr_ and I_CaL_ perturbation, while 88% manifest DADs for a sufficiently high extracellular calcium, increasing to 93.4% when increasing SERCA by 50% to facilitate SR calcium overload (Figure 7A and 7B). The key factor preventing DADs in a part of the population is increased NCX current I_NaCa_ (Figure S26) because this can clear released calcium more rapidly from the dyadic space, which limits the pro-DAD condition of high dyadic calcium combined with high [Ca]_SR_ load, as also observed previously.^86^ In total, 708 models in the population can capture all stimuli at a cycle length of 260 ms, and 99% of these manifest alternans for at least 1 tested SERCA multiplier (Figure 7C). Finally, 48.5% of models exhibit a restitution slope > 1 (Figure 7D), consistent with 44%,^79^ 74%,^87^ and 61%.^81^ of samples in human recordings. Thus, T-World is highly robust with regard to its arrhythmia precursor capabilities, which are its intrinsic properties, rather than phenomena that only occur for highly specific sets of distinct parameters for each property. This is a fundamental advance compared with prior major models, which show a limited range of arrhythmogenic behaviors, and often with major caveats (Figure 7E).

## Discussion

We developed, calibrated, and validated T-World, a new computer model of the human ventricular cardiomyocyte designed to meet the longstanding need for a highly general human-specific virtual cardiac cell. Integrating innovations with carefully curated elements from the Rudy-like^4,18^ and Grandi and Bers^9,10^ models, T-World unifies these 2 major model families, combining their strengths while resolving key limitations. Unlike predecessor models with limited arrhythmogenic capabilities, it reproduces, in a single model, the generation of EADs and DADs, realistic restitution dynamics, and calcium-driven alternans responsive to SERCA modulation.

The model’s credibility is supported by extensive calibration to human data (Table S1), independent validation on unseen data (Table S2), and construction from well-characterized components. Unlike machine learning models with billions of free parameters, T-World is built from components (ionic channels and signaling pathways), which were derived from experimental measurements, markedly reducing the risk of model overfitting and consequent incorrect predictions. Its universality and adaptability make it valuable for basic research, drug testing, and patient-specific virtual twins.^88^

T-World advances the 3Rs principles: particularly reduction and (partial) replacement of animal use. It is synergistic with in vitro systems such as induced pluripotent stem cell–derived cardiomyocytes, helping interpret their immature phenotypes in an adult-heart context. Its human-specificity also enables the translation of animal data, such as protein-level changes, into predictions of functional implications for human physiology. There are important differences between the hearts of humans and animals, particularly the most popular experimental animal models such as mice and rats.^89^ For example, most episodes of drug-induced arrhythmia in humans are due to drug-induced inhibition of hERG (human Ether-à-go-go-Related Gene) channels,^90^ but this channel is nearly absent in the hearts of mice and rats. As such, testing the safety of drugs in those species is of limited use, as those species lack the key component responsible for the issue. We note that in this context, human specificity refers predominantly to the phenotype produced and focuses on using human data in calibration and validation. Although the model utilizes human data to describe the kinetics of ionic channels and other processes wherever possible, some model components are based on prior animal-based formulations, as human data are absent.

T-World represents sex differences in cardiac cell physiology, and its simulations, supported by newly analyzed human data, suggest that females may be more prone to steep restitution slopes. This is in addition to higher EAD susceptibility observed here and previously,^49,57^ with both mechanisms promoting arrhythmia at the tissue level.^12,77^ The finding of likely steeper restitution in females highlights T-World’s potential to generate biological insights but warrants confirmation in larger studies. The results reinforce the need for sex-specific drug dosing and treatment guidelines, which remain insufficiently addressed.^91^

T-World’s strong calcium handling and excitation-contraction coupling make it well-suited for studying diseases with pronounced calcium remodeling, such as heart failure and postinfarction remodeling. Unlike models like ToR-ORd, T-World can produce DADs, important in such diseases.^60^ A particular strength pertaining to arrhythmia mechanisms is that T-World exhibits increased alternans vulnerability with a reduction in SERCA activity, both hallmarks of those diseases. This is an improvement over major prior human models such as ORd and ToR-ORd,^4,18^ which do not respond well to SERCA changes, and are thus less suitable for modeling this aspect of disease.

Incorporation of βAR signaling and excitation-contraction coupling modulation makes T-World particularly useful for studies of the neurocardiac axis and sympathetic control of arrhythmia.^92^ Validation results demonstrate strong predictive performance for multiple arrhythmic mechanisms influenced by sympathetic activation, underscoring its utility for mechanistic and translational research. As more data become available on electrophysiological and signaling effects of sympathetic neurotransmitters such as Neuropeptide Y, it will be interesting to incorporate these, given their proarrhythmic role.^93^ Incorporation of parasympathetic signaling is another likely direction of development.

The main limitation of computer models like T-World is that they can only reproduce behaviors that emerge from the components explicitly included. Yet this limitation can serve as a powerful research tool. When a model’s predictions diverge from experimental observations, the discrepancy can highlight gaps in our biological understanding, pointing, for example, to a missing signaling pathway that may contribute to the observed phenomenon. Such omissions might explain, for instance, the lack of APD shortening in response to elevated extracellular calcium in T-World (Supplemental Note 1).

A known limitation of models at this level of abstraction is their inability to accurately represent DAD origins and the spatial heterogeneity of subcellular calcium handling. DADs arise from localized spontaneous calcium releases that spread diffusively through the cell, recruiting additional RyRs. Because T-World and similar models describe only average calcium concentrations per compartment, they cannot capture this within-compartment diffusion process. Likewise, it is challenging to represent certain changes in subcellular structure, such as changes in T-tubule density or dispersion of RyRs, important, for example, in heart failure.^94^ Spatially distributed models, which represent thousands of RyR and I_CaL_ clusters can simulate calcium wave propagation and T-tubule structure more realistically^95^ and can provide more detailed insights into the subcellular determinants of calcium-driven alternans that are present at a whole-cell scale in T-World.^96^ At the same time, they are computationally demanding (up to an hour per beat, as opposed to ≈0.15s in T-World) and less scalable, occupying a different niche with regard to applications. This makes developing hybrid approaches that incorporate spatial diffusion into T-World–like frameworks an interesting direction for future research.

On the other end of the model complexity spectrum, while T-World is suitable for 3-dimensional organ-scale simulations (as demonstrated in the second article), considerably simpler models may enable more high-throughput studies focused on conduction properties, where cellular realism is not a priority. Such models include, for example, the TP06 model also investigated here,^12^ or the even simpler and more computationally efficient minimal ventricular model by Bueno-Orovio et al.^97^

The versatility and broad applicability of T-World open new avenues in cardiac research and predispose it to further extensions, such as variants representing disease remodeling, channel mutations, or investigating nonstandard ionic channels. We envision that its universality, modularity, and open-source nature will also enable adaptations to represent other excitable cells, such as atrial, sinoatrial, Purkinje, or neuronal. It will also facilitate studies on newly discovered ionic currents, and on understanding signaling pathways and how they modulate cellular physiology. To expand the model’s generality, we anticipate it will be particularly important to represent dynamic regulation of trafficking and transcription,^98^ enabling studies on long-term remodeling, representation of mitochondria, metabolism, and reactive oxygen species,^99^ integration with AI-driven structural modeling,^100^ and omics analyses.^101^

To support continued development while preserving reproducibility, we will treat T-World as a versioned, openly released framework. Incremental extensions (eg, adding a mutation or a narrowly targeted remodeling mechanism) can typically be implemented modularly without affecting baseline behavior, whereas more disruptive changes to central subsystems (eg, calcium handling) will warrant revalidation using the same calibration and validation criteria established here. Each public release will be assigned a distinct version number (current: v1.0) and accompanied by a transparent changelog and archived code snapshot, enabling users to reproduce published results and to identify precisely what changed between versions. Importantly, future refinements will be evaluated against the current model’s defining behaviors and predictive benchmarks; any trade-offs introduced by extensions will therefore be detected explicitly and reported, ensuring that improvements do not inadvertently compromise the capabilities demonstrated in this article.

## ARTICLE INFORMATION

### Acknowledgments

The authors thank Eleonora Grandi, Stefano Morotti, and Haibo Ni for useful discussions on how models derived from Shannon et al operate. The authors also thank Dirk Gillespie, Dezso Boda, Pavel Jungwirth, and Geir Halnes for their insights on how ionic driving force through open L-type calcium channels should or should not be modeled, and Roshni Shetty and David Ortega for spotting minor issues in the code.

### Author Contributions

J. Tomek conceptualized and coordinated the study, designed, developed, and validated the model with contributions from J. Heijman, D.M. Bers, M.A. Colman, and A. Bueno-Orovio. J. Heijman, D.M. Bers, and B. Rodriguez jointly supervised the project. J. Tomek and J. Heijman wrote the initial draft, subsequently revised by D.M. Bers, B. Rodriguez, M. Tomkova, A. Bertrand, M.A. Colman, and A. Bueno-Orovio. M. Holmes developed the formulation of sex differences and performed conduction velocity restitution simulations. T. Bury developed the online application to run T-World. M. Tomkova contributed to genetic algorithm fitness design, visualization, and modeling design choices. H. Jo generated codes to assess sodium-potassium pump function and contributed to its redesign. N. Nagy provided and analyzed experimental data on sex differences in S1-S2 restitution.

### Disclosures

None.

### Supplemental Material

Supplemental Methods

Tables S1–S6

Figures S1–S27

Major Resources Table

References 102–167

## Supplementary Material


