# Supplementary material for: T-World Virtual Human Cardiomyocyte. I. Development, Validation, and Cell Arrhythmogenesis
Source: Circ Res. 2026 Apr 7;138(10):e328073. doi: 10.1161/CIRCRESAHA.125.328073 (PMC13152079; doi:10.1161/CIRCRESAHA.125.328073)
Supplement: Supplementary file 1 [file res-138-e328073-s001.pdf]

Supplemental Material:
T-World virtual human cardiomyocyte. I. Development, validation, and cell arrhythmogenesis

Jakub Tomek, Maxx Holmes, Thomas Bury, Marketa Tomkova, Heeseung Jo, Norbert Nagy, Ambre Bertrand, Alfonso Bueno-Orovio, Michael A. Colman, Blanca Rodriguez, Donald M. Bers, Jordi Heijman

Table of contents

Supplementary Methods 3
- Model architecture 3
- Calibration and validation criteria 3
- Ionic currents and fluxes 6
  - Sodium current (I_{Na}, I_{NaL}) 7
  - L-type calcium current (I_{CaL}) 8
  - Transient outward current (I_{to}) 10
  - Rapid delayed rectifier current (I_{Kr}) 11
  - Slow delayed rectifier current (I_{Ks}) 11
  - Inward rectifier current (I_{K1}) 12
  - Sodium-calcium exchanger (I_{NaCa}) 12
  - Sodium-potassium pump (I_{NaK}) 13
  - Chloride currents ($I_{(Ca)Cl}$, I_{Clb}) 14
  - Background currents (I_{Nab}, I_{Cab}, I_{Kb}) 14
  - Stimulus current (I_{stim}) 14
  - Calcium release from the SR (J_{rel}, J_{leak}) 14
  - Calcium reuptake to the SR (J_{up}) 17
  - Sarcolemmal calcium pump (p_{Ca}) 18
  - Buffering and ion diffusion between compartments 18
- Contraction modelling 19
- CaMKII signaling 20
- βAR signaling 21
- Sex differences 22
- Arrhythmia studies 23

EADs 23
DADs and stochastic DADs 23
Alternans 24
Restitution 24
Stability of arrhythmic behaviors 24
Graphical user interface 25
Parameter optimization using genetic algorithms 25
Details of optimization fitness 27
Fitness for calcium handling 28
Fitness for I_{CaL} and overall model behavior 32
Notes on implementation 35
Other models 36
Experimental methods 36
Supplementary notes 36
Supplementary note 1: Extracellular calcium and APD 36
Supplementary figures 38

Supplementary Methods

Model architecture

The overall cell architecture and compartmentalization of T-World (**Figure 1** of the main manuscript) is based on the architecture introduced in the Shannon et al. rabbit model [9]. We retained the compartment volumes and localizations of buffers. The majority of ionic currents and fluxes were replaced by alternative formulations, as described below.

A substantial update is that intracellular potassium and chloride concentrations are dynamic in T-World, being updated according to ionic currents, unlike the Shannon model and later models in the same family (e.g. [10,11]), where calcium and sodium were dynamic, but potassium and chloride were at fixed concentrations. Given the relatively uniform concentrations of potassium and chloride in a cardiomyocyte, we represent only a single intracellular concentration of those ionic species to keep the model simple and efficient. By contrast, sodium and calcium have separate concentrations in dyad, subsarcolemmal (SL), and myoplasm (MYO) compartments. Most ionic currents are localized similarly to Shannon-like models, with 89% localized in the SL compartment, with 11% placed in the dyad. The exceptions are I_{CaL} (80% in dyads), I_{NaCa} (31% in dyads), and $I_{(Ca)Cl}$ (50% in dyads). For potassium and chloride currents, the reversal potential was calculated based on the extracellular concentration and the single, homogeneous intracellular concentration.

The model has three versions according to transmural localization: endocardial, epicardial, and midmyocardial. The endocardial model is used as a default version throughout the article unless stated otherwise. Finally, T-World uses a conservative potassium stimulus (pacing stimulus current produces appropriate change in ionic concentration) [102], improving model stability compared to nonconservative-stimulus models.

Calibration and validation criteria

T-World was developed and calibrated to reproduce a wide range of pre-defined behaviors (**Table S1**), which was followed by validation on data not used in development (**Table S2**). The behaviors were chosen to reflect key properties of myocyte physiology and excitation-contraction coupling, and their modification by treatments and conditions with a known effect. Among the calibration criteria, we note that the criterion on alternans being promoted by SERCA reduction was fulfilled spontaneously once we were able to develop the Ca handling model to recapitulate alternans, without the development process looking at this feature. While it was included as a calibration criterion in later development stages to make sure the property is not lost, the fact that it emerged spontaneously serves as semi-validation.

On the other hand, the criterion on APD-slope relationship being positive was initially included as a validation criterion in the T-World development (APD = action potential duration). However, this was failed by a model we considered near-final several years back, which warranted investigation of the issue and consequent replacement and redevelopment of the I_{CaL} model. Given that this criterion was ultimately used in the model development, it is listed as a calibration criterion.

Table S1. Calibration criteria of T-World. *AP = action potential, APD = action potential duration, SR = sarcoplasmic reticulum, EAD = early afterdepolarization, EAD = early afterdepolarization, DAD = delayed afterdepolarization.*

Feature	Data	Method of assessment
AP morphology	[18]	Membrane potential within 10-90 percentile of the data after 15 ms (higher AP peak is allowed in the single cell model due to lack of electrotonic coupling which is

		present in reference data based on small tissue samples); shape visually consistent with experimental recordings
Calcium transient morphology	[19]	45-55 ms time to peak, 370-460 ms, duration at 90% amplitude recovery, 325-375 nM amplitude.
Dyadic calcium time to peak near 10 ms	[32–35]	Direct comparison of time to peak.
Contraction biomarkers	[20]	290-380 time from peak to 95% recovery of active tension, 147-172 ms time to peak active tension, 15-25 kPa peak active tension.
I_{CaL} I-V relationship	[103]	Root mean squared error of simulation versus data.
I_{CaL} recovery from refractoriness	[104]	Root mean squared error of simulation versus data.
Positive correlation between CaT amplitude and SERCA levels	[23,36,37,105]	Slope of CaT amplitude between 50 and 150% SERCA model is positive and at least 0.2.
Biphasic rate dependence of CaT	[24,25]	CaT amplitude increases from 1 Hz to 2 Hz, and then at a certain point between 2 Hz and 3 Hz it peaks and starts decreasing.
Biphasic rate dependence of developed force	[24,26–29]	Developed force increases from 1 Hz to 2 Hz, and then at a certain point between 2 Hz and 3 Hz it peaks and starts decreasing.
Monotonically positive rate-dependence of calcium in the SR	[26,30]	SR calcium increases from 1 Hz to 2Hz and from 2 Hz to 3 Hz.
β-adrenergic activation shortens APD	[41,42]	Direct comparison of APD without and with β-adrenergic stimulation.
EADs occur in experiment-like conditions	[54]	EAD presence at 0.25 Hz, 15% I_{Kr}, 2 mM Ca_o
DADs can be evoked in the presence of β-agonist and elevated extracellular calcium	[106]	Testing whether DADs can be evoked with full β-adrenergic activation and at Cao up to 6 mM.
Alternans occurs at human-like rates	[69]	Alternans is present around 4 Hz, but not at 3 Hz.
Alternans coupling between APD and CaT amplitude is positive	[30,70,71]	Positive correlation between APD and CaT amplitude during alternans
Alternans is potentiated by SERCA reduction and in diseases with reduced SERCA expression, and is inhibited by SERCA increase and β-AR stimulation (which increases SERCA)	[69,73–75,107–111]	Direct qualitative assessment across tested conditions.
S1S2 restitution has data-like shape	[78,83–85]	Restitution slope increases smoothly as the diastolic interval shortens.
Peak slope of S1-S2 restitution exceeds 1 slightly	[78,79,84,85,87]	Direct assessment.
Relationship between cellular APD and its peak restitution slope is positive	[16]	In a population of models, it holds on average that APD prolongation increases the peak restitution slope.

Table S2. Validation criteria of T-World. *NCX = sodium-calcium exchanger, β-AR = β-adrenergic.*

Feature	Data	Method of assessment
Timing of I_{CaL} voltage-dependent inactivation, as well as combined voltage- and calcium-dependent inactivation, is similar to data.	[18]	Root mean squared error versus data
Appropriate rate-dependent response of APD to channel blockers (E-4031, HMR-1556, nisoldipine, mexiletine)	[18]	APD changes in response to drugs within error bars of experimental data, similar slope of rate-dependence to the experiments.
Negative inotropy of sodium blockers	[112–114]	Combined 50/50% blockade of I_{Na} and I_{NaL} reduces calcium transient amplitude and thus contractility.
Positive rate-dependence of $[Na]_i$	[115]	Intracellular sodium concentration increases as pacing rate is increased from 1 Hz to 2 Hz and from 2 Hz to 3 Hz.
During excitation-contraction coupling, most calcium is removed from cytosol by SERCA pumps, 20-30 by NCX, with a minimal contribution from pCa (sarcolemmal calcium pump)	[39]	Direct comparison of integral of calcium transported.
Longer APD with slightly smaller CaT amplitude and contractility in female vs male cardiomyocytes	[52,53,116,117]	Qualitative comparison between male and female model version.
Female myocytes are more vulnerable to EADs than male cardiomyocytes	[49,57,118,119]	Direct assessment of whether female myocytes show EADs for a smaller degree of I_{Kr} inhibition.
β-AR stimulation markedly increases CaT amplitude	[44,45]	At least 2fold increase in CaT amplitude with β-AR stimulation.
β-AR stimulation markedly increases contractility	[45–47]	At least 2fold increase in peak active tension with β-AR stimulation.
β-AR stimulation steepens restitution	[78,120]	Direct comparison of peak slope in a model without versus with β-AR stimulation.
β-AR stimulation inhibits alternans	[75,121]	Direct comparison of peak slope in a model without versus with β-AR stimulation.
Shortening S1 flattens S1-S2 restitution slope	[78]	Direct comparison of peak slope evoked at S1 = 1000, 600, and 400 ms.
Faster prepacing promotes DAD formation	[122,123]	Direct comparison of how much Ca_o needs to be elevated to produce DADs at faster versus slower pre-pacing rates.
RyR sensitization promotes DAD formation in the presence of β-AR stimulation	[124]	Increase in RyR opening sensitivity to calcium should produce DADs at lower Ca_o than the control model

Ionic currents and fluxes

This section summarizes the formulations of ionic currents and fluxes in T-World. Baseline conductances are given in **Table S3**.

Table S3. Conductances and transport rates of ionic currents and calcium handling fluxes in T-World.

Parameter	Maximum conductances and transport rates
G_{Na}	22.08788 (mS/μF)
G_{NaL}	0.04229 (mS/μF)
p_{CaL}	1.5768e-04 (cm/s)
G_{to}	See corresponding section below
G_{Kr}	$0.043 \cdot \sqrt{\frac{K_o}{5}}$ (mS/μF)
G_{Ks}	Calcium-dependent, see section below. Given in (mS/μF)
G_{K1}	0.6992 (mS/μF)
G_{NaCa}	0.00179 (mS/μF)
$\bar{\mathbf{I}}_{\mathbf{NaK}}$	2.10774 (μA/μF)
G_{PCa}	0.02064 (μA/μF)
$G_{(Cl)Ca}$	0.01615 (mS/μF)
G_{Clb}	0.00241 (mS/μF)
G_{Nab}	0.000594 (mS/μF)
G_{Cab}	5.15575E-04 (mS/μF)
G_{Kb}	0.010879 (mS/μF)
$V_{max,SERCA}$	0.00543 (mM/ms)

Traces of key ionic currents at 1 Hz stimulation are given **in Figure S1**.

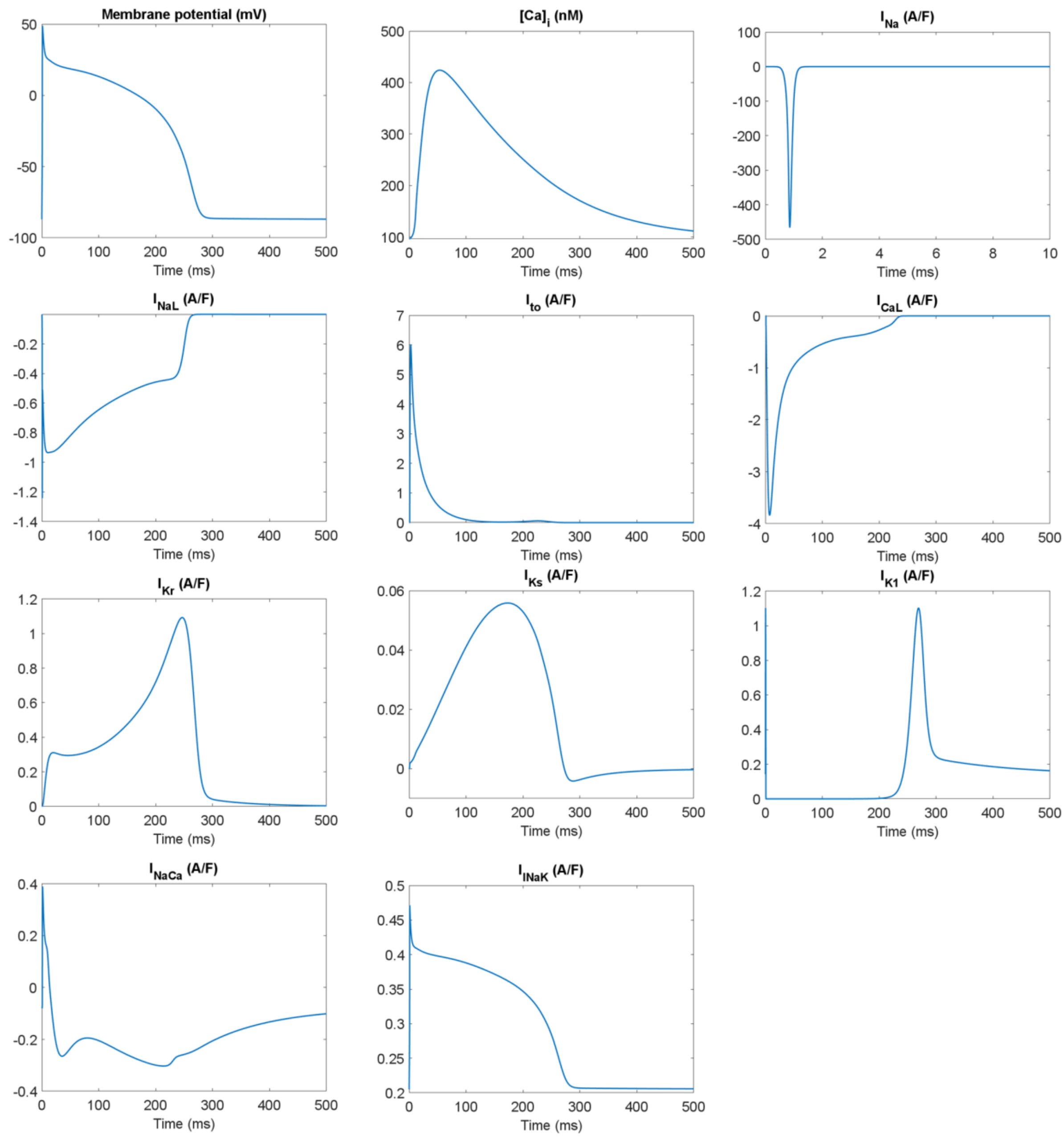

Figure S1. Traces of key ionic currents in T-World. *Please note the different x-axis for* I_{Na} *(top right), used given its extremely rapid activation and inactivation.*

Sodium current (I_{Na}, I_{NaL})

The fast sodium current I_{Na} was implemented based on ToR-ORd, extended with PKA-dependent phosphorylation, similar to Doste et. al. [125]. This yields four distinct populations of channels: unphosphorylated, PKA-phosphorylated, CaMKII-phosphorylated, and dual-phosphorylated. Compared to Doste et al., full PKA activation in our model induces 25% increase in I_{Na} conductance, as used previously by Heijman et al. [40], and a small shift (5 mV) of the voltage-dependent I_{Na} activation curve, similar to canine experimental data [126].

The late sodium current is based on ToR-ORd [4], with τ_{hL} reduced from 200 ms to 145 ms. This yields an I_{NaL} profile that decreases throughout the AP, as observed in human myocytes (this differs from guinea pigs, where I_{NaL} increases throughout most of the AP) [127]. Epicardial cells have 70% I_{NaL} of the endocardium.

L-type calcium current (I_{CaL})

The I_{CaL} model is originally inspired by the formulation in ToR-ORd [4]. Beyond numerous parametric changes to its predecessor, it also includes an additional calcium-dependent inactivation gate $f_{Ca,CDI}$. This helps the model achieve a leaner I_{CaL} profile, while maintaining the capability of manifesting EADs under experiment-like conditions. The leaner profile is advantageous in that much less I_{Kb} is required to achieve a data-like AP shape, compared to ToR-ORd.

As in ToR-ORd, 80% I_{CaL} is in the dyad, with 20% in the subsarcolemmal compartment.

β-AR stimulation has been incorporated following the approach of Doste et al.[125] in the sense that four distinct populations of I_{CaL} are simulated: unphosphorylated, CaMKII-phosphorylated, PKA-phosphorylated, and dual-phosphorylated. However, PKA phosphorylation increases I_{CaL} conductance 1.9-fold, which is in line with the substantial increase observed experimentally [128,129]. In addition, PKA phosphorylation shifts I_{CaL} activation and inactivation leftward by 9 and 6 mV respectively, similar to experimental data [128,129].

Transmural differences in I_{CaL} density were reduced compared to ToR-ORd[4], given experimental data in dog showing little difference in functional I_{CaL} measurements across the wall [130]. Now, epicardium includes a 2.5% increase over endocardium (rather than the 20% in ToR-ORd), and midmyocardium includes a 10% increase over endocardium (rather than 100% in ToR-ORd). The midmyocardial versions of the original ORd and ToR-ORd models show EADs possibly too eagerly due to the strongly enhanced I_{CaL}, which is no longer the case in T-World.

Changes in equations compared to ToR-ORd (and/or the Doste et al. formulation of PKA effects on I_{CaL}) are given below:

$$d_\infty = \left(1.0763 \cdot e^{-1.007 \cdot e^{-0.0829 \cdot V + 3.62483}} \text{for } V \le 31.4978 \,\middle|\, 1 \text{ otherwise}\right)$$

$$\tau_d = 1.5 + \frac{1}{e^{-0.05\cdot(V+6)} + e^{0.09\cdot(V+14)}}$$

$$\tau_{f,fast} = 6.171108886816713 + \frac{1}{0.00126 \cdot e^{\frac{-(V+26.63596)}{9.69961}} + 0.00126 \cdot e^{\frac{(V+26.63596)}{9.69961}}}$$

$$\tau_{f,slow} = 2719.22489 + \frac{1}{7.19411E\text{-}5 \cdot e^{\frac{-(V+5.74631)}{10.8769}} + 7.19411E\text{-}5 \cdot e^{\frac{-(V+5.74631)}{16.31535}}}$$

$$A_{f,fast} = 0.52477$$

$$\tau_{f,Ca,fast} = 13.50673 + \frac{1}{0.1542 \cdot e^{\frac{-(V-1.31611)}{11.3396}} + 0.1542 \cdot e^{\frac{(V-1.31611)}{11.3396}}}$$

$$\tau_{f,Ca,slow} = 177.95813 + \frac{1}{4.73955E\text{-}4 \cdot e^{\frac{-(V+0.79049)}{0.81777}} + 4.73955E\text{-}4 \cdot e^{\frac{-(V+2.4074)}{1.90812}}}$$

$$A_{f,Ca,fast} = 0.3 + \frac{0.6}{1 + e^{\frac{V-9.2424}{27.96201}}}$$

$$\tau_{jca} = 66$$

$$jca_\infty = \frac{1}{1 + e^{-\frac{V+17.66945}{3.21501}}}$$

$$K_{m,n} = 0.00222$$

$$K_{+2,n} = 957.85903$$

$$K_{-2,n} = jca \cdot 0.84191$$

$$\alpha_{n,dyad} = \frac{1}{\frac{K_{+2,n}}{K_{-2,n}} + \left(\frac{K_{m,n}}{[Ca^{2+}]_{dyad}}\right)^{3.80763}}$$

$$\alpha_{n,sl} = \frac{1}{\frac{K_{+2,n}}{K_{-2,n}} + \left(\frac{K_{m,n}}{[Ca^{2+}]_{sl}}\right)^{3.80763}}$$

(here, ‘,dyad’ indicates dyadic variables, and ‘,sl’ indicates subsarcolemmal variables)

Steady-state value, tau, and derivative of the new calcium-dependent inactivation (CDI) gate $f_{Ca,CDI}$ (which is combined with the activation gate by multiplication) for compartment X (dyad or subsarcolemmal) are as follows:

$$\mathrm{f}_{\infty \mathrm{Ca,CDI,X}} = 1 - \frac{1}{1 + \left(\frac{1.86532 \cdot [Ca^{2+}]_X}{0.032}\right)}$$

$$\tau_{f,Ca,CDI,X} = 1.0967 + (1 - \mathrm{f}_{\infty \mathrm{Ca,CDI,X}}) \cdot 141.4299$$

$$r_{recovery} = 0.02313$$

$$\frac{d\mathrm{f}_{\mathrm{Ca,CDI,X}}}{dt} = -\mathrm{f}_{\mathrm{Ca,CDI,X}} \cdot \frac{\mathrm{f}_{\infty \mathrm{Ca,CDI,X}}}{\tau_{f,Ca,CDI,X}} + (1 - \mathrm{f}_{\mathrm{Ca,CDI,X}}) \cdot r_{recovery}$$

The variable $r_{recovery}$ determines rate of recovery from inactivation.

We have updated the driving force calculation based on our observation that the way driving force is typically calculated is problematic for ionic currents located in very small compartments such as dyads. Specifically, we believe it is problematic to use the gradient of extracellular space versus local, dyadic calcium concentrations. Both equations commonly used to describe the driving force (based on the difference from the Nernst potential, V-E_{ion}, or the Goldman-Hodgkin-Katz flux equation) are based on the assumption of well-mixed solutions on either side of the membrane. While this holds approximately for sodium, potassium, or chloride ions, the assumption clearly does not hold for calcium, the concentration of which changes by orders of magnitude in the dyad throughout the excitation-contraction coupling cycle. During peak release of calcium from the SR, local concentrations can reach approx. 100 μM, while bulk cytosol calcium concentrations will be in the order of low hundreds of nM at that time point. The actual physical driving force for dyadic channels at such a time point depends on the intracellular gradient of calcium (high average concentration in the dyad, decreasing with distance from the dyad). In the cell model, this gradient is discretized into two concentrations: dyadic and non-dyadic, using only the dyadic for the driving force. The exact nature of such a discretization becomes surprisingly important. If we use a small dyad model this leads to a very high local calcium concentration in the dyad. Conversely, if we work with a somewhat larger dyad, the average dyadic concentration will be lower. As a result, by changing the size of the modelled dyad, we are changing the average dyadic concentration markedly, and, by extension, we change the

driving force markedly. At the cellular level, a small dyad with high local concentrations can then produce unphysiological I_{CaL} reversal (corresponding to calcium efflux). However, the driving force should principally be determined by the true gradient, not a semi-arbitrary modelling choice. Based on discussions with experts in ionic fluxes across membranes (Prof. Dirk Gillespie from Rush University, Prof. Dezso Boda from University of Pannonia, Prof. Pavel Jungwirth from IOCB in Prague, and Dr. Geir Halnes from University of Oslo), we concluded that the driving force formulation for not-well-mixed solutions should also incorporate non-dyadic calcium concentrations.

Since the exact solution to this issue is currently unknown, different model families take different approaches to this apparent problem. The ToR-ORd model and its predecessors use a very large dyadic space (2% of the cell, like the whole subsarcolemmal space in Shannon-like models). This makes local concentrations in the dyad relatively low, and after the improvements in driving force formulation introduced in ToR-ORd, this leads to a well-shaped I_{CaL}, which does not tend to manifest current reversal. However, the 2% volume is far higher than best available estimates based on imaging data used in the Shannon et al. model and its successors, which is 0.0539%. One of the consequences is that it is extremely challenging to introduce genuinely calcium-sensitive calcium release in the ToR-ORd family, as the calcium influx through I_{CaL} is a mere trickle in the vast dyad. Having RyR respond to the resulting minor calcium elevation produces a hypersensitive phenotype which is then prone to spontaneous release. On the other hand, the most up-to-date model from the Shannon-like family, Morotti2021 [11], made the I_{CaL} driving force insensitive to dyadic calcium, using a constant in its place. This avoids the problem of current reversal, but makes the model insensitive to genuine changes inside the cell. The TP06 model [12] then employs much lower ionic activity inside the cell than outside (which does not seem to be theory-supported) and a shift of 15 mV in the membrane potential used in the driving force calculation (we could not find the origin of this shift, which could be a phenomenological way of avoiding driving force problems and current reversal).

In the absence of a clear answer on how the local and non-local calcium concentrations should be weighted to produce the driving force, we use a “one-compartment-further” approach. Thus, subsarcolemmal calcium concentration is used when calculating the driving force of dyadic I_{CaL}, and myoplasmic calcium concentration is used when calculating subsarcolemmal I_{CaL}. This non-local approach to driving force retains the calcium-sensitive nature of the driving force, but at the same time avoids problems arising from a fully local approach, which would lead to current reversal with high elevations of dyadic calcium. It produces a good data-like shape of I_{CaL} traces, and we are not aware of any major weakness of this way of modelling driving force.

We note, that while the driving force is and should be partly non-local, processes like CDI that are local still use local concentration – the non-local “one-compartment-further” approach is used only when calculating the driving force.

In addition, ionic activity coefficients γ used in the driving force calculation now correctly use base of power 10, rather than e, such as the following for ionic strength I, ionic species X and dyadic compartment:

$$\gamma_{X,dyad} = 10^{-A\cdot z_X^2\cdot\left(\frac{\sqrt{I}}{1+\sqrt{I}}-0.3\cdot I\right)}$$

Transient outward current (I_{to})

Baseline I_{to} was formulated as in the Grandi 2010 model [10]. The conductances of fast and slow component of I_{to} ($I_{to,f}$ and $I_{to,s}$) for different cell types are given in **Table S4.**

Table S4. Conductance of I_{to} components across the ventricular wall.

Cell type	$G_{to,f}$ (mS/μF)	$G_{to,s}$
Endocardial	0.01276	0.0721
Midmyocardial	0.14928	0.04632
Epicardial	0.29856	0.02036

We have additionally incorporated the effect of CaMKII on inactivation of I_{to}, creating separate populations of phosphorylated and non-phosphorylated channels. CaMKII-phosphorylated channels have their activation shifted by 10 mV in the depolarizing direction:

$$x_{to,\infty} = \frac{1}{1 + e^{\frac{-(V-19-10)}{13}}}$$

To represent modulation of I_{to} inactivation by CaMKII, we used an identical approach to that of O'Hara et al. [18], which involves multiplying time constants of inactivation of $I_{to,s}$ and $I_{to,f}$ by the product of $\delta_{CaMK,develop}$ and $\delta_{CaMK,recover}$, defined as follows:

$$\delta_{CaMK,develop} = 1.354 + \frac{10^{-4}}{e^{\frac{V-167.4}{15.89}} + e^{\frac{-(V-12.23)}{0.2154}}}$$

$$\delta_{CaMK,recover} = 1 - \frac{0.5}{1 + e^{\frac{V+70}{20}}}$$

Rapid delayed rectifier current (I_{Kr})

I_{Kr} is based on the ToR-ORd formulation, with the β_i parameter reduced by 30% to improve restitution properties. Similar to the best available estimate of transmural changes [18], G_{Kr} is scaled by 1.25 in epicardium vs endocardium, and by 0.7 in midmyocardium vs endocardium.

Slow delayed rectifier current (I_{Ks})

Similarly to the Morotti2021 model, we have utilized the framework of calcium-sensitive formulation of I_{Ks} by Bartos et al. [131] as a starting point, with the following changes compared to the Morotti2021 implementation. First, the default multiplier of the current (the variable 'gks_factor_SA' in the code) changed from 2.5 to 2.97. Second, the I_{Ks} conductance is reduced down to 50% in midmyocardial cells, given canine data on transmural differences in I_{Ks} density [132]. The equations describing the regulation of I_{Ks} by PKA were adjusted as follows, to enable the model to reproduce the ~14-fold increase in peak I_{Ks} evoked during an AP in βAR stimulated cells compared to controls [133]:

$$G_{Ks,0} = 0.01 \cdot (0.2 + 0.2 \cdot k_{PKA})$$

$$G_{Ks,max} = 0.01 \cdot (0.8 + 7 \cdot k_{PKA}),$$

where k_{PKA} is the effective fraction of phosphorylated I_{Ks}. Subsequently, G_{Ks} for the dyad and subsarcolemmal populations of I_{Ks} are defined as follows:

$$G_{Ks,dyad} = G_{Ks,0} + \frac{G_{Ks,max} - G_{Ks,0}}{1 + \left(\frac{150E\text{-}6}{[Ca^{2+}]_{dyad}}\right)^{1.3}}$$

$$G_{Ks,sl} = G_{Ks,0} + \frac{G_{Ks,max} - G_{Ks,0}}{1 + \left(\frac{150E\text{-}6}{[Ca^{2+}]_{sl}}\right)^{1.3}}$$

Inward rectifier current (I_{K1})

We employed the identical I_{K1} formulation as in ToR-ORd, based on the work of Carro et al. [134]. Epicardial cells have 1.1fold conductance of endocardial ones, with midmyocardial cells showing 1.3fold conductance over the endocardium.

Sodium-calcium exchanger (I_{NaCa})

The formulation of the sodium-calcium exchanger (NCX) was reused from the ToR-ORd model with modification to better represent conditions of sodium overload. We noticed that the model correctly shows increased influx and reduced efflux of calcium when intracellular sodium is high in most conditions, except at diastolic potentials and with resting levels of calcium. In that condition, the original model would paradoxically show increased efflux of calcium in the setting of sodium overload, complicating simulations of sodium-overloaded cells (e.g. with heavy I_{NaK} inhibition such as following ouabain exposure). While sodium overload should translate into calcium overload due to more calcium retention via NCX, this was not captured well by the model given the increased calcium efflux during diastole. The following parametric changes were applied to avoid this problem while preserving the original model functionality:

$$kna1 = 11.9712$$

$$kna2 = 2.76$$

$$kna3 = 88.767$$

$$kassym = 19.4258$$

$$wna = 3.2978\text{E}04$$

$$wca = 5.1756\text{E}04$$

$$wnaca = 2.7763\text{E}03$$

$$kcaon = 3.4164\text{E}06$$

$$kcaoff = 3.8532\text{E}03$$

$$qna = 0.6718$$

$$qca = 0.0955$$

In addition, the membrane potential in the equations for *hca* and *hna* was shifted by -8.3117.

The fraction of NCX in the dyad is 31%, which is in line with a high degree of colocalization observed experimentally [135]. Similar to I_{CaL}, non-local calcium concentrations are used when calcium transport is calculated (dyadic NCX uses subsarcolemmal calcium concentration and subsarcolemmal NCX uses intracellular one), with the exception of allosteric regulation, which is fully local, and uses concentrations in the same compartment.

Epicardial cells have 1.1fold NCX of endocardium, and midmyocardial cells have 1.4fold NCX over endocardium (reflected by differences in G_{NaCa}).

Sodium-potassium pump (I_{NaK})

We noticed that the initially considered I_{NaK} formulation from the ToR-ORd model (which utilized a formulation from the ORd model by O'Hara et al. [18]) is problematic with regards to responses to changes in extracellular potassium. Reduction in extracellular potassium concentration is known to reduce the activity of the sodium-potassium pump [136]. The ToR-ORd/ORd model of I_{NaK} reproduces this phenomenon when unphysiological ionic concentrations (also used in the experiment) are employed (**Figure S2A,B**); however, it does not do so when physiological levels used in actual cell model simulations are used (**Figure S2C**).

As such, we instead used and adjusted a formulation originally based on the Grandi et al. 2010 model [10], which represents the reduction in the pump activity with reduced extracellular potassium in a robust way. We changed the equation for f_{NaK} to:

$$f_{NaK} = 0.75 + \left(0.00375 - 0.001 \cdot \frac{140 - Na_o}{50}\right) \cdot V$$

With this formulation, the voltage-dependence of I_{NaK} is linear, compared to sublinear in Grandi et al. [10], based on the observation that the sublinear relationship is obtained with 50 mM intracellular sodium, whereas a physiological concentration of 8 mM yields a relatively linear relationship (**Figure S2D**). At the same time, the updated model recapitulates the observation that extracellular sodium alters the slope of the voltage-dependence of I_{NaK} (**Figure S2E,F**).

I_{NaK} activation via phospholemman phosphorylation by β-AR system is represented by the inclusion of a PKA-activated I_{NaK} population which has increased affinity for intracellular sodium, similar to experimental measurements [137] (Km_{Nai} = 11 for normal channels in the model, $Km_{Nai,PKA}$ = 8.4615 mM).

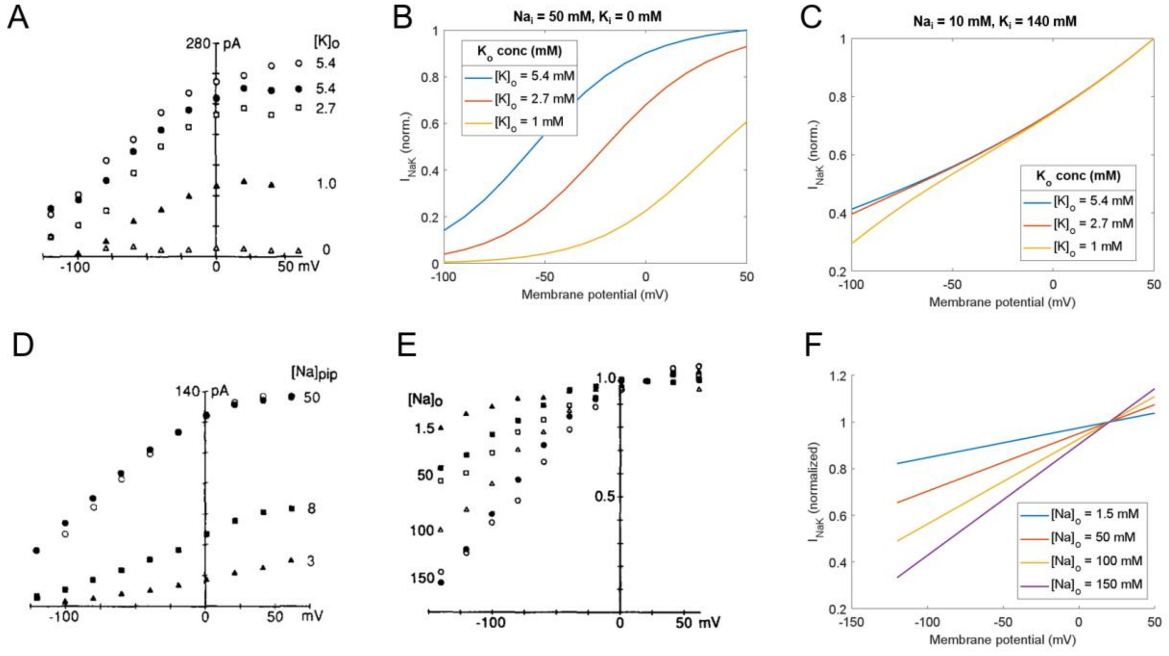

***Figure S2 Sodium-potassium pump properties**. **A)** Dependence of I_{NaK} on extracellular potassium, figure reproduced from Nakao & Gadsby 1989 [138], as permitted by the CC-BY-NC-SA license. **B)** Simulated I_{NaK} using the ToR-ORd/ORd formulation, using high intracellular sodium and zero intracellular potassium, as used in the patch pipette in panel A. **C)** Demonstration of loss of potassium-dependence of I_{NaK} when physiological intracellular concentrations (10 mM sodium and 140 mM potassium) are used in the same model. **D)** Demonstration of sublinear dependence of I_{NaK} on membrane potential at unphysiologically high intracellular sodium, but near-linear dependence at physiological levels. Figure reproduced from the work of Nakao & Gadsby 1989. **E)** Voltage-dependence of I_{NaK} for varying levels of extracellular sodium, again reproduced from Nakao & Gadsby 1989. **F)** Simulation of the new I_{NaK} model, corresponding to data in panel E.*

Chloride currents ($I_{(Ca)Cl}$, I_{Clb})

The calcium-sensitive chloride current $I_{(Ca)Cl}$ and the background chloride current I_{Clb} were formulated as in the Grandi 2010 model [10], with an update to the conductance.

Background currents (I_{Nab}, I_{Cab}, I_{Kb})

Background sodium and calcium currents (I_{Nab}, I_{Cab}) were formulated as in the Grandi 2010 model [10] and the background potassium current (I_{Kb}) as in ToR-ORd, all with updated conductances (**Table S3**). No transmural gradient of those channels is present.

Stimulus current (I_{stim})

A conservative potassium stimulus is used as in ToR-ORd [4], as suggested by Hund et al. [102].

Calcium release from the SR (J_{rel}, J_{leak})

The representation of calcium release from the SR (J_{rel}) combines the approaches from ToR-ORd and Shannon-like models of calcium-induced calcium release [4,9]. Shannon-like models are mechanistically more realistic, with J_{rel} being triggered by dyadic calcium, rather than I_{CaL} (which is the case in ToR-ORd), but they suffer from the issue of late-peaking release (see **Figure S9)**. The mechanistic importance is not just for its own sake, but also for enabling the formation of delayed afterdepolarizations (DADs), which do not occur spontaneously in ToR-ORd. We therefore designed a hybrid scheme, where a very small population of RyR (approx. 4%) is directly coupled to I_{CaL} (based on a modified ToR-ORd formulation), representing the most tightly coupled I_{CaL}-RyR clusters. This provides an early source of calcium influx, which then helps activate at the right time the much larger population of RyR that are modelled using a modified Shannon-like formulation. The latter has been modified predominantly to support the earlier time to peak of calcium release, and to enable calcium-driven alternans. In addition, passive leak from the SR to the dyad is included in the model. The equations for the distinct components are given below.

I_{CaL}-activated RyR flux ($J_{rel,ICaLdep}$)

The formulation is a reparametrized and updated version of J_{rel} from ToR-ORd with a small maximum flux and two additional inactivation gates to keep its duration short and thereby keeping the overall amount of calcium released in this way low. The $J_{rel,ICaLdep}$ flux over time is shown in **Figure S3A**, demonstrating a minor role in the overall release. We furthermore confirmed that calcium-driven alternans manifests in T-World even when the $J_{rel,ICaLdep}$ component of release is turned off (**Figure S3B**). I.e., alternans arises from the "main" calcium-sensitive component of calcium release and is not driven by the I_{CaL}-sensitive component.

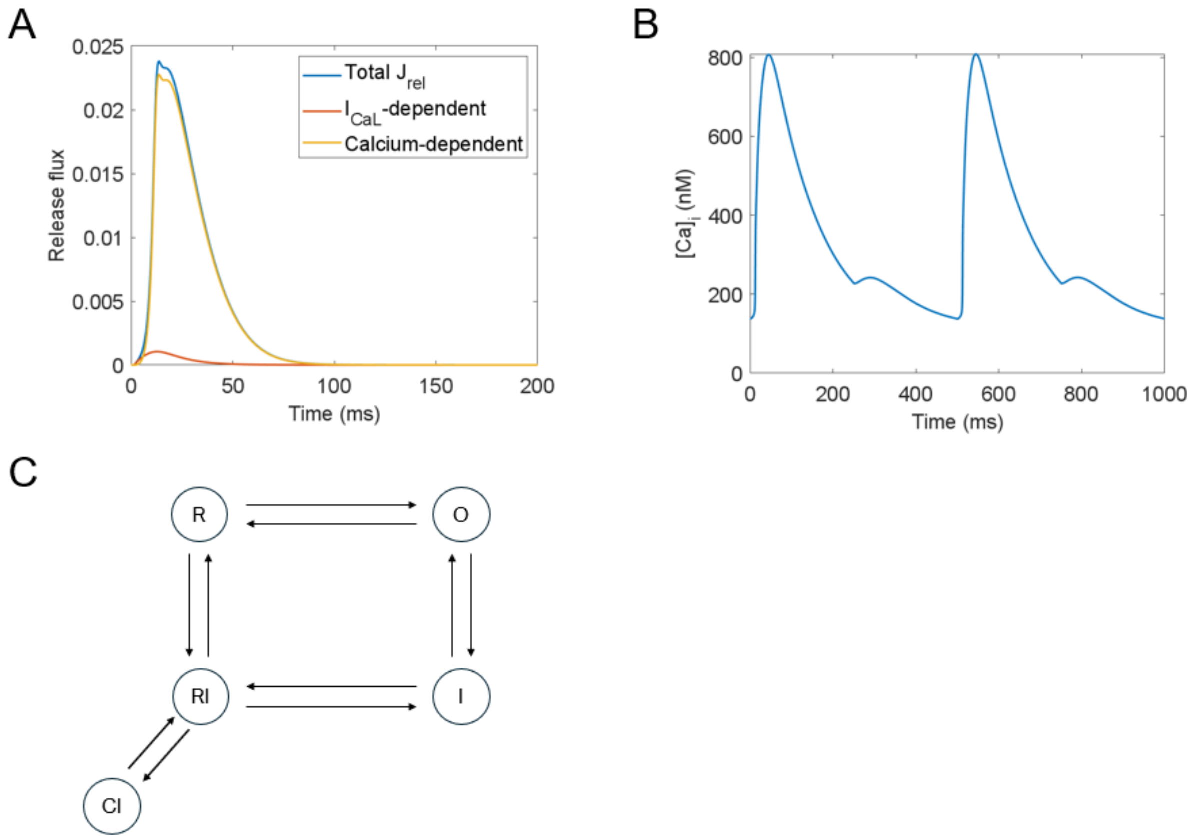

Figure S3. Modelling ryanodine receptors. A) *Flux through RyR: total and its two sub components (I_{CaL}-dependent and calcium-dependent), demonstrating that the calcium release from the SR is largely calcium-sensitive (the I_{CaL}-dependent component is only 4.2% of total release with regards to peak and 3.8% with regards to integral).* ***B)*** *Illustration of presence of calcium-driven alternans at basic cycle length of 260 ms even when the I_{CaL}-dependent component of release is turned off.* ***C)*** *A diagram of the calcium-sensitive RyR Markov model.*

The derivative of the activation gate of the directly coupled RyR ($J_{rel,ICaLdep,act}$) is given by:

$$I_{CaL,junc,sigmoided} = 1 - \frac{1}{1 + \left(\frac{|I_{CaL,dyadic}|}{0.45}\right)^{4.5}}$$

$$J_{rel,ICaLdep,act,\infty} = 15.5959 \cdot \frac{I_{CaL,junc,sigmoided}}{1 + \left(\frac{0.95271}{[Ca]_{SR}}\right)^{7.72672}}$$

$$\tau_{rel} = \max\left(\frac{12.4767}{1 + \frac{0.0123}{[Ca]_{SR}}}, 0.001\right)$$

$$\frac{dJ_{rel,ICaLdep,act}}{dt} = \frac{J_{rel,ICaLdep,act,\infty} - J_{rel,ICaLdep,act}}{\tau_{rel}}$$

The derivatives for the two inactivation gates ($J_{rel,ICaLdep,f1}$, $J_{rel,ICaLdep,f2}$) are calculated as follows:

$$\frac{dJ_{rel,ICaLdep,f1}}{dt} = \frac{\frac{1}{1 + \frac{I_{Ca,junc,sigmoided}}{0.001}} - J_{rel,ICaLdep,f1}}{64.11202}$$

$$\frac{dJ_{rel,ICaLdep,f2}}{dt} = \frac{\frac{1}{1 + \frac{I_{Ca,junc,sigmoided}}{0.0006}} - J_{rel,ICaLdep,f2}}{119.48978}$$

The total I_{CaL}-sensitive RyR flux is calculated as follows:

$$J_{rel,ICaLdep} = 0.00174 \cdot J_{rel,ICaLdep,act} \cdot J_{rel,ICaLdep,f1} \cdot J_{rel,ICaLdep,f2}$$

Calcium-activated RyR flux ($J_{rel,Cadep}$)

The calcium-sensitive component of the SR release of calcium via RyR is based on an extensively reparametrized 4-state square Markov model by Shannon et al. [9]. This was extended with a single "calcium-inactivation" state (**Figure S3C**), providing an additional partial source of RyR refractoriness. In particular, this helps the model to reproduce at rapid pacing the combination of high SR load, but somewhat limited RyR release (indicated by the limited SR depletion e.g. in [30]), less than might be expected from the load-release relationship. The equations determining the transitions between states are as follows:

R → O, RI → I:

$$k_{Ca,SR} = 15 - \frac{14}{1 + \left(\frac{0.75385}{[Ca]_{SR}}\right)^{5.09473}}$$

$$k_{o,SRCa} = \frac{23.87221}{k_{Ca,SR}}$$

$$transition_{R \to O \& RI \to I} = 0.52967 \cdot k_{o,SRCa} \cdot \left([Ca]_{dyad}\right)^{2.06273}$$

O → R, I → RI:

$$transition_{O \to R \& I \to RI} = 0.16219$$

O→I, R→RI:

$$k_{i,SRCa} = 0.39871 \cdot k_{Ca,SR}$$

$$transition_{O \to I \& R \to RI} = 0.94428 \cdot k_{i,SRCa} \cdot \left([Ca]_{dyad}\right)^{0.68655}$$

I → O, RI → R:

$$transition_{I \to O \& RI \to R} = 0.04311$$

RI → CI:

$$transition_{RI \to CI} = 3.0232E\text{-}4 \cdot \left(0.93249 + \frac{29.2005}{1 + \left(\frac{0.001}{[Ca]_{dyad}}\right)^{5.93447}}\right)$$

CI → RI:

$$transition_{CI \to RI} = 0.00248$$

The overall calcium flux through the calcium-sensitive, non-phosphorylated RyR is as follows:

$$J_{rel,Cadep,NP} = 26.6 \cdot O \cdot ([Ca]_{SR} - [Ca]_{junc}),$$

where O is the fraction of RyRs in the open state.

A separate population is simulated for RyR phosphorylated by CaMKII. The equations are identical, except the transition from R→O (and symmetrically RI→I) is increased by 50% to represent the increased open probability of RyR following CaMKII phosphorylation [139]. Using this, the flux through calcium-sensitive phosphorylated RyR ($J_{rel,Cadep,P}$) is calculated analogously.

Combined J_{rel}

The combined flux is calculated as follows:

$$J_{rel} = (1 - f_{RyRP,CaMKII}) \cdot (J_{rel,Cadep,NP} + J_{rel,ICaLdep}) + f_{RyRP,CaMKII} \cdot (J_{rel,Cadep,P} + J_{rel,ICaLdep})$$

I.e., it is a weighted sum of CaMKII-phosphorylated and unphosphorylated RyR flux, weighted by the fraction of RyR phosphorylated by CaMKII ($f_{RyRP,CaMKII}$).

RyR leak ($J_{SR,leak}$)

The leak between the SR and the dyads is represented similarly to Shannon et al. [9], extended to account for CaMKII-dependent potentiation of SR calcium leak [139] and super-linear load-dependence [38].

$$multiplier_{CaMKII} = 1 + 2 \cdot f_{RyRP,CaMKII}$$

$$multiplier_{nonlinear} = 0.2144 \cdot e^{1.83 \cdot [Ca]_{SR}}$$

$$J_{SRleak} = 1.59306E\text{-}6 \cdot ([Ca]_{SR} - [Ca]_{junc}) \cdot multiplier_{CaMKII} \cdot multiplier_{nonlinear}$$

Calcium reuptake to the SR (J_{up})

The formulation of SERCA pumps was mainly based on the Shannon et al. formulation [9], with the following parametric changes (in addition to $V_{max,SERCA}$, listed in **Table S3**): K_{mr} = 2.31442, K_{mf} = 0.30672E-03, H (Hill coefficient) = 1.02809. The maximum pumping rate $V_{max,SERCA}$ in the epicardium is increased by 20% versus (baseline) endocardium, following the report of an increased epicardial SERCA protein expression by Laurita et al. [140].

We have additionally incorporated SERCA potentiation by phospholamban phosphorylation (possible via the CaMKII and PKA pathways). This is represented by a separate population of SERCA pumps with a greater affinity for cytosolic calcium (half K_{mf} versus unphosphorylated, $K_{mf,phosphorylated}$ = 1.5336E-04). The fraction of phosphorylated SERCA pumps is calculated as the sum of fraction of pumps phosphorylated by CaMKII and those phosphorylated by PKA, minus the product of those fractions.

Compared to ToR-ORd, PLB/SERCA are only marginally phosphorylated by CaMKII, reflecting experimental observations by Huke and Bers, who observed minimal phosphorylation by CaMKII at this site [141]. Nevertheless, major potentiation of SERCA pumping rate was observed experimentally at rapid versus slow pacing [142]. We represent this phenomenon phenomenologically using a separate set of equations similar to how CaMKII is activated by calcium, which are however distinct from CaMKII phosphorylation itself. Therefore, when CaMKII overexpression or hyperactivity are simulated in future studies, this calcium-based activation will be unaffected. We note that in normal conditions, this calcium-based activation is functionally similar to ToR-ORd, where SERCA/PLB can be relatively highly phosphorylated by CaMKII. The degree of activation of this mechanism is determined as follows (reusing some of the CaMKII-related named constants, but not state variables):

$$\alpha_{SERCA} = 0.05$$

$$bound_{SERCA} = CaMK_0 \cdot \frac{1 - SERCA_{casig,trap}}{1 + \frac{Km_{CaMK,Ca}}{[Ca]_{dyad}}}$$

$$SERCA_{casig,act} = bound_{SERCA} \cdot SERCA_{casig,trap}$$

$$\frac{dSERCA_{casig,trap}}{dt} = \alpha_{SERCA} \cdot bound_{SERCA} \cdot SERCA_{casig,act} - \beta \cdot SERCA_{casig,trap} \cdot (0.1 + 0.9) \cdot \frac{PP1_{tot}}{0.1371}$$

The $SERCA_{casig,act}$ variable is subsequently used in the equation describing the multiplier applied to $V_{max,SERCA}$:

$$V_{max,multiplier} = 1 + \frac{1.111423947401174}{1 + \left(\frac{0.4}{SERCA_{casig,act}}\right)^2}$$

A similar principle can likely be used in the future to rescue the relatively negative frequency-force relationship in models utilizing the Soltis-Saucerman CaMKII model[143], accelerating calcium reuptake at rapid pacing. However, this will also require a cell model reproducing the positive relationship between SERCA activity and calcium transient amplitude (as T-World does, see **Figure 2C** of the main manuscript).

Sarcolemmal calcium pump (p_{Ca})

The sarcolemmal calcium ATPase was formulated as in the Grandi 2010 model [10], with an update to the conductance.

Buffering and ion diffusion between compartments

Cell compartmentalization and buffering are based on the Shannon et al. framework [9]. One exception is the buffering of calcium by Troponin C, which is replaced by the Land contraction model formulation (see next section). The following parametric changes were made:

$$Bmax_{SR} = 17.85854E\text{-}3$$

$$Bmax_{SLlow,sl} = 33.923E\text{-}3 \cdot \frac{V_{myo}}{V_{sl}}$$

$$Bmax_{SLlow,dyad} = 4.89983E\text{-}4 \cdot \frac{V_{myo}}{V_{sl}}$$

$$Bmax_{SLhigh,sl} = 12.15423E\text{-}3 \cdot \frac{V_{myo}}{V_{sl}}$$

$$Bmax_{SLhigh,dyad} = 1.75755E\text{-}4 \cdot \frac{V_{myo}}{V_{sl}}$$

$$Bmax_{CSQN} = 136.55214E\text{-}3 \cdot \frac{V_{myo}}{V_{SR}}$$

V_{myo}, V_{sl}, V_{junc} and V_{SR} indicate the volumes of myoplasmic, subsarcolemmal, dyadic (junctional), and SR compartments.

Diffusion coefficients of calcium between compartments are:

$$J_{Ca,juncsl} = \frac{1}{3.06685E12}$$

$$J_{Ca,slmyo} = \frac{1}{0.74556E11}$$

Contraction modelling

When including the representation of contraction in T-World, we were initially faced with the choice of whether to take as a starting point the model by Negroni et al. [144] or the Land et al. [21], the two currently leading published formulations.

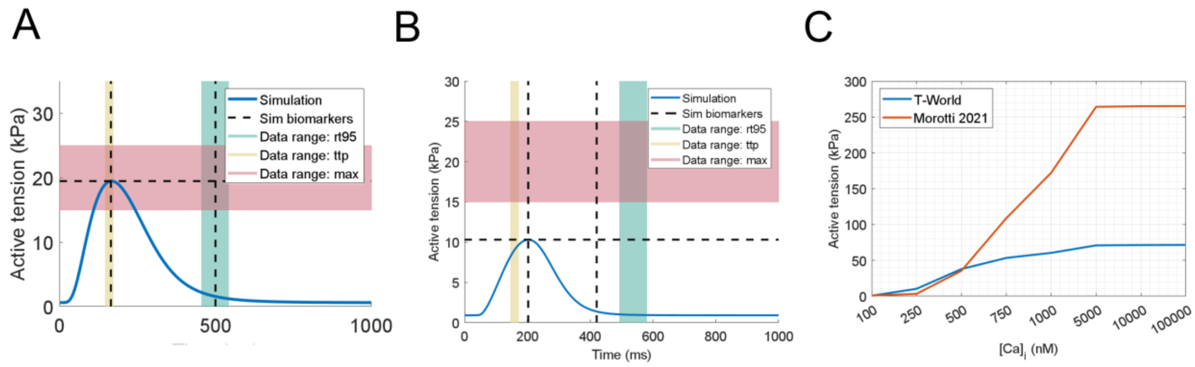

Figure S4. Contraction properties of Land and Negroni-based models. A) *T-World contraction with biomarker ranges shown as in Figure 1 of the main manuscript.* ***B)*** *Corresponding simulation of contraction in the Morotti2021 model (please note that the model was not recalibrated for the experimental ranges like T-World was, i.e., the lower agreement does not necessarily imply an overall worse model).* ***C)*** *Comparison of steady-state active tension between T-World and Morotti2021 models evoked by a range of calcium concentrations. The end points of the curves reflect peak force achievable by the model.*

In principle, starting with the Negroni model would be simpler, given that it is already integrated in the calcium handling framework that T-World is based on (and it provides contraction representation e.g. in the Morotti2021 model [11], referred to extensively in this project). Both Land and Negroni models are capable of generally data-like force development when integrated in cardiomyocyte models (**Figure S4A,B**). The key factor in our decision was the correspondence between the models and the observation that the twitch force during normal activation by an action potential reaches ca. 25-70% of peak contraction force possible [145,146]. While the T-World model with the Land model is in agreement with this (ca 28% twitch force versus peak contraction shown in **Figure S4A** vs **Figure S4C**), this does not hold for the Negroni model embedded in Morotti2021, which reaches only around 4% of peak force during a normal twitch (**Figure S4B** vs **S4C**). This appears to be a result of combination of slow calcium binding to the contractile apparatus, and then additionally slow force development following calcium binding. Given the extensive reparameterization that would be needed, we decided to adopt the Land model as a baseline, with the following parametric changes:

$$TRPN_n = 1.65 \text{ (based on [147])}$$

$$Ktm_{unblock} = 0.02626$$

$$k_{off} = 0.07854$$

$$ca_{50} = 0.7645$$

$$nu = 10.15996$$

$$mu = 3.94046$$

$$T_{ref} = 80$$

The contraction model was coupled to the cellular model via the Troponin C calcium buffering, where the original formulation was replaced by the buffering carried out by the Land model.

We have additionally incorporated the effect of βAR stimulation on the contractile apparatus. Similarly to Negroni et al. [144], we separated the effects into two sites: Troponin I and Myosin binding protein C.

Troponin I phosphorylation is known to reduce the affinity of the contractile apparatus to calcium [148,149]. This is represented in T-World by scaling the ca_{50} parameter (midpoint of calcium-force relationship curve) by the following expression:

$$ca50_{scaling} = 1.45 - \frac{0.45 \cdot f_{TnI,PKA}}{1-0.0031},$$

where $f_{TnI,PKA}$ is the effective fraction of Troponin I phosphorylated by PKA.

Similar to Negroni et al. the acceleration of contraction kinetics was assigned to the Myosin binding protein C phosphorylation site, with the scaling factor (applied to the k_{ws} and xb_{uw} parameter) being as follows:

$$PKA_{XB,acceleration} = 1 + 0.5 \cdot f_{MyBPC,PKA},$$

where $f_{MyBPC,PKA}$ is the effective fraction of Myosin binding protein C phosphorylated by PKA.

In addition, peak force T_{ref} is scaled by $1+0.26 \cdot f_{MyBPC,PKA}$, reflecting the ca. 20-30% increase in maximal force observed following PKA activation [150].

During the development, we noticed a possible issue in the coupling of the Land model to our prior model ToR-ORd, linked to the buffering of calcium by the Land model being slower than the baseline buffering by Troponin C. As a result, the released calcium stays longer in the cytosol before being buffered, and in the setting of enhanced calcium release (e.g. in midmyocardial cells or when I_{CaL} is otherwise increased), a non-data-like two-peak CaT may emerge (see e.g. Fig 4A, midmyocardial cell, in [20]). However, this issue is not present in T-World, which retains normal-looking Ca transient even in the setting of strongly enhanced calcium release (e.g. with β-AR stimulation, see **Figure 3** of the main manuscript). This results from the inclusion of sarcoplasmic reticulum buffering sites in the myoplasmic compartment in T-World (similarly to Shannon-based models, but unlike ToR-ORd and its predecessors). These sites provide sufficiently strong buffering capacity to prevent the aberrant CaT morphology, even when the Troponin C buffering is switched from the original to Land-based formulation.

CaMKII signaling

We decided to use a relatively simple formulation of CaMKII activation by calcium, similar to ToR-ORd [4]. CaMKII targets in T-World comprise I_{Na}, I_{NaL}, I_{to}, I_{CaL}, RyR, and SERCA pumps (via PLB). We also considered using the more complex model by Soltis-Saucerman [143], but ultimately decided against it, given a) a much higher complexity increasing simulation time considerably, b) a problematic effect of CaMKII activation on cellular physiology. Specifically, we observed that CaMKII activation at rapid pacing leads to reduction in SR calcium content and mostly negative rate-dependence of calcium transient (as seen also in Supplementary note 3, Morotti2021 model, which utilizes the Soltis-Saucerman model).

We nevertheless took inspiration from the Soltis-Saucerman model, reflecting the prediction that different sites in the cell are likely phosphorylated to different extent at different heart rates (unlike ToR-ORd or ORd CaMKII representation, which phosphorylates all targets equally). This is achieved by using the following equations when calculating phosphorylation levels:

$$CaMK_{\infty phos,CaMKII,ICaL} = \frac{CaMKII_{active}}{CaMKII_{active} + 0.35}$$

$$CaMK_{\infty phos,CaMKII,RyR} = \frac{CaMKII_{active}}{CaMKII_{active} + 1}$$

$$CaMK_{\infty phos,CaMKII,PLB} = \frac{CaMKII_{active}}{CaMKII_{active} + 10}$$

$$\frac{df_{CaMKII,ICaL}}{dt} = \frac{CaMK_{\infty phos,CaMKII,ICaL} - f_{CaMKII,ICaL}}{10000}$$

$$\frac{df_{CaMKII,RyR}}{dt} = \frac{CaMK_{\infty phos,CaMKII,RyR} - f_{CaMKII,RyR}}{10000}$$

$$\frac{df_{CaMKII,PLB}}{dt} = \frac{CaMK_{\infty phos,CaMKII,PLB} - f_{CaMKII,PLB}}{100000},$$

where $CaMKII_{active}$ is the fraction of active CaMKII, $f_{CaMKII,X}$ is the fraction of target X phosphorylated by CaMKII, and ∞ in subscript indicates a steady-state value.

In addition, the value of the $CaMKII_0$ parameter of the CaMKII signaling was set to 0.1, and the $Km_{CaMK,Ca}$ to 0.0075.

The resulting phosphorylation levels at different heart rates are shown in **Figure S5**. I_{Na}, I_{NaL}, and I_{to} use identical phosphorylation levels as the RyR.

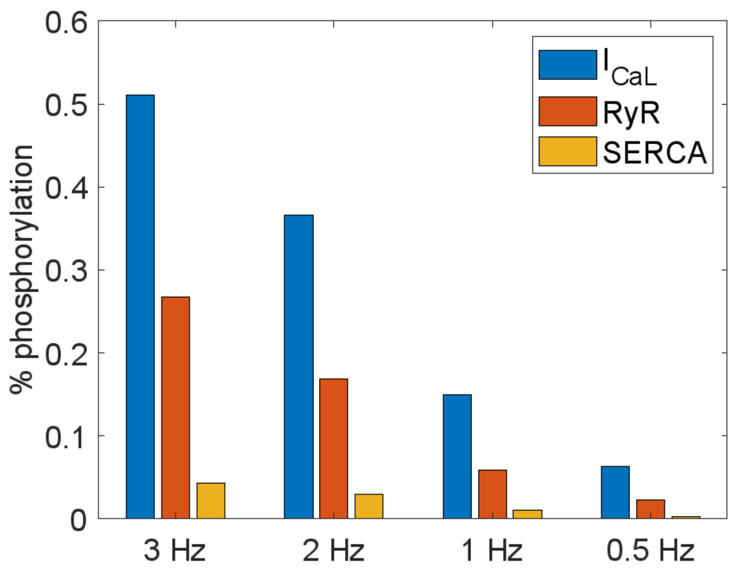

***Figure S5**. **Simulated fractional rate-dependent CaMKII activation at three different subcellular sites.** The phosphorylation for each target was determined as an average value throughout the last action potential out of 250 at a given stimulation rate.*

βAR signaling

The representation of effects of sympathetic nervous activation on cardiomyocytes via (βAR) stimulation consists of two relatively separate parts. The first part consists of a signaling pathway, which calculates the effective phosphorylated fractions of Protein Kinase A (PKA) targets based on the concentration of a beta-agonist. This part is simulated based on the Doste

et al. implementation of the Heijman et al. model [40]. We adjusted cell radius in the signaling model to match that of T-World (10.25 μm which is derived from Shannon/Grandi models, rather than original 11 μm). To retain original model's behavior, we changed the equation 'iks_f_hat_val = (fp_iks - c(167)) / (0.785 - c(167))' to 'iks_f_hat_val = (fp_iks - c(167)) / (0.7 - c(167))'.

The second part of how sympathetic signaling is represented then describes how a given level of phosphorylation translates into changes in target function. We formulated these changes based on available literature, and they are described in corresponding sections above, when changes to ionic currents and fluxes are described. The targets affected by PKA phosphorylation are I_{Na}, I_{CaL}, I_{Ks}, phospholamban (regulating SERCA pumps and thus J_{up}), phospholemman (regulating the NKA pump and thus I_{NaK}), and the contractile apparatus (at two sites, Troponin I and Myosin binding protein C).

The signaling pathway may be turned off, with the user directly supplying the phosphorylated fractions of the PKA targets instead, which enables simulating βAR activation without the increased runtime resulting from simulating the signaling pathway.

Sex differences

The inclusion of sex as a biological variable is becoming an important factor for in-silico investigations due to the historical under-representation of the female sex in basic research [151] and clinical studies [152]. We developed sex-specific variants of the electrophysiological model through variations of the conductance of specific ionic channels which have been identified with significantly different expression in human genomics studies [153] and validate these variant models through comparison with experimentally observed differences in male and female adult electrophysiology. Using a similar approach to Holmes et al.[51], Yang and Clancy [49], Peirlinck [50], we apply scaling factors to ionic channel conductance representative of the published differences in expression of corresponding proteins in un-diseased adult male and female human cells (**Table S5**). The sex-neutral version of T-World, with all scalars set to 1.0, is used throughout this article unless specified otherwise.

To additionally represent the I_{CaL} elevation at the base of female heart[55], we increased the current by 28% to generate the yellow traces in **Figure 4C** of the main manuscript.

Table S5. T-World sex-specific conductance modifications and corresponding evidence. *$CMDN_{Max}$ determines the total amount of calmodulin.*

Conductance	Male Scaling Factor	Female Scaling Factor	Evidence
G_{NaCa}	0.9325	1.0724	Upregulated in female rabbits and rats [154]. (Furthermore upregulated via estrogen in humans [58]).
G_{Kr}	1.1	0.87	I_{Kr} subunits more expressed in males [153].
G_{Ks}	1.09	0.905	I_{Ks} subunits more expressed in males [153].
G_{K1}	1.07	0.92	I_{K1} subunits more expressed in males [153].
G_{pCa}	0.8	1.28	Higher calcium pump expression in females [153].
$CMDN_{Max}$	0.909	1.1	More calmodulin in females [153].

Arrhythmia studies

EADs

In order to evoke EADs, we simulated T-World for 100 beats at 0.25 Hz, with extracellular calcium of 2 mM, and with maximum conductance of I_{Kr} reduced by 85%, based on the work of Guo et al. [54]. The last beat of the 100 was investigated for the presence of EADs.

DADs and stochastic DADs

To evoke a single deterministic DAD leading to triggered activity, we pre-paced T-World for 200 beats at 2.5 Hz with full β-AR stimulation and 3.25 mM extracellular calcium. This was followed by one more paced stimulus followed by several seconds of quiescence, during which the presence of DADs was recorded. To obtain a train of multiple stimuli, we used the same protocol, except faster 3 Hz pre-pacing for 150 beats was used. To sensitize RyR, we increased the R→O (and symmetrically RI→I) transition by 100%.

We also provide a separate implementation of T-World with stochastic DADs resulting from spontaneous overload-driven release, similar to Colman (2019)[65]. This is provided in a separate model code function, given the use of global variables, which we found to be necessary as explained below, but which are otherwise discouraged in Matlab and we avoid using them in the baseline code. The overall idea is simple: provided that calcium in the SR is over a given threshold, there is a certain probability that a spontaneous release may occur, lasting for a parameter-determined duration. Here we used 0.9 mM as the minimum SR content, 100 ms as release duration, and the probability of spontaneous release such that on average 1 DAD occurs per 10 seconds. Cells were pre-paced for 100 beats at 2.5 Hz, using 3 mM extracellular calcium. Once a spontaneous release is determined to occur, starting at *timeStartDAD* and ending at *timeEndDAD*, with amplitude given by the parameter *amp*, the formula for the spontaneous release at time $t \in (timeStartDAD, timeEndDAD)$, which is added to the SL compartment) is:

$$J_{rel,stoch} = amp \cdot 2 \cdot \frac{\min(t - timeStartDAD, timeEndDAD - t)}{timeEndDAD - timeStartDAD}$$

I.e., it is a triangle-shape release, zero at the ends of the time interval, and with maximum at the midpoint between starting and ending time. Using such a spontaneous release function is straightforward when using a fixed-step method to solve ODEs. However, particularly for single cell simulations, it is much more efficient to use variable step solver, such as @ode15s in Matlab. In such a case, one needs to scale the probability of DAD starting at a given time step by the length of the time step (otherwise, if kept uniform, DADs would occur more frequently earlier during the beat's simulation, where time step is smaller). However, such scaling in Matlab is complicated by the fact that there is, to our knowledge, no clear way of finding the current step size. We designed an approximate scheme, which uses global variables to store the information on previous step's starting time, which can be used to calculate the current time step, as the current time is available in the ODE system code. In addition, we have furthermore limited the maximum time step to 25 ms. If left unconstrained, the solver can choose time steps over 100 ms in later phases of a beat simulation, which makes it skip a DAD that was determined to happen, which leads to under-occurrence of late DADs. Finally, we note that the current formulation of stochastic DADs with a fixed probability of spontaneous release when calcium in the SR exceeds a threshold is rather basic – future versions may modulate the probability by the SR content, and possibly also by calcium in the adjacent SL compartment. Similarly, although the triangular shape is qualitatively consistent with progressive recruitment of RyRs during a calcium wave, followed by a gradual decrease as the wave reaches the end of the cell with fewer available calcium release units, it represents a significant simplification of

the different processes involved in calcium-wave propagation. Our approach can be substituted by more advanced formulations, e.g., based on Colman (2019).[65]

The stochastic formulation of DADs is also a practical way of achieving subthreshold DADs as well as triggered activity in the same parameterization of a model (unlike the default T-World, which produces triggered activity). We note the fact that T-World DADs lead to action potential development, whereas they remain mostly subthreshold in the Shannon et al. model[9] is not primarily about differences in calcium handling, but about the amount of I_{K1}. The Shannon model has ca. fourfold I_{K1} and hence it maintains the resting potential much more strongly, usually not permitting the development of DADs into triggered activity. We confirmed that when T-World has I_{K1} increased four times, DADs are likewise converted to subthreshold ones (not shown).

Alternans

To evoke alternans, we simulated T-World for 250 beats at a range of frequencies: basic cycle lengths 240, 250, 260, ... 400, 420, 440,... 500, 600, 700, ..., 1000 ms, recording the last 4 beats. In addition to the control model, a version with 65% SERCA pump V_{max} (0.65 J_{up} multiplier), 150% SERCA V_{max}, and β-AR-stimulated versions were simulated.

Restitution

When measuring the slope of the restitution, we pre-paced T-World for 100 S1 beats, which were followed by a pair of stimuli S2 ms apart. S2 intervals explored ranged from S1 down to 200 ms. The default S1 interval was 1000 ms (1 Hz), with 600 and 400 used for a part of the simulation, as described in the corresponding figure. Action potential duration was measured at the level of -75 mV. Peak slope of the S1S2 restitution curve was determined as the maximum slope of diff(APD_{S2})./diff(diastolic interval), where APDS2 is the vector of APDs of all the S2 stimuli. Data corresponding to S2 stimuli which did not reach the threshold of 0 mV were discarded. To explore the relationship between the baseline cell APD and its peak restitution slope, we created 300 models with randomly perturbed I_{CaL}, I_{Kr}, I_{Ks}, I_{NaL}, I_{NaK}, I_{NaCa} (each parameter scaled by a number between $e^{-0.5}$ and $e^{0.5}$; the exponent was randomly drawn from uniform distribution between -0.5 and 0.5). This was done for T-World, Morotti 2021 and TP06 models (in the case of TP06, I_{NaL} was effectively not changed, as the model does not represent I_{NaL}).

Stability of arrhythmic behaviors

We assessed the stability/robustness of arrhythmic behaviors in a population of models with varied parameters, calibrated to human-derived criteria in **Table S6**. I_{Na}, I_{NaL}, $I_{to,f}$, I_{CaL}, I_{K1}, I_{Kr}, I_{Ks}, J_{rel}, J_{up}, I_{NaCa}, I_{NaK} were varied between 67% and 150% (sampled using log-multipliers, so that x-fold reduction is equally likely as x-fold increase). 786 out of 1000 models generated in this way passed all the calibration criteria.

Table S6. Biomarker ranges for the calibration of population of models used to test stability of arrhythmogenic behaviors. *Based on studies summarized in* [20,155]

Biomarker	Minimum	Maximum
AP peak	7 mV	55 mV
Resting membrane potential	-95 mV	-80 mV
Peak upstroke velocity	100 V/s	1000 V/s
APD90 (Action potential duration at 90% recovery)	180 ms	440 ms
APD50 (APD at 50% recovery)	110 ms	350 ms
APD40 (APD at 40% recovery)	85 ms	320 ms
90-40 triangulation (APD90-APD40)	50 ms	150 ms
CaT duration at 90% recovery	220 ms	750 ms

CaT duration at 50% recovery	120 ms	420 ms
CaT amplitude	200 nM	600 nM
Peak of CaT	200 nM	1000 nM
Diastolic Ca	0 nM	400 nM
Peak active tension	5 kPa	40 kPa
Time to peak active tension	120 ms	200 ms
Time from peak active tension to 95% recovery	200 ms	600 ms

To assess stability of EADs in the 786 chosen models, we varied together I_{CaL} and I_{Kr}, adding those changes (via multiplication) to the parameterization of each model. For each model, we explored I_{CaL} multipliers of 1, 1.01,...,1.45, concurrently with I_{Kr} multipliers of 1, 0.98, ...0.1. In this way, EADs are gradually promoted through a medium-strength I_{CaL} increase and a reduction in repolarization. For each model, we recorded the earliest pair of I_{CaL} and I_{Kr} multipliers producing EADs.

To assess the stability of deterministic DADs, we simulated each of the 786 models with fully active β-AR signaling, at extracellular calcium of 2, 2.2, ..., 6 mM. 200 beats of pre-pacing were used at 2.5 Hz, recording how much extracellular calcium each model requires to trigger DADs in a period of quiescence following the pre-pacing phase. These simulations were done separately in a baseline model and in a model with 50% increase in J_{up}, promoting SR overload.

To assess stability of alternans in T-World, we simulated each model with J_{up} multiplier of 0.5, 0.6, ..., 1.5, at basic cycle length of 260 ms, recording the number of multipliers which yield alternans at the end of 100 beat pacing train.

To assess the stability of S1S2 restitution, we simulated the S1S2 protocol in each of the 786 models, recording the peak restitution slope (100 S1 beats followed by a S2 beat after 200,205,...,1000 ms).

The same ranges and criteria (not including contractility) were used for other models in simulations underlying **Figure 7** and **Figure S27**. In the case of TP06, alternans was simulated at basic cycle length (bcl) of 320, given the model's relatively long APD and thus refractory period. For Morotti2021, the model was simulated at bcl of 280 ms, given its longer APD than T-World.

Graphical user interface

We provide a graphical user interface (GUI) for simulating T-World for a choice of parameter values and a variety of stimulation protocols. Users can visualize different variables and download simulation data for further analysis. The GUI supports four stimulation protocols: (i) regular pacing, (ii) S1-S2 pacing, (iii) pacing over a range of intervals (suitable, e.g., for alternans), and (iv) regular pacing followed by a pause (suitable, e.g., for DADs). For protocol (ii), the GUI generates a restitution curve, while for protocol (iii), it displays a bifurcation diagram of the APD or another variable as a function of the pacing interval. The GUI is implemented in Plotly Dash and uses Myokit [156] for model simulation. It is accessible online or available for download. The simulator and further details are available at https://t-world-simulator-multipage-production.up.railway.app/.

Parameter optimization using genetic algorithms

Throughout the development of T-World, we frequently used multiobjective genetic algorithm (MGA, @gamultiobj in Matlab) to fit parameters of single model components, as well as for integrating components together. In general, a range of protocols would be simulated with a candidate model, with the simulation outputs compared to reference values based on

experimental data. Below we share several insights we have obtained throughout the process, which could be valuable to other users of the method.

- **Number of fitness dimensions.** Each simulated protocol produces a single error describing the deviation of simulation from experiments. We would often work with 10-20 different errors that are to be optimized. Putting them in a single fitness criterion as a weighted sum makes the MGA more likely to get stuck in a local (non)optimum. However, having each error as a separate dimension of a fitness function yields a Pareto front where the vast majority of creatures are useless (because being useless in a single dimension tends to be sufficient for the resulting model to be useless). As a result, the evolution tends to explore mostly useless creatures, and does not progress well. In practice we found that 3 fitness dimensions (in which the errors are allocated as weighted sums) was a sweet spot for most of the genetic algorithms, with 2 and 4 also useful[a]. We often aggregated logically connected errors in the same dimension, i.e., fitness(1) would consider errors of basic CaT biomarkers, fitness(2) would consider alternans errors, etc.
- **Population size and number of generations.** We used a two-stage procedure, where a large[b] population was first simulated for several (5-10) generations. The resulting Pareto front was used as a starting population for a second stage MGA, which was ran with a smaller population of 300 or 450 creatures. Using a large population has the advantage that it samples the parameter space well initially, and it explores a more diverse population than if a small population was used at the start. However, a large population subsequently undergoes slow and inefficient evolution. On the other hand, using a smaller population accelerates the evolution (with a single generation taking much less time to simulate) – but if used exclusively, the initial parameter space would not be sampled as well. The two-stage procedure combines good sampling of initial parameter space with reasonably fast evolution later.
- **Final choice of creatures for further development.** A multiobjective GA produces a Pareto front, rather than a single creature at the end of optimization, with us having to choose one solution for further development and/or final selection. With 4-dimensional fitness that we used at the end of the development process, and a small population, only few creatures were practically of relevance: we ignored all those with imperfect alternans properties, lack of restitution with slope >1 that steepens with APD prolongation, those not fulfilling criteria on duration of CaT, as well time to peak of dyadic and cytosolic calcium, and those that did not manifest EADs in the relevant conditions. From the few creatures left (which were similar in parameters), we chose one with the best APD and CaT morphology.
- **Multiple runs of GA to avoid local optima.** Even though multiobjective GAs are less prone to getting stuck in a local optimum than standard single-objective one, this remains a potential concern. We have run key instances of GA in several repeats, monitoring differences in solutions achieved. Throughout the development, the solutions/creatures produced were highly similar, both with regards to the fitness achieved, and creatures evolved, indicating that evolution stochasticity was not a strong limiting factor.

---

[a] Final development MGAs were run with the most complex fitness that had 4 dimensions. Early model development would consider many fewer criteria, and would use only 2 dimensions, with most development done with fitness of intermediate complexity and 3 dimensions.
[b] What "large" means depends on the problem and fitness function. For our more comprehensive simulations where a single fitness evaluation took around 3 minutes, a population of 3600 creatures was considered large. However, for faster-to-run fitness functions, hundreds of thousands creatures can be simulated.

- **Early killing of ultimately infeasible creatures.** Early in the evolution, it is important to consider even creatures with fitness that will be ultimately deemed useless (e.g. models excellent in fitness(2) and fitness(3), but very poor in fitness(1)). While the creatures are not yielding useful models themselves, they can carry valuable information that can be combined with other creatures in crossover, creating excellent creatures and thus models in the future. However, in the second stage of optimization, which is more about accelerating evolution and parameter fine-tuning, it may be practical to avoid having too many creatures that will be ultimately deemed useless. To this end, we would sometimes include guards that set fitness to Inf (effectively guaranteeing the removal of such creatures from the evolved population) when any of the fitness dimensions is bad enough. Similarly, when a single error that is optimized is known to often drive such infeasibility, it is worth to calculate this error at the start of the fitness function and check right after what the error value is – if it is poor, the entire fitness can be stopped there (returning vector of Inf to make sure the creature is discarded), before the rest of the fitness function is simulated, which saves time.
- **Saving intermediate results**. It is very useful to save every generation throughout the optimization, which can be achieved by using the 'OutputFcn' option of @gamultiobj. This enables intermediate monitoring of the evolution, early termination of optimization without losing any results, or restarting the evolution from the last saved point e.g. in case of power outages.
- **Pareto front size limit.** We observed that the Matlab implementation of MGA seems to have an internal limit of the Pareto front size. As a result, when the Pareto front size reaches 350, some creatures start being removed throughout the generation, so that the size of Pareto front is maintained. For some reasons, we noted that the removed creatures would be often the ones that encode the practically best models. For this reason, we suggest that the number of creatures on the Pareto front is monitored throughout the simulation, and caution is exercised when the limit of 350 is hit – it is possible that good creatures are lost from that point on. This issue is exacerbated with a growing number of dimensions of the fitness function. It can be partly ameliorated by the early discarding of creatures described above, which generally limits the space of feasibility of fitness values.

Details of optimization fitness

Two fitness functions were used to constrain properties of the calcium handling system, and of L-type calcium current and overall cellular behavior. They were run repeatedly, for different intermediate versions of the model with different formulations of ionic currents etc. The two fitness functions are partly independent, and e.g. models strong in the first fitness for calcium handling did retain their strengths after undergoing fitting for the second fitness (largely due to different parameters of the model being evolved). This separation into two relatively independent optimizations enabled us to fit the model to a large set of criteria, and use a still relatively low-dimensional fitness. To further ascertain that the latter optimization does not fundamentally alter the model with regards to the first set of criteria, several elements of the first fitness were included in the second one. Convergence towards useful models would be challenging if all the optimization criteria were included in a single fitness function, which would also consequently take considerably longer to run. We note that the ability to split the optimization into multiple parts like this strongly relies on model robustness, where properties achieved after one optimization are largely retained when undergoing the other one and vice versa.

Fitness for calcium handling

The following four-dimensional fitness (to be minimized) was used to evolve parameters of calcium handling. Unless stated otherwise, fitness is calculated based on 1 Hz pacing simulations. Each fitness dimension further aggregates multiple penalties/errors. Below is given the final fitness. This has been built over time – early in the development, the model was evolved with fewer criteria (e.g. initially making sure that alternans is present at rapid pacing, with roughly reasonable amplitudes and duration of CaTA at 1 Hz), and more were added as model was improved – until the final version below was used.

In many cases, you will see the errors have the form of X+Y*(feature-reference) if the feature (e.g. CaT amplitude) is outside the desired range. This helps the evolution to find satisfactory solutions. If only a fixed-constant penalty was used (the X term), this is usually not sufficiently informative for the evolution process, as a model slightly outside the desired range will have the same penalty as a model that is completely off. On the other hand, if only the Y*(feature-reference) is used, the evolution can struggle balancing small errors in the weighted sums, often producing models that are slightly off in multiple features. Combining a flat penalty with a dynamic one avoids those issues, helping the evolution find solutions within biomarker ranges[c].

- $fitness(1) = error_{CaTduration} + error_{Ca,dyad} + error_{Ca,dyad\ decay} + error_{unsmoothness} + error_{SERCA,slope} + error_{Ca,diastolic} + error_{Ca,diastolic3.3Hz} + error_{CaTamplitude} + error_{CaTamplitudeBcl260} + error_{CaSR} + error_{CaSR,decay} + error_{excitation} + error_{TTP} + error_{TTP,RyRO} + error_{contraction}$
    - $error_{CaTduration} =$
        - $4 * (CaTD90 - 400)^2\ if\ CaTD90 < 380$ (CaTD90 is like APD90, but for CaT)
        - $(CaTD90 - 400)^2\ if\ CaTD90 < 390\ \&\ \geq 380$
        - $(CaTD90 - 410)^2\ if\ CaTD90 > 430$
        - $0\ otherwise$
    - $error_{Ca,dyad} =$
        - $0\ if\ \max(Ca_{dyad}) < 0.1$ (this is in mM, so 100 μM)
        - $50 + \left(\max(Ca_{dyad}) - 0.1\right) * 1E4\ for\ \max(Ca_{dyad}) \in [0.1, 0.11)$
        - $250 + \left(\max(Ca_{dyad}) - 0.1\right) * 1E4\ for\ \max(Ca_{dyad}) \in [0.11, 0.125)$
        - $500 + \left(\max(Ca_{dyad}) - 0.1\right) * 1E4\ otherwise$
    - $error_{Ca,dyad\ decay}$ serves to avoid two-peak dyadic calcium; i.e., the derivative of dyadic calcium should be negative or almost-negative from the peak until the end of the AP. It is defined as follows:
        - $5000\ if\ \frac{dCa_{dyad,afterPeak}}{dt} > 3E\text{-}7$. $Ca_{dyad,afterpeak}$ is the dyadic Ca from its peak to the end of recording
        - Additional $\max\left(\frac{dCa_{dyad,afterPeak}}{dt}\right) + 5$ is added if $\frac{dCa_{dyad,afterPeak}}{dt} > 1E\text{-}6$. This is a marginal additional penalty only, slightly helping the evolution approach feasible solutions.

[c] That said, several error terms without the fixed penalty can work fine and are used here, and a large flat penalty can be also used to signal a generally problematic property of the model in certain situations – it is not always necessary to include both terms.

- $error_{unsmoothness}$ penalizes overly biphasic rise of cellular calcium transient. Linear models are fit to $[Ca]_i$ signal between 7-10.5 ms and between 12-19.5 ms (those intervals are furthermore shifted by the difference between time to peak dyadic calcium and 13). Subsequently,

$$error_{unsmoothness} = 5E12 * \left(slope_{segment1} - slope_{segment2}\right)^2$$

- $error_{SERCA,slope}$ penalizes the lack of positive relationship between SERCA pump amount and CaT amplitude (CaTA). It starts as zero, with additional penalties added as follows:
  - A linear model is fit to x=[0.5,1,2], y=[$CaTA_{halfSERCA}$, $CaTA_{normalSERCA}$, $CaTA_{doubleSERCA}$]. If slope is lower than 0.2, $error_{SERCA,slope} = 100 + (0.2 - slope) * 100$
  - Further 600 is added if slope < 0.
  - Further 500 is added if $\mathrm{CaTA_{halfSERCA}} * 1.2 > \mathrm{CaTA}_{normalSERCA}$
- $error_{Ca,diastolic} =$
  - $0\ if\ Ca_{i,diastolic} \in [0.9E\text{-}4, 1.27E\text{-}4]$ (in mM, so 90-127 nM)
  - $300 + 1E12 * \left(Ca_{i,diastolic} - 1E\text{-}4\right)^2\ otherwise$
- $error_{Ca,diastolic3.3Hz}$ is similar to the preceding error, but is taken at 3.3 Hz (basic cycle length of 300 ms), rather than 1 Hz stimulation (we want to see an increase in resting Ca). It is:
  - $0\ if\ Ca_{i,diastolic,3.3Hz} \in [1.7E\text{-}4, 2.3E\text{-}4]$
  - $300 + 5E10 * \left(Ca_{i,diastolic,3Hz} - 2E\text{-}4\right)^2\ otherwise$
- $error_{CaTamplitude} =$
  - $0\ if\ CaT_{amplitude} \in [3.4E\text{-}4, 3.7E\text{-}4]$ (in mM, so 340-370 nM)
  - $200 + 2E11 * \left(CaT_{amplitude} - 3.5E\text{-}4\right)^2\ otherwise$
- $error_{CaTamplitudeBcl260}$ penalizes too small CaT in smaller beat in alternans (at 260 ms basic cycle length pacing), preventing 2:1 Ca block. If the smaller of two last beats at this pacing rate is larger than 60 nM, this criterion is 0, otherwise it is 1000.
- $error_{CaSR} =$
  - $0\ if\ Ca_{SR,diastolic} > 0.6$
  - $500 + 3000 * \left(0.6 - Ca_{SR,diastolic}\right)\quad otherwise$
- $error_{CaSR,decay}$ penalizes decreasing SR content at the end of a beat at 1 Hz pacing. To calculate it, differences between last 10 samples of Ca_{SR} of a beat are calculated, and the minimum is taken ($minimum_{dcl}$). Then, $error_{CaSR,decay} =$
  - $0\ if\ minimum_{dcl} \geq 0.45E\text{-}4$
  - $500 - 1E5 * (500 - minimum_{dcl})\quad otherwise$
- $error_{excitation}$ penalizes instability (lack of regular CaT or regular alternans at 260 ms basic cycle length). If there are five continuous segments in the membrane potential signal below -80 mV when simulating last 4 beats of pacing at 260 ms bcl (corresponding to 4 well-developed APs), this error is 0, and it is 750 otherwise.
- $error_{TTP}$ aggregates numerous aspects of time to peak (TTP) of calcium in the dyad and cytosol, at different pacing rates. The starting value is

- $error_{TTP} = abs\left(TTP_{Ca,dyad,1Hz} - 13\right)^{1.5}$ (aiming for ca. 13 ms TTP in the dyad). To this are added additional penalties:
- $(mean\left(TTP_{Ca,dyad,260msBcl}\right) - 20)^2 \; if \; mean\left(TTP_{Ca,dyad,260msBcl}\right) > 35$ (i.e., if average time to peak at rapid pacing – possibly with alternans, hence the average is calculated - is too large, a penalty is applied)
- $100 + 3 * \left(TTP_{Ca,i,1Hz} - 60\right)^2 if \; TTP_{Ca,i,1Hz} < 47$ (penalizing too short TTP of cytosolic CaT)
- $100 + 5 * \left(TTP_{Ca,i,1Hz} - 50\right)^2 if \; TTP_{Ca,i,1Hz} > 60$

  - $error_{TTP,RyRO} =$
    - $0 \; if \; TTP \; of \; O \; state \; of \; the \; RyR \; model \; is < 15 \; ms$
    - $350 + 30 * \left(TTP_{RyR,O} - 14\right)$. This error term helps make sure that the opening of the Ca-sensitive RyR is sufficiently rapid (TTP of dyadic Ca could be in principle achieved merely via the I_{CaL}-coupled RyRs, which we wanted to avoid).
  - $error_{contraction}$ constrains time to peak contraction and rt95 (time from peak to 95% recovery). It starts as zero, with two penalties optionally added:
    - $100 + 0.3 * (TTP_{contraction} - 165)^2 \; if \; TTP_{contraction} \; \notin [155{,}165]$
    - $100 + 0.3 * (rt95 - 330)^2 \; if \; rt95 \; \notin [320{,}350]$
- $fitness(2) = error_{CaSR,decay} + error_{stabilization} + error_{NCX}$
  - $error_{CaSR,decay}$ is defined as above
  - $error_{stabilization}$ penalizes too long oscillation with abrupt change in pacing rate from 2 to 3 Hz. The differences between CaT amplitude in consecutive beats in 50 beats after such change are calculated, and this error term is equal to the first instance of such difference being between -5 and 0 ("almost stabilize") – by minimizing this, such almost-stabilization should occur as early as possible. If stabilization does not occur throughout the 50 beats, this means a generally unstable model and a penalty of $1000 + 10 * abs\left(difference_{CaTA,last\;2\;beats}\right)$ is added.
  - $error_{NCX}$ serves to prevent the NCX going positive during plateau, which was a concern at one point in the development with another model of NCX. To calculate this, t50 is the time vector from 50 ms on, and ncx50 is the NCX current from 50 ms on, where negative values were set to 0 (i.e., the only remaining values are positive ones and zeros). Subsequently, $error_{NCX} = trapz(t50, ncx50)$, i.e., the area under the curve given by t50 and ncx50.
- $fitness(3) = 1.5 * error_{rateCaSR} + 4 * error_{rateCaT} + error_{rateContraction}$ This dimension constrains properties of Ca handling across 1 Hz, 2 Hz, and 3.3 Hz.
  - $error_{rateCaSR}$ starts as zero, to which are added the following penalties:
    - $200 + 1E5 * \left(1.05 * Ca_{SR,diastolic,1hz} - Ca_{SR,diastolic,2hz}\right),$
      $if\left(1.05 * Ca_{SR,diastolic,1hz} - Ca_{SR,diastolic,2hz}\right) > 0$
    - $200 + 1E5 * \left(1.03 * Ca_{SR,diastolic,2hz} - Ca_{SR,diastolic,3hz}\right),$
      $if\left(1.03 * Ca_{SR,diastolic,2hz} - Ca_{SR,diastolic,3hz}\right) > 0$
  - $error_{rateCaT}$ starts as zero, to which are added the following penalties:

- $200 + 5E6 * \left(1.05 * Ca_{i,amplitude,1hz} - Ca_{i,amplitude,2hz}\right),$ $if\left(1.05 * Ca_{i,amplitude,1hz} - Ca_{i,amplitude,2hz}\right) > 0$
- $200 + 5E6 * \left(1.05 * Ca_{i,amplitude,2hz} - Ca_{i,amplitude,3hz}\right),$ $if\left(1.05 * Ca_{i,amplitude,2hz} - Ca_{i,amplitude,3hz}\right) > 0$

  - $error_{rateContraction}$ starts as zero, to which are added the following penalties:
    - $200 + 100 * (1.1 * DevelopedTension_{1Hz} - DevelopedTension_{2Hz}),$ $if(1.1 * DevelopedTension_{1Hz} - DevelopedTension_{2Hz}) > 0$
    - $200 + 10 * (1.03 * DevelopedTension_{2Hz} - DevelopedTension_{3Hz}),$ $if(1.03 * DevelopedTension_{2Hz} - DevelopedTension_{3Hz}) > 0$

- $fitness(4) = 100 * error_{S2CaT} + error_{alternans} + 4 * error_{alternansModulation} + error_{CaSR,decay}$
  - $error_{S2CaT}$ helps ameliorate excessive refractoriness of the Ca transient. It is defined using the variable S2S1ratio, which represents the ratio of the CaT amplitude from a premature beat applied 500 ms after 1 Hz prepacing to the CaT amplitude during steady-state 1 Hz pacing. $error_{S2CaT} =$
    - $0 \ if \ S1S2ratio \geq 0.65$
    - $100 * (0.65 - s2s1ratio) \ if \ S1S2ratio \in (0.25, 0.65)$
    - $500 + 100 * (0.65 - s2s1ratio) \ if \ S1S2ratio \leq 0.25$
  - $error_{alternans}$ describes the presence of alternans in a control cell at basic cycle length of 260 ms. It is defined using alternansRatio34 variable, which is the ratio between CaT amplitude of the 3rd and 4th beats in the four beats recorded at the end of the simulation of rapid pacing. $error_{alternans} =$
    - $500 + 2000 * (\text{alternansRatio34} - 0.5) \ if \ alternansRatio34 \ lies \ outside \ 0.2 \ and \ 0.8$
    - In addition, 666 is added if the ratio between 1st and 3rd beat in the last four simulated , or the ratio between 2nd and 4th (alternansRatio24), is <0.99. This indicates overall instability, rather than regular alternans.
  - $error_{alternansModulation}$ penalizes additional properties of alternans.
    - A penalty of 600 is added if alternansRatio34 (between 3rd and 4th CaT amplitude) is <0.99 at 300 ms basic cycle length. I.e., this serves to avoid alternans presence at 300 ms bcl.
    - A penalty of 1600 is added if clear alternans is not present at 450 ms bcl in a model with 65% SERCA (alternansRatio34 between 0.2 and 0.8, and alternansRatio13 and alternansRatio24 above 0.99).
  - $error_{CaSR,decay}$ is defined as in previous fitness dimensions.

In addition, any model with one of the following properties is considered infeasible and its fitness is set to infinity in all dimensions:

- More than 1 calcium transient upstroke at 1 Hz pacing (unstable model).
- More than 4 calcium transient upstrokes in 4 beats simulated at 260 ms basic cycle length.
- If the SR content changes by more than 1% between the start and the end of the last simulated beat (another index of instability).

- Simulations crash at any point.

The following model parameters were evolved using this fitness:

- Parameters of SERCA pumps: $V_{max,SERCA}, K_{mr}, K_{mf}, H\ (Hill\ coefficient)$, Vmax increase by calcium-mediated activation.
- Parameters of the main calcium-sensitive RyR: k_s (multiplier of calcium-sensitive RyR flux), $k_{o,Ca}, k_{om}, k_{i,Ca}, k_{im}, EC50SR$ (midpoint of I_{CaL}-coupled RyR activation), parameters of $transition_{RI\to CI}$ not equal to 1, reverse rate $transition_{CI\to RI}$, constants involved in calculation of $transition_{R\to O\&RI\to I}, transition_{O\to I\&R\to RI}$.
- Parameters of I_{CaL}-coupled RyR activation: overall magnitude, b_t (used to calculate the time constant), exponent in $J_{rel,ICaLdep,act,\infty}$, time constants of inactivation (denominators in $\frac{dJ_{rel,ICaLdep,f1}}{dt}$ and $\frac{dJ_{rel,ICaLdep,f2}}{dt}$)
- Buffering parameters: $B_{max,CSQN}, B_{max,SR}, B_{max,SL,low,subsarcolemmal}, B_{max,SL,high,subsarcolemmal}, B_{max,SL,low,dyadic}, B_{max,SL,high,subsarcolemmal}$. Here, for $B_{max,SL}$, the same constant was used for low and high constant in each compartment.
- NCX fraction in the dyad.
- Multiplier of I_{Cab}.
- Multipliers of parameters in the Land model of contraction: ca50 (midpoint of calcium binding), mu, nu, k_{off}, $ktm_{unblock}$.

Fitness for I_{CaL} and overall model behavior

The following four-dimensional fitness (to be minimized) was used to evolve parameters of I_{CaL} and the balance of ionic currents. It aims to achieve reasonable properties of AP, I_{CaL}, and restitution, while not breaking key features of calcium handling.

- $fitness(1) = error_{AP} + error_{IV,ICaL} + error_{restitution,IKrBlock,1} + error_{restitution,IKrBlock,2} + error_{IKr,density} + error_{CaTamplitude} + error_{TTP}$
  - $error_{AP}$ starts as zero, with the following three components added optionally. Here, $plateau_{max}$, the maximum plateau potential, is defined as the maximum membrane potential from 20 ms on. The notation V_t denotes membrane potential at time t.
    - $100 + 100 * (plateau_{max} - 25)^2\ if\ plateau_{max} > 25$
    - $25 + 25 * (plateau_{max} - 23)^2\ if\ plateau_{max} > 23$
    - $1000 * (V_{60} - V_{25} + 2)\ if\ - V_{60} > V_{25} - 2$
  - $error_{IV,ICaL}$ is obtained by simulating the I_{CaL} I-V relationship as in Magyar et al. [103]. The sum of squares of the simulation versus the experimental data is multiplied by 16 to get this error term. In addition, if the peak current exceeds that of the data by more than 5%, the error is multiplied by 8 (i.e., the sum of squares times 128)
  - $error_{restitution,IKrBlock,1}$ makes the model steepen its restitution following I_{Kr} inhibition (to 60% availability). It is calculated based on peak restitution slope in a control versus I_{Kr}-blocked cell.

$$error_{restitution,IKrBlock,1} =$$

$2000 * (0.5 + 1.1 * \max(slope_{control}) - \max(slope_{60\%IKr}))\ if$
$1.075 * \max(slope_{control}) > \max(slope_{60\%IKr})$

- $error_{restitution,IKrBlock,2}$ is very similar to the preceding error term, except it is measured at 50% I_{Kr} availability:

  $error_{restitution,IKrBlock,2} =$
  $1000 * (0.5 + 1.15 * \max(slope_{control}) - \max(slope_{60\%IKr}))\ if$
  $1.12 * \max(slope_{control}) > \max(slope_{60\%IKr})$

  The reason for including two highly similar error terms differing only in a small amount of I_{Kr} block is the fact that the exact value of peak restitution slope depends on where exactly the S2 stimuli (spaced 5 ms apart) hit the membrane potential of the recovering cell – as a result, small changes in input parameters can occasionally produce a nontrivial change in the peak slope near the effective refractory period. We noticed that when only one term for I_{Kr} modulation was included, the optimization procedure sometimes leveraged this phenomenon and produced a model which technically steepened its restitution in the specific simulated condition, but this was not a robust steepening (the property of I_{Kr}-induced restitution steepening would be lost with perturbation of model parameters). In contrast, having two criteria constraining this property at 50% and 60% I_{Kr} availability was sufficient for the genetic algorithm to find solutions where the restitution steepening was a robust property, present even under model perturbation.
- $error_{IKr,density}$ is a relatively technical parameter serving for the evolution to avoid models increasing I_{Kr} density compared to the previous development version. Based on the evolved parameter $multiplier_{IKr}$, it is defined as:
  $error_{IKr,density} = 50 + 1000 * (multiplier_{IKr} - 0.9)\ if\ multiplier_{IKr} > 0.9$
- $error_{CaTamplitude}, error_{TTP}$ are defined as in the fitness function constraining calcium handling.

- $fitness(2) = error_{EADs} + error_{P2P1} + error_{ICaL,decay} + error_{VDI}$
  - $error_{EADs}$ starts with the value 3000 (to become 0 in the presence of satisfactory EADs), with the following changes being made, largely depending on $\max(diff_{V300})$, which is the maximum dV/dt from 300 ms on, extracted from a simulation of the evolved model at 0.25 Hz, 17% I_{Kr}, and 2 mM extracellular calcium.
    - 1000 is subtracted if $\max(diff_{V300}) > 0.15$ (i.e., an afterdepolarization is present)
    - 1000 is subtracted if $\max(diff_{V300}) < 0.6$ (too steep afterdepolarizations are avoided)
    - 1000 is subtracted if membrane potential at 2000 ms is below -80 (the cell depolarizes)
    - 500 is added if $\max(diff_{V300}) < 0.1$ (no or near-flat EAD is produced)
  - $error_{P2P1}$ is the sum of three subterms which are added together:
    - Sum of square differences between simulation of the P2P1 protocol and experimental data using P1=25 ms (**as in Supplementary Figure S12B**)

- Sum of square differences between simulation of the P2P1 protocol and experimental data using P1=100 ms (**as in Supplementary Figure S12B**)
- $0.025 + \left(0.1 - abs\left(fraction_{ICaL,P1=25,interpulse=150} - fraction_{ICaL,P1=100,interpulse=150}\right)\right) if$ $abs\left(fraction_{ICaL,P1=25,interpulse=150} - fraction_{ICaL,P1=100,interpulse=150}\right) < 0.1$, where $fraction_{ICaL,P1=X,interpulse=Y}$ represent the fraction of recovered I_{CaL} using P1 duration of X ms, and interpulse interval of Y ms. This term helps to achieve sufficient distinction between recovery from refractoriness when 25 ms versus 100 ms inactivating pulse is employed.

  - $error_{ICaL,decay} = 200 - \min\left(\frac{dI_{CaL,20-}}{dt}\right), if \min\left(\frac{dI_{CaL,20-}}{dt}\right) < 0$, where $\frac{dI_{CaL,20-}}{dt}$ is the derivative of I_{CaL} measured under voltage clamp (same protocol as IV protocol extraction), from 20 ms after a holding potential of 0.1 mV is imposed to the end of the pulse. The error term penalizes I_{CaL} that does not decay monotonically (instead, its derivative should be positive throughout). This serves to constrain CDI – a too strong CDI that recovers too early could in principle generate I_{CaL} model which activates, then strongly inactivates, and then again increases due to I_{CaL} recovery from refractoriness. This would generate I_{CaL} curves unlike those seen in data, but is prevented by this criterion.
  - $error_{VDI}$ weakly constrains the voltage-dependent inactivation of I_{CaL}, having the value of 0.5 if the fraction of available I_{CaL} is more than 0.7 after 50ms pulse of 20 mV; otherwise it is 0.
  - We note that the scaling of the errors in fitness(2) is such that the EAD penalty is much greater than the other ones – as a result, the genetic algorithm first focuses on finding solutions that are EAD-capable, and subsequently pursues the other properties.
- $fitness(3) = error_{fatICaL} + error_{CaTduration}$
  - $error_{fatICaL}$ is based on $I_{CaL,175}$, which is the I_{CaL} current at 175 ms at 1 Hz pacing. The value of this error term is as follows, penalizing I_{CaL} that decays too slowly within an AP.
    - $500 - I_{CaL,175} * 100$ if $I_{CaL,175}$ $if\ I_{CaL,175} < -0.3$
    - $50 - I_{CaL,175} * 20$ if $I_{CaL,175}$ $if\ I_{CaL,175} < -0.15$
  - $error_{CaTduration}$ is defined as in the fitness function constraining calcium handling.
- $fitness(4) = error_{restitution}$
  - $error_{restitution}$ starts as zero, with the following penalties added, based on peak S1S2 restitution slope ($slope_{restitution,max}$) recorded
    - 1000 if peak restitution slope exceeds 3 (usually indicating some form of instability.
    - $1000 + 2000 * \left(1.1 - slope_{restitution,max}\right)\ if\ slope_{restitution,max} < 1$
    - $200 + 2000 * \left(0.7 - slope_{restitution,aftermax}\right)\ if\ slope_{restitution,aftermax} < 0.7$

Here, $slope_{restitution,aftermax}$ is the restitution slope measured at the next S2 coupling interval, 5 ms longer than the one where maximum was achieved. I.e., this penalty forces the restitution to be overall relatively steep near its maximum. This serves to prevent the scenario (which we observed when this penalty was not included) where a model with a flat restitution except one steep point can technically fulfill the criterion of "peak slope > 1", while not practically matching relevant reality.

The following model parameters were evolved using this fitness:

- Multipliers of I_{Kr}, I_{CaL}, I_{ClCa}, I_{Clb}, I_{Ks}, I_{NaL}, I_{NaK}, I_{pCa}, I_{Kb}, I_{to}
- Constants present in the model of I_{CaL}:
  - All constants not equal to 1 in equations for voltage-independent CDI of I_{CaL}: $f_{\infty Ca,CDI,X}$ and $\tau_{f,Ca,CDI,X}$ and $r_{recovery}$. The same values evolved were used for dyadic and nondyadic I_{CaL}.
  - All constants added to membrane potential were evolved by adding an evolution-determined number in the following equations:
    $$d_\infty, \tau_{f,fast}, \tau_{f,slow}, \tau_{f,Ca,fast}, \tau_{f,Ca,slow}, A_{f,Ca,fast}, jca_\infty$$
    Addition rather than multiplication serves to avoid disproportionately larger shifts in membrane potential in equations where the shifting constant is larger, compared to equations where the initial value of the shifting constant was small.
  - All constants not equal to 1 that are not shifters for membrane potential were evolved by a multiplication actor in the following equations:
    $$\tau_{f,fast}, \tau_{f,slow}, \tau_{f,Ca,fast}, \tau_{f,Ca,slow}, jca_\infty, \alpha_{n,dyad}$$
  - Constant in $A_{f,Ca,fast}$ that is now 27.96201 was evolved by a multiplicative factor.
  - The following constants were evolved by a multiplication factor: $A_{f,fast}, \tau_{jca}, K_{m,n}, K_{+2,n}, K_{-2,n}$.

Notes on implementation

The default implementation is in Matlab, which was subsequently converted to CellML, C, and CUDA, enabling loading T-World in simulators such as Chaste[157], MonoAlg3D[158], or Myokit [156], with the latter also offering conversion to other languages.

The Matlab version is structured with a logic similar to ToR-ORd [4]:

1. The user defines parameters of the simulation (e.g. number of beats, stimulation rate, concentrations, multipliers of current conductances, etc.) in a Matlab simulation script, which then calls a runner function. When the simulation is finished, a structure with ionic currents and fluxes over time is extracted and may be easily used to visualize traces (e.g. 'plot(currents.time, currents.V)' to plot membrane potential, etc.).
2. The runner function receives the parameters, unpacks them into single variables, and sets the undefined parameters to default values (in this way, the user has to define only the parameters that do not take the default value). Subsequently it solves the ordinary differential equation system in a model function using the ode15s solver.

3. The model function, containing the definition of the cell model, calculates the derivatives of state variables based on the input state variables vector.

This design has several advantages. We believe that it maximizes user comfort by allowing them to define a simple structure of parameters as an input, without them having to touch the model code itself. Using structures is convenient to neatly pack Matlab variables. Unfortunately, their use in Matlab also comes with a rather severe performance penalty. For this reason, we use them only on the user side for convenience; however, the runner function unpacks them into single, unstructured variables, which are then passed to the ODE solver itself. Unpacking the structure once does not cause any performance problem – however, using the structure of parameters in the model function would, which is why we avoid this.

An advantage of the model function being kept separate is that different model functions can be easily used in the Matlab framework we provide. E.g. a model function specific to a disease may be created by the operator, and then used by including in the structure of parameters which model function should be used (e.g. 'parameters.model = @model_HFrEF;').

An additional aspect of modularity is that the model function of T-World is structured logically into functions calculating different ionic currents and other components (in contrast to some other models using monolithic code). It is therefore easy to navigate the code, and to replace a chosen ionic current by including a different formulation as a function. We also use reasonably named variables throughout the code; e.g. 'ions_ca_i' referring to intracellular calcium concentration. This is in contrast with many other models which refer to elements of the state vector (e.g. y(38) or X(8), which is difficult to navigate for researchers not familiar with the given model family.

Finally, we avoided Matlab global variables in T-World, given their performance penalty and given that they complicate parallelization of simulations. The one exception is the version of T-World with stochastic DADs, where we were unable to avoid their use when relying on ode15s for ODE solving.

Other models

T-World was compared in this study to ToR-ORd [4], Morotti 2021 [11], and TP06 models [12]. ToR-ORd was downloaded from Github (https://github.com/jtmff/torord), as was the Morotti 2021 model (https://github.com/drgrandilab/Morotti-et-al-2021-Cross-species-translators-of-electrophysiological-response). The endocardial TP06 model was simulated using a CellML version (https://models.cellml.org/workspace/tentusscher_panfilov_2006) loaded and simulated in Myokit [156].

Experimental methods

Experimental data for human S1S2 restitution, used to investigate sex differences in restitution slope, were collected as a part of prior study[83], with methodology described there.

Supplementary notes

Supplementary note 1: Extracellular calcium and APD

Our model does not reproduce the known phenomenon of extracellular calcium shortening APD (not shown), a limitation shared by most cardiomyocyte models, with mechanism unknown. If the role of extracellular calcium is a central focus of a study, the BPS2020 model[159] may be used, which in addition also recapitulates several arrhythmic behaviors. That said, the action potential shape is not particularly human-like, and the reported alternans is not a typical

calcium-driven alternans, but results from calcium transient developing only in every other beat, which is then naturally translated into APD oscillation. Additionally, as the model relies on strong calcium-dependent inactivation of I_{CaL}, the resulting "alternans" exhibits electromechanical discordance (longer APD in CaT-absent beats). This contradicts most data in human-like species [30,70,71], and while I_{CaL} inactivation likely contributes to extracellular calcium responses, further research is needed to confirm its central role.

Supplementary figures

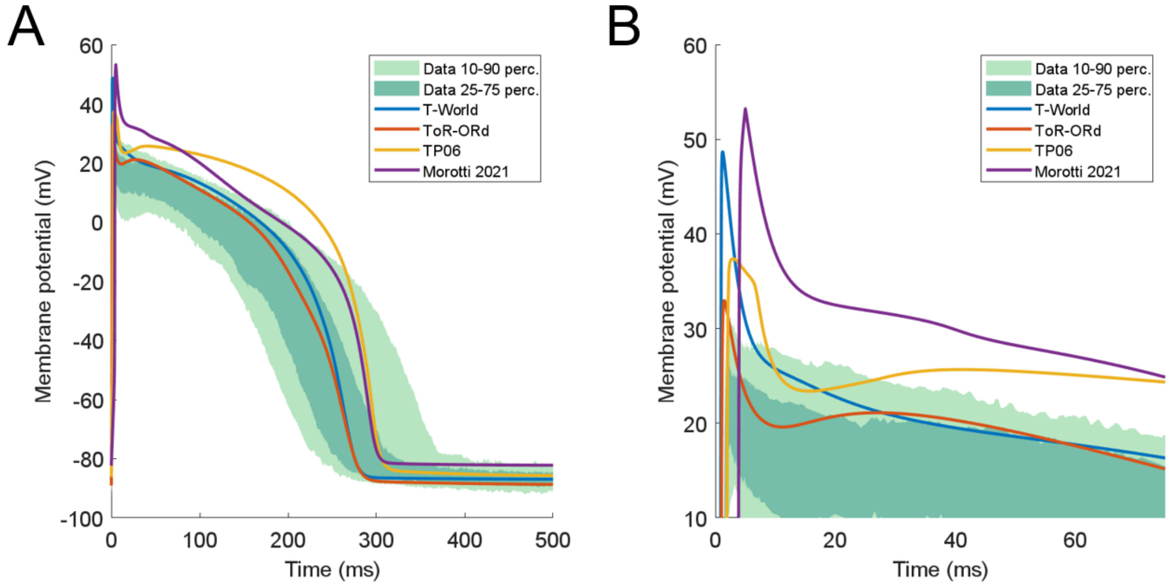

Figure S6. Comparison of AP morphology between models. A) *Comparison of action potentials (APs) between four major computational human ventricular cardiomyocyte models and experimental data [18]. **B)** A zoomed-in version panel A, showing a notch following the AP peak in ToR-ORd and TP06 models, that is typically not seen in endocardial cells. In addition, TP06 model shows unusually biphasic transition from peak to the notch and a lack of AP triangulation, with a long, near-flat plateau followed by a steep repolarization. The Morotti2021 model also does not manifest a notch after the AP peak, but it shows a kink in the early plateau, which is generally not observed in living cells, and which results from a calcium-handling issue inherent to this model family (discussed in main manuscript).*

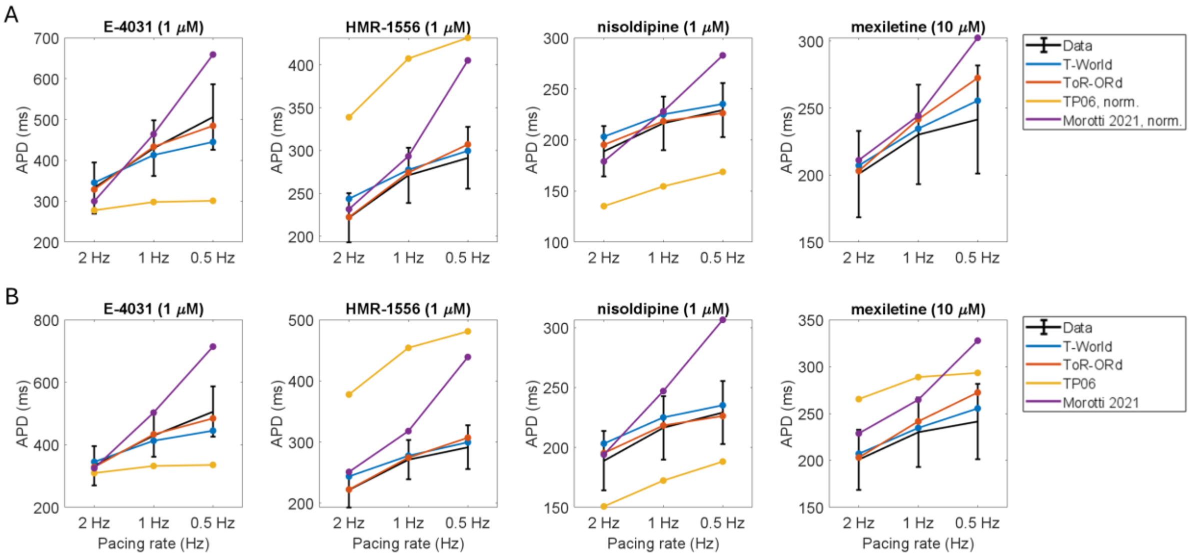

Figure S7. Comparison of the response of four models to simulated channel blocking drugs. *Independent validation of the APD prolongation or shortening induced by 1 µM E-4031 (70% I_{Kr} block), 1 µM HMR-1556 (90% I_{Ks} block), 1 µM nisoldipine (90% I_{CaL} block), and 10 µM mexiletine (54% I_{NaL}, 9% I_{Kr}, 20% I_{CaL} block) at 0.5, 1.0 and 2.0 Hz pacing in the four models. Drug concentrations and their effects on channel blocks are based on O'Hara et al. [18]. In panel **A**, outputs of the TP06 and Morotti2021 models were scaled by $APD90_{model}$/270 to compensate for their slightly longer baseline APD: without the normalization, the discrepancy between those models and data is exaggerated artificially. The outputs of TP06 are not shown for mexiletine, as the model lacks the representation of the primary target I_{NaL}. For completeness, the version of the figure without normalization and with TP06+mexiletine is given in panel **B**. Please note the distinct y-axes for the four drugs.*

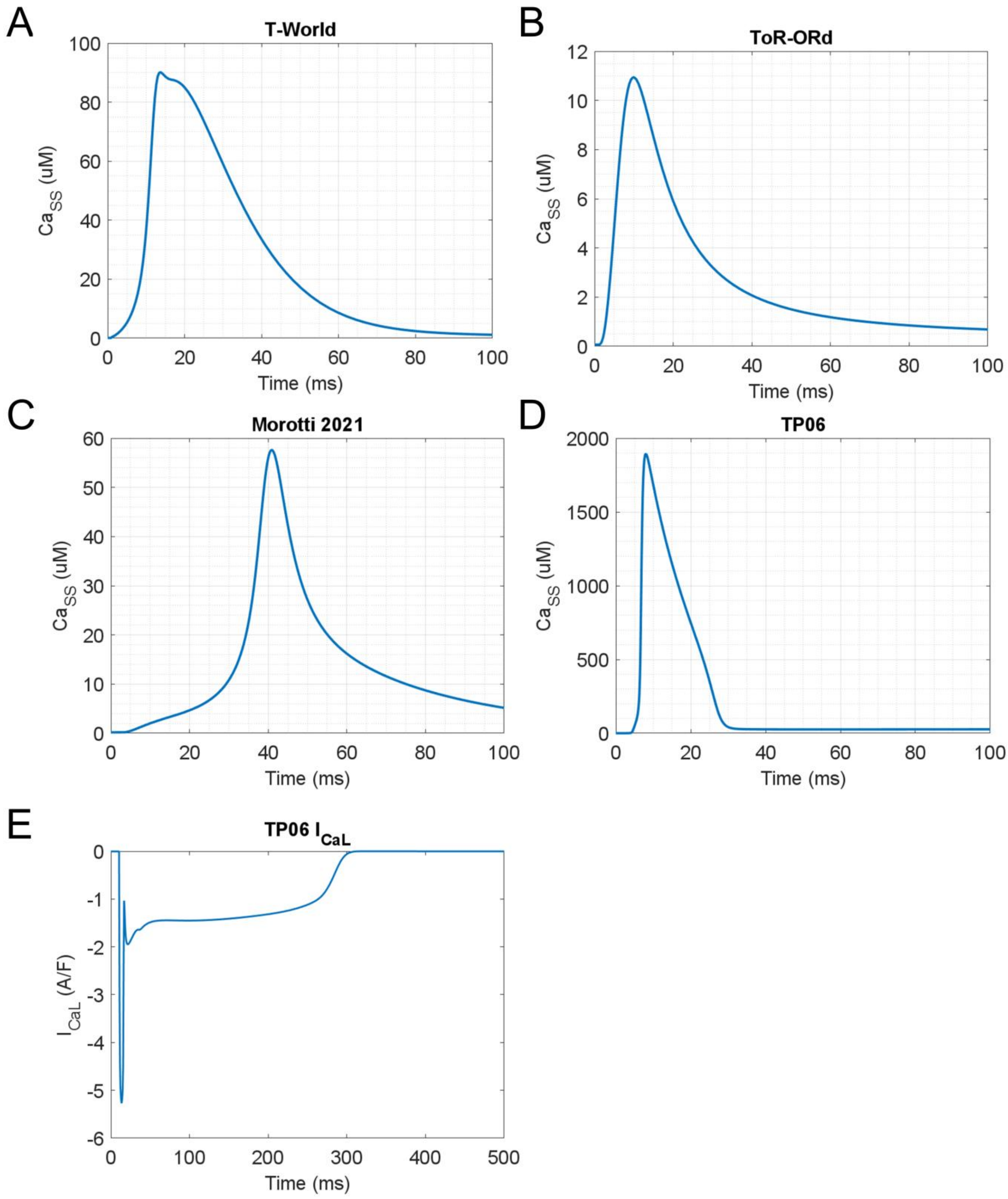

Figure S8. Dyadic concentrations of calcium in four models. A-D) *dyadic calcium concentrations in T-World, ToR-ORd, Morotti 2021, and TP06.* ***E)*** *L-type calcium current during endocardial AP in TP06, showing spike-and-dome morphology which results from the extremely high dyadic concentration even at baseline conditions. This is to our knowledge not supported by data, and is furthermore exacerbated if a more standard formulation of driving force of I_{CaL} was used. See the section on I_{CaL} in Supplementary Methods for further comments on the driving force equation used in TP06.*

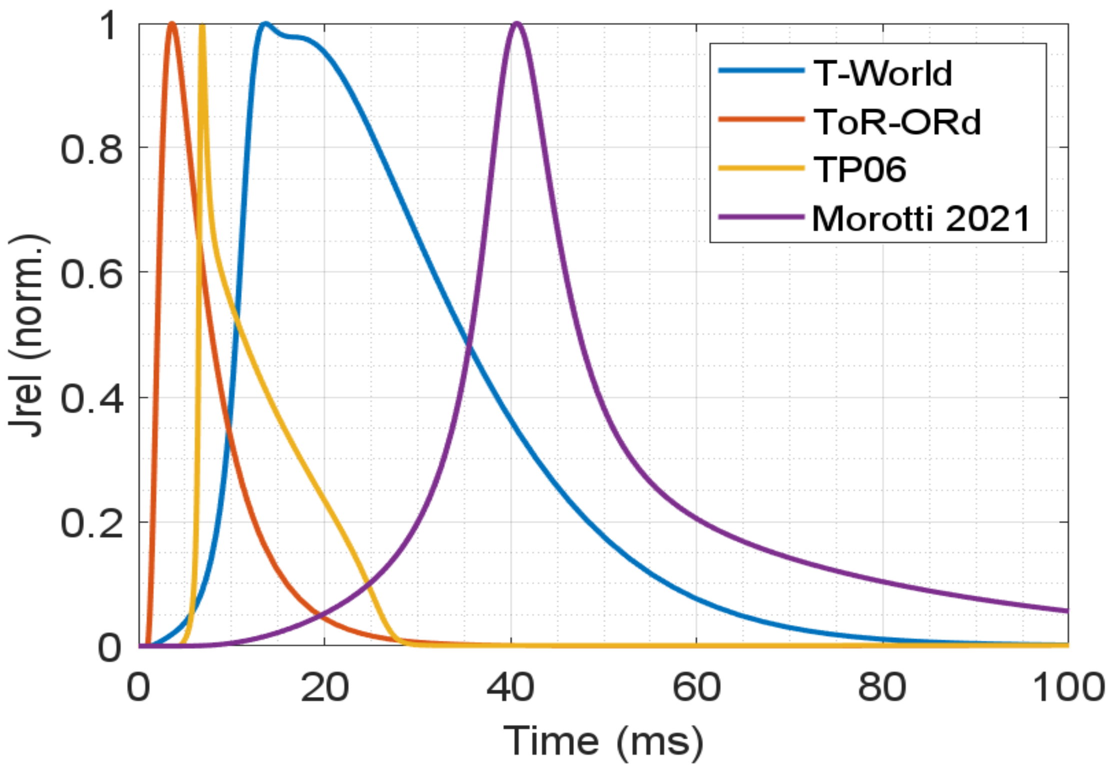

Figure S9. *Comparison of timing of normalized SR release through RyR in four models.*

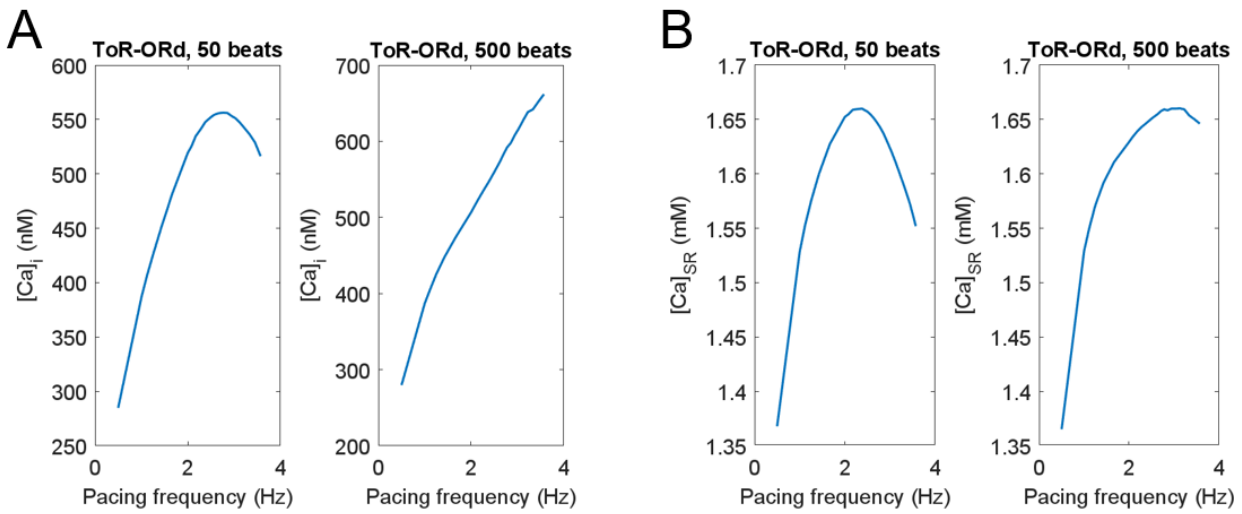

Figure S10. Effect of pre-pacing duration on biomarkers in ToR-ORd. *We note that the monotonically positive rate-dependence of peak [Ca]ᵢ ToR-ORd shown in main text Figure 2 is not at odds with the more data-like biphasic rate-dependence shown in Figure 12 of the article describing the predecessor model of O'Hara-Rudy* [18]*. In our work, we show a steady-state value after 500 beats, whereas the figure in the O'Hara-Rudy article was generated following much shorter pacing. To clearly show the importance of pre-pacing duration on the pattern of CaT peak across rates, we compared this in ToR-ORd simulated for 50 beats (**panel A**, left) and 500 beats (**panel A**, right). The model paced for 50 beats appears more data-like in its biphasicity, but this is not a behavior that would hold close to steady-state. The difference is to a large extent driven by differences in the calcium content of the sarcoplasmic reticulum (**panel B**).*

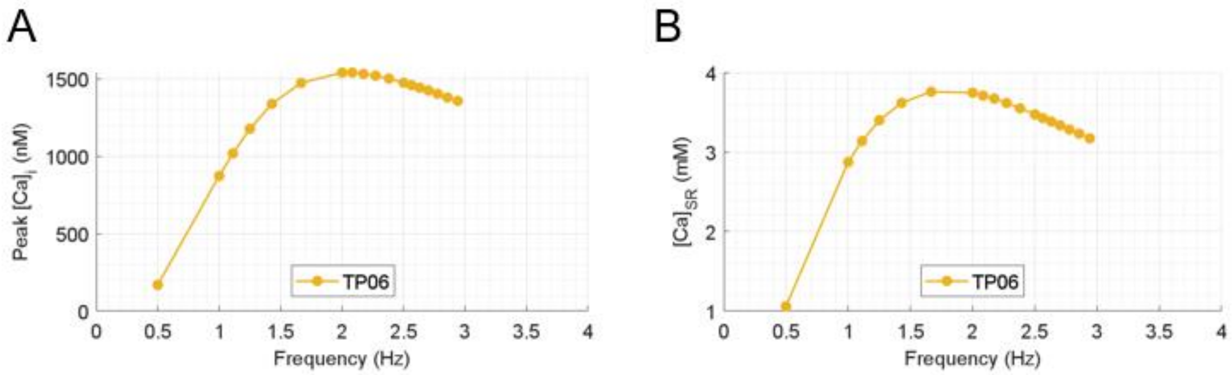

Figure S11. *Rate dependence of calcium transient peak and sarcoplasmic reticulum calcium loading in the TP06 model.*

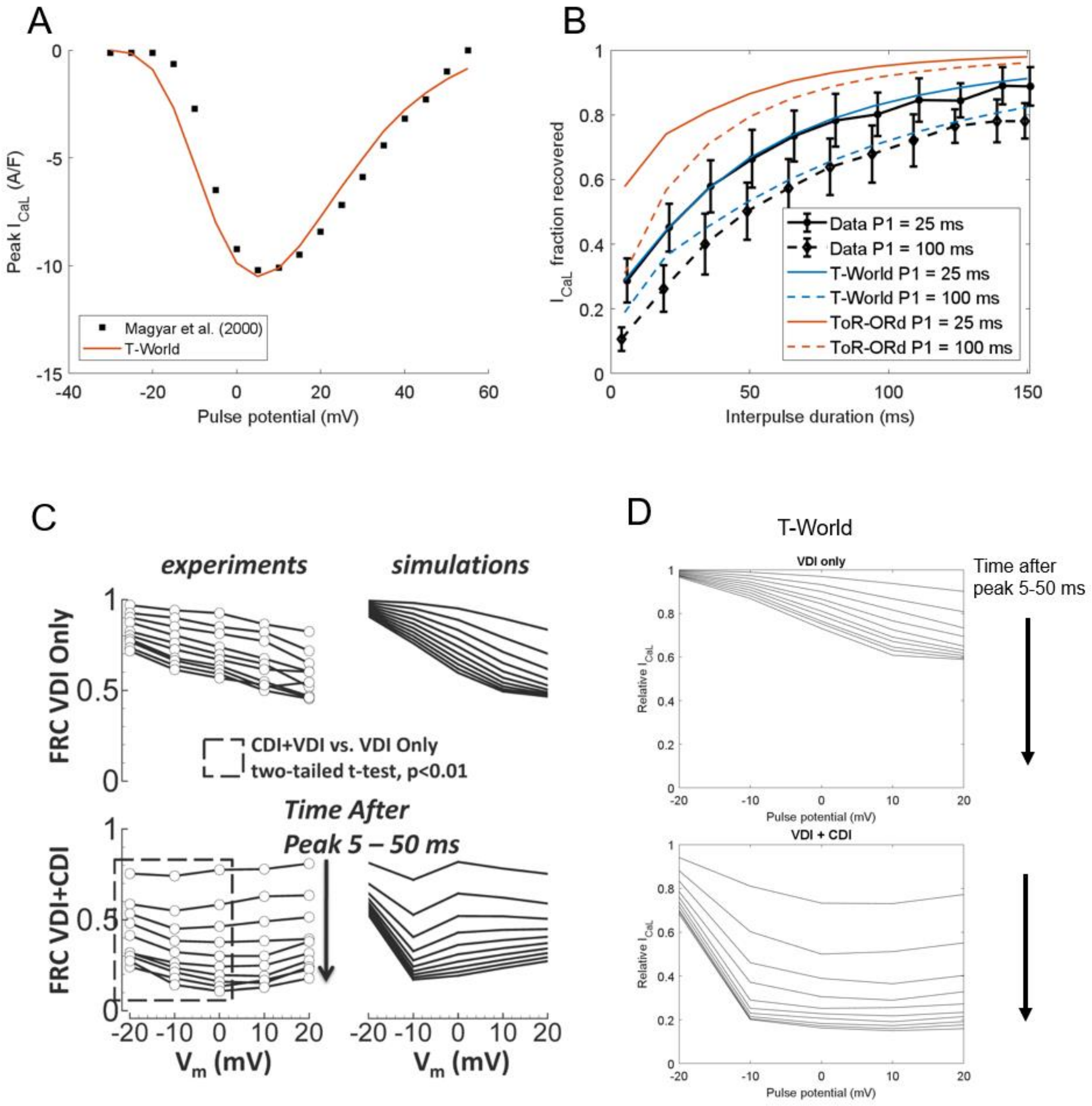

Figure S12. Properties of L-type calcium current in T-World. A) *Current-voltage (I-V) relationship, showing a similar shape and amplitude between T-World and experimental data [103].* ***B)*** *Simulated P2-P1 protocol, a two-pulse protocol used to describe the recovery from refractoriness of I_{CaL}. The duration of the first pulse (which partly inactivates I_{CaL}) is either 25 ms or 100 ms (given in the legend). It is clear from the data that the longer the first pulse, the more extensive the inactivation of I_{CaL} (dashed lines lie under corresponding solid lines). The T-World model provides a good agreement with the experimental data by Fulop et al. [104], unlike ToR-ORd, which underestimates the I_{CaL} refractoriness.* ***C)*** *Separation of voltage-dependent inactivation (VDI) and Ca-dependent inactivation (CDI) of I_{CaL}, comparing experimental data with the O'Hara-Rudy article (reproduced based on [18]). The panels show fractional current at a given voltage pulse potential 5, 10, ..., 50 ms after the peak of evoked I_{CaL}.* ***D)*** *Simulations of corresponding plots in the T-World model, which we performed as additional validation of the current formulation, given the relatively extensive changes from the preceding versions of the current in ToR-ORd (and O'Hara-Rudy). The simulations show a generally good agreement between the simulations and data, with pure VDI providing a relatively slow source of inactivation. We note that both experimental and simulation results will depend to some extent on specific conditions (such as pacing history and corresponding $[Ca]_{SR}$). In addition, it is known that Ba current used in the experimental study to obtain VDI is in fact a mix of VDI and a very small amount of CDI [160], thus slightly overestimating the VDI development speed. For those reasons, we believe it is not particularly useful to focus at whether a full quantitative agreement is achieved. Instead, it is encouraging that 1) in both O'Hara-Rudy and T-World, VDI+CDI develop much more rapidly than pure VDI, 2) VDI shows a clear voltage-dependence (faster inactivation VDI at higher potentials), whereas VDI+CDI are comparably flatter across most pulse potentials. We note that T-World was calibrated to achieve the behaviors in panels* ***A,B****, whereas panel* ***D*** *is independent validation.*

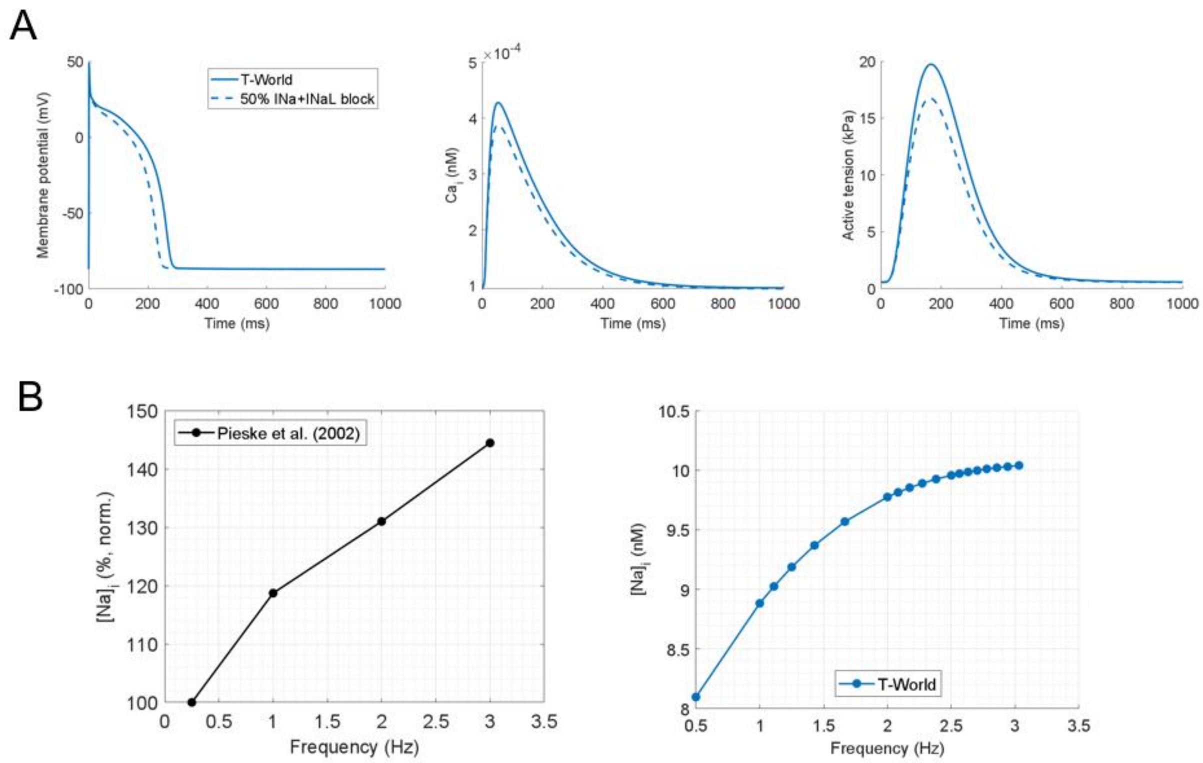

Figure S13. Validation of T-World through sodium-related protocols. A) *Response of the computational model to a combined 50% blockade of I_{Na} and I_{NaL}, similar as done in Tomek et al. [4]. It shows APD shortening and a reduction in CaT amplitude and contractility, consistent with established negatively inotropic effect of sodium blocker drugs [112–114].* ***B)*** *Experimental data [115] describing the rate-dependence of $[Na]_i$ concentration, and corresponding simulation of T-World, which also shows a positive rate-dependence relationship. In both model and the experimental study, $[Na]_i$ keeps increasing even at fast pacing where the contractility is already diminishing.*

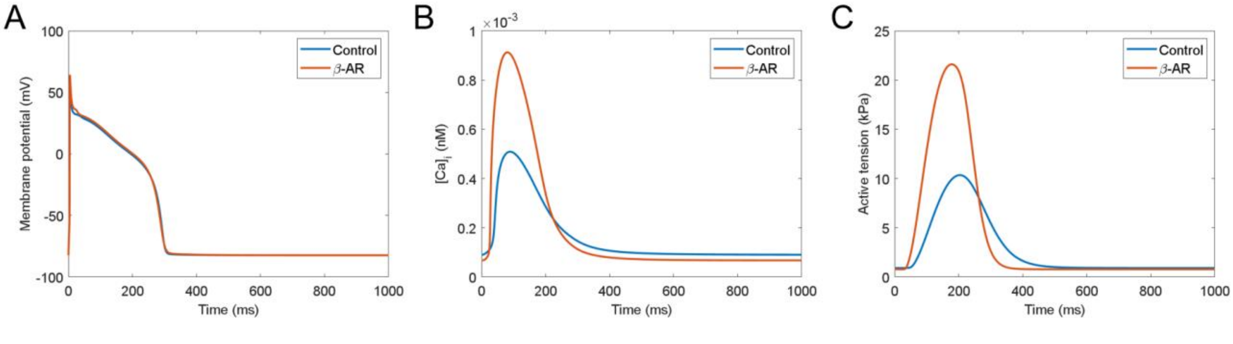

Figure S14. Effects of βAR stimulation on the Morotti2021 model. *Shown are the effects of βAR stimulation on* ***A)*** *Action potential,* ***B)*** *Ca^{2+} transient,* ***C)*** *Active tension.*

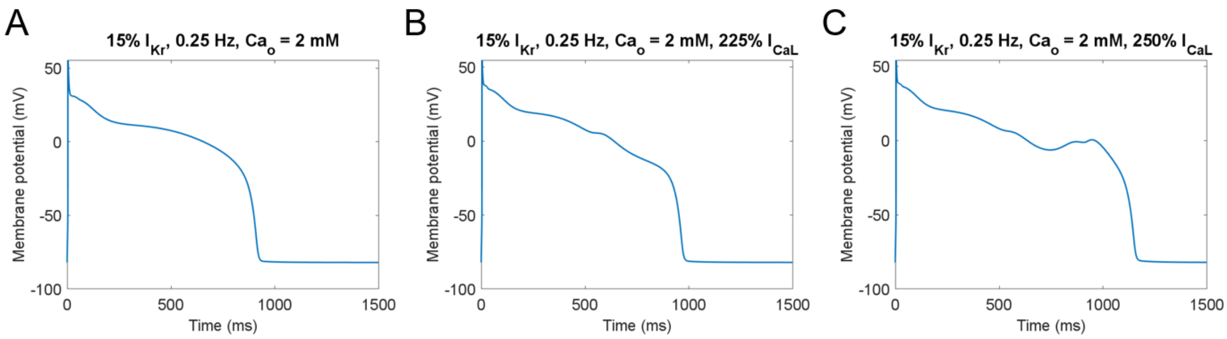

Figure S15*. *EADs in the Morotti 2021 model. ***A***) *Standard conditions used to evoke EADs as in* [54]*; 0.25 Hz pacing, 15%* I_{Kr} *availability, and extracellular calcium of 2 mM. No EADs are evoked.* ***B)*** *Demonstration that even with a substantial increase in* I_{CaL} *(225% of control, i.e., 115% increase) is still not sufficient to trigger EADs.* ***C)*** *With 250%* I_{CaL}*, an EAD is evoked. However, the unsmooth AP morphology during recovery to resting potential is not particularly similar to experimental measurements.*

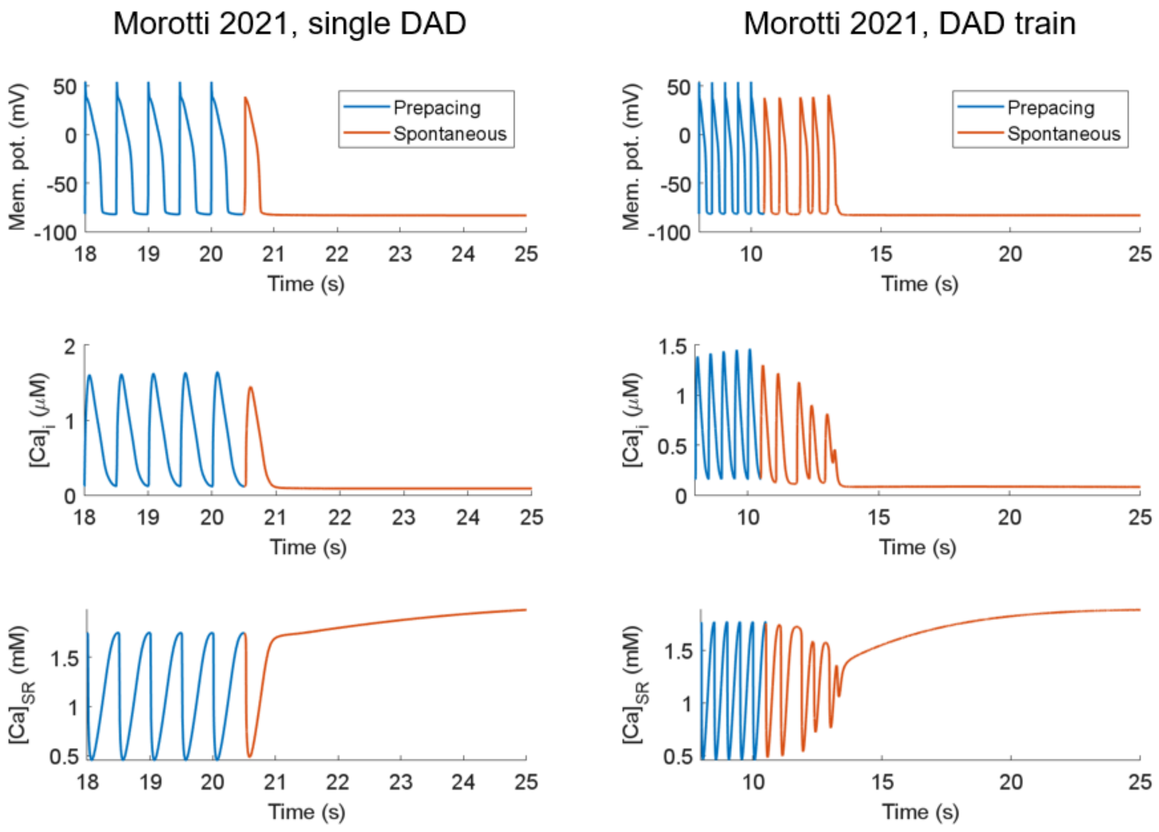

Figure S16. DADs in the Morotti2021 model*. In the* ***left*** *column are the membrane potential, intracellular calcium, and SR calcium. The cell model was pre-paced for 40 beats at 2 Hz with 0.1 µM simulated isoproterenol and extracellular calcium of 3.5 mM, following which a single beat followed by long period of quiescence was applied. Similar to Figure 4D of the main manuscript, blue traces indicate the paced beats, whereas the red ones arise from spontaneous calcium release. In the right panel is an example of a multi-DAD train, achieved using a similar protocol, which occurred at 4 mM extracellular calcium, and the pre-pacing was applied for 20 beats. In either case, endocardial model was used, with parameters myoFlag = 1 and mechFlag = 0.*

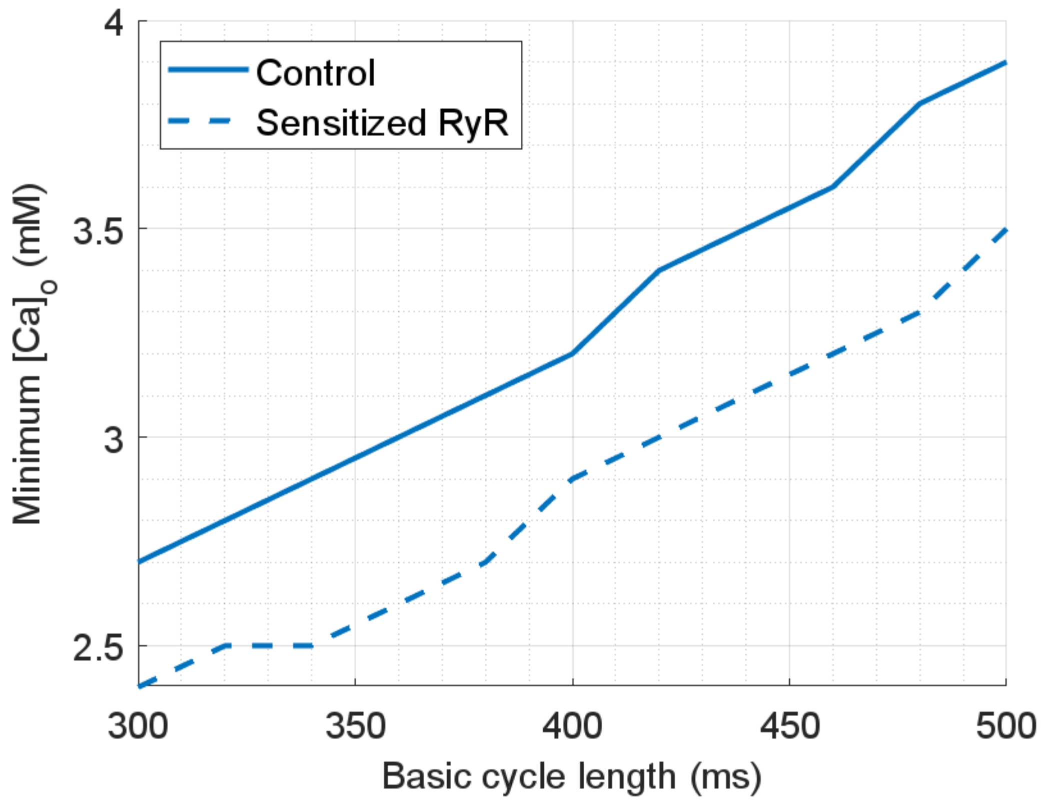

Figure S17. DAD validation. *To validate the DAD generation in T-World, we confirmed that pre-pacing at higher frequencies facilitates DADs, as observed experimentally[161]. We also simulated a version of the model with sensitized RyR (doubled RyR opening rate), which also promoted DAD formation in the presence of β-AR stimulation, consistent with experimental findings [124]. The figure shows the DAD threshold (expressed as the minimum extracellular calcium concentration needed to evoke DADs) versus pre-pacing frequency. In solid line is shown the control model, with a sensitized model (doubled transition rate from closed to open RyR state) shown by dashed line. The lower threshold for shorter basic cycle length, and for the sensitized RyR indicates increased DAD vulnerability.*

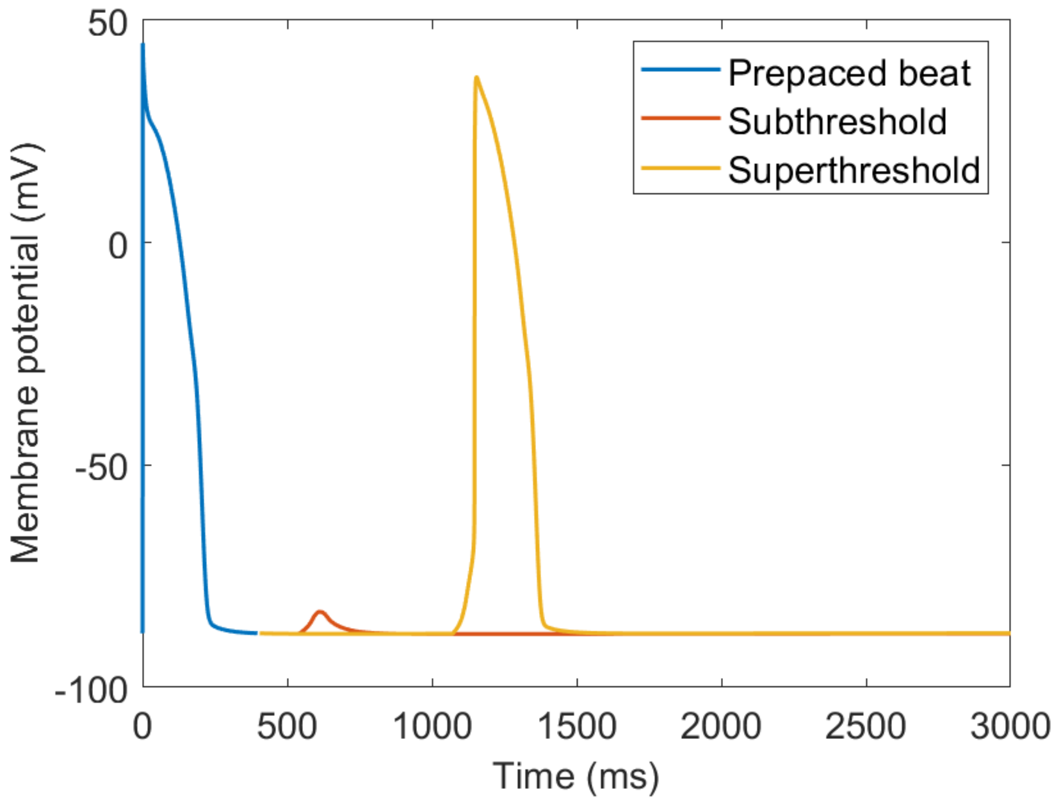

Figure S18. Examples of DADs generated by stochastic spontaneous calcium release. *See Supplementary Methods-Arrhythmia Studies for details of implementation.*

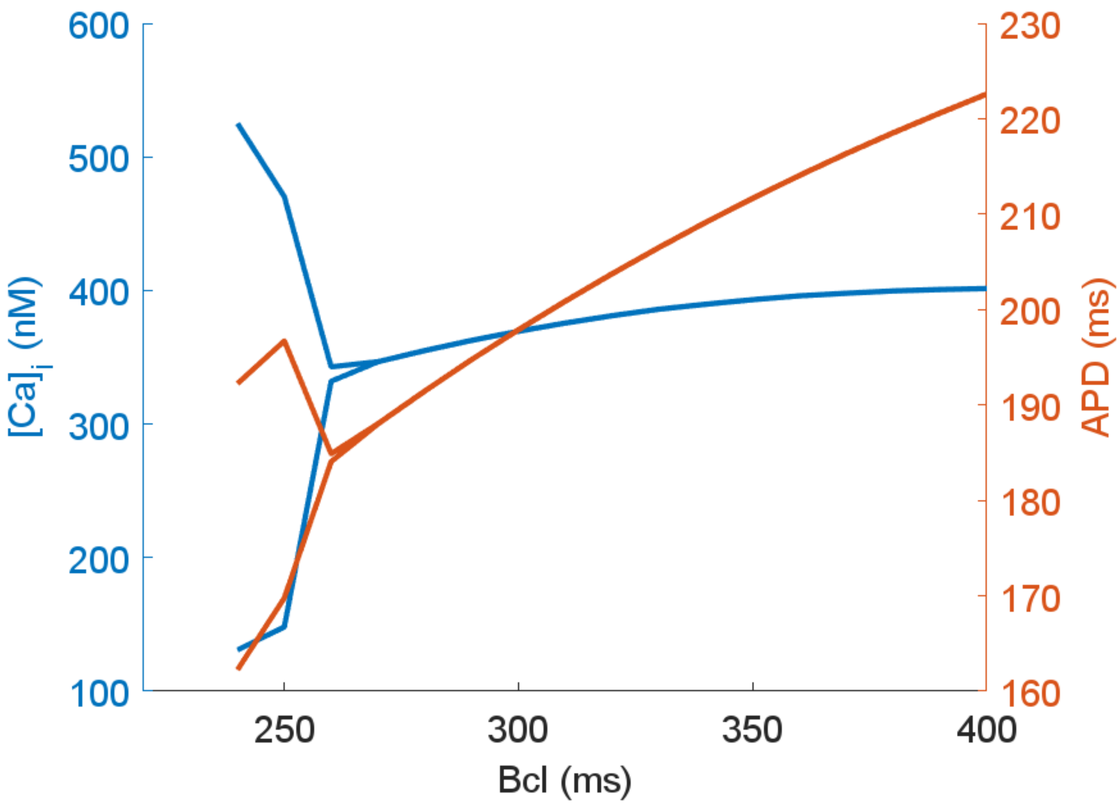

Figure S19. Rate-dependent occurrence of CaT and APD alternans in T-World.

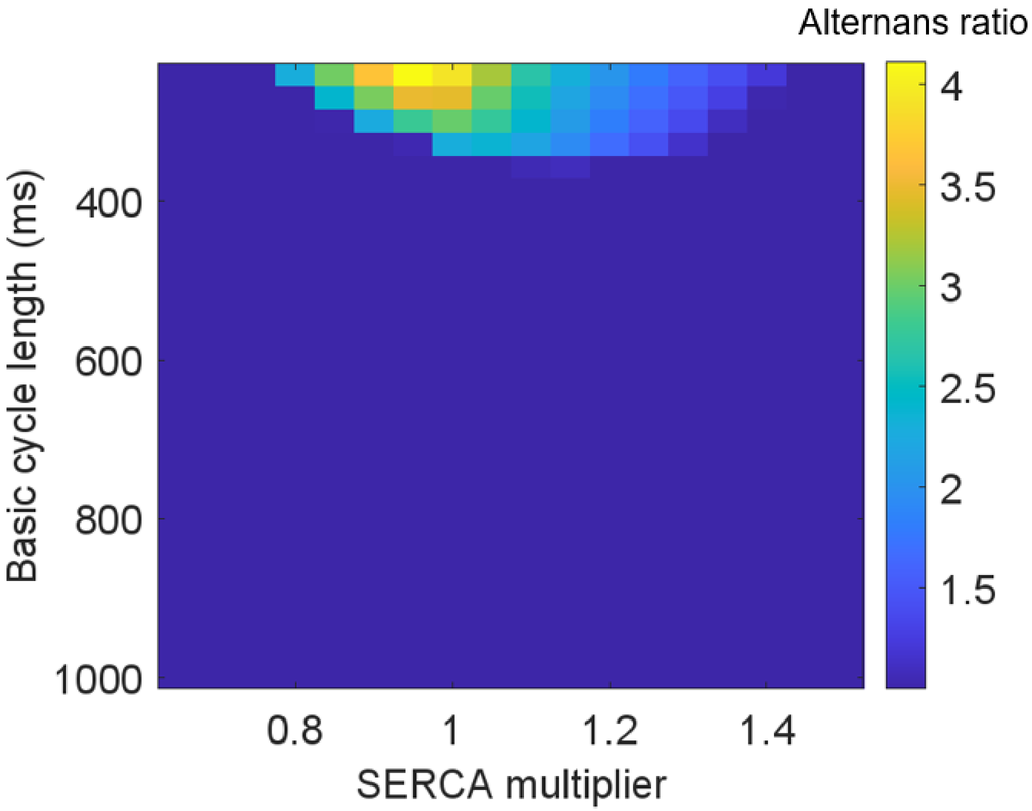

***Figure S20. Visualization of the response of alternans in ToR-ORd to changes in SERCA pumps**. The color codes calcium alternans ratio (amplitude of the larger CaT divided by the amplitude of the smaller CaT among the last two beats out of 250). On the x-axis is the multiplier of J_{up} (1 corresponds to control value), and on the y-axis is the basic cycle length used to pace the cell. It can be clearly seen that reducing SERCA availability does not lead to extension of alternans to slower frequencies; rather, it attenuates or abolishes alternans, in disagreement with experimental data. We believe this is related to a less realistic alternans mechanism in ToR-ORd, which relies on slow refilling of junctional SR from the network SR (the model uses two-compartment model of SR, unlike T-World, which uses single compartment). Available experimental data indicate the refilling to be relatively fast, faster than needed by the model to achieve alternans [162]. In addition, the qualitatively correct behavior of a large SERCA increase in ToR-ORd inhibiting alternans is also mechanistically problematic: it relies on full depletion of SR during a release, which trivially inhibits alternans, and that we described previously [163]. Such a depletion appears to be unrealistic: normal fractional release was reported to be 35%, increasing to 60% when a strong release trigger was used, but definitely not approaching full depletion [164].*

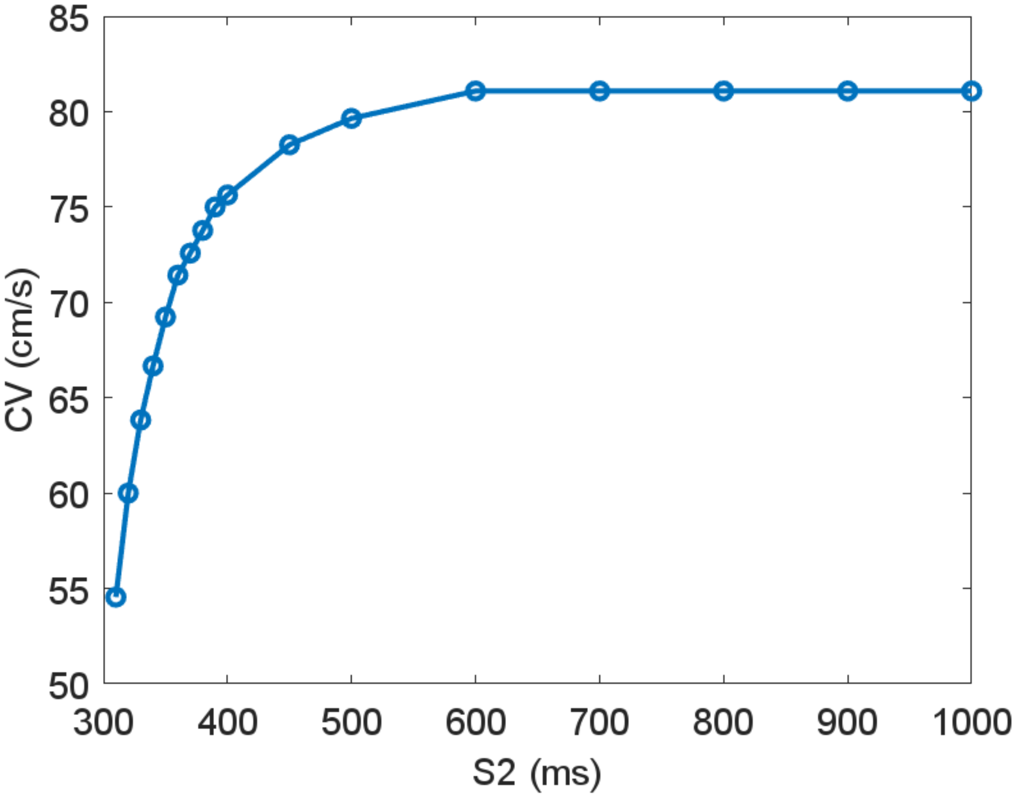

Figure S21. Conduction velocity (CV) restitution T-World. *The model manifests restitution of the conduction velocity, with conduction slowing at shorter intervals, in line with human recordings.[165,166]. Conduction velocity (CV) restitution was assessed in MonoAlg3D[158] using an S1-S2 pacing protocol in a 1-D cable of length 1cm consisting of 50 electrically coupled cell models (conductivity 0.000195 mS/m; surface to volume ratio 1400 cm^{-1}, membrane capacitance 1 μF/cm^{2}) and a spatial resolution of 0.02 cm. Following conditioning with 10 S1 beats at a cycle length of 1000 ms to ensure steady state, an S2 stimulus was applied at progressively shorter coupling intervals, from 1000ms to 200 ms. Each S2 simulation was run for an additional 500ms to ensure complete propagation of the premature wavefront and to capture any additional behavior. Membrane potential was recorded at 0.1ms temporal resolution. Activation time at each cell was defined as the first upward crossing of -60 mV during the S2 beat. CV was calculated from the final 9 mm of the cable (ignoring the first 1 mm including the stimulation site, which could confound the estimation slightly). Only S2 intervals that resulted in full propagation across the cable were included in the CV restitution analysis. Conduction velocity at S2=1000 ms (1 Hz pacing) is in line with undiseased human recordings [167].*

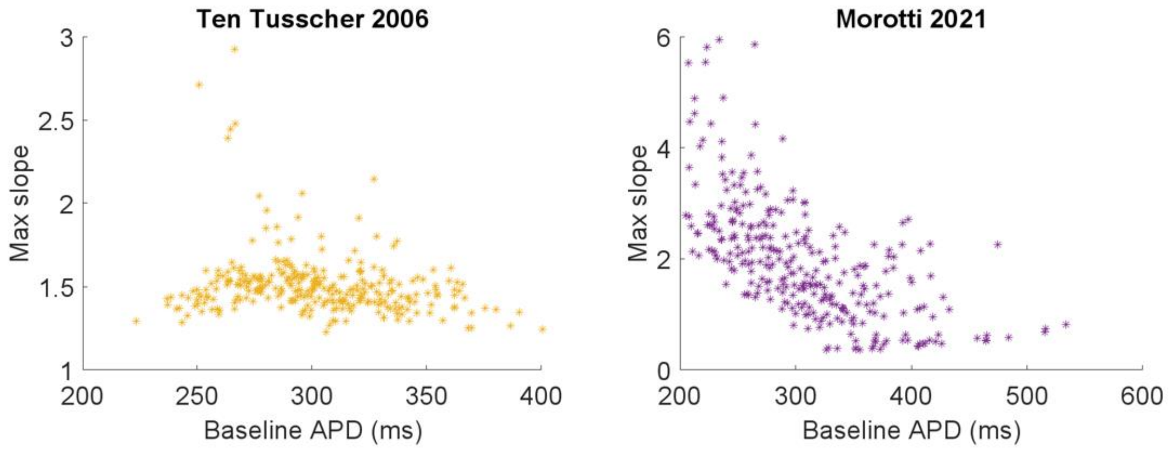

Figure S22. APD-Slope restitution in TP06 (left) and Morotti 2021 (right) models. *The simulations were carried out using the same protocol as T-World in **Figure 6D** of the main manuscript.*

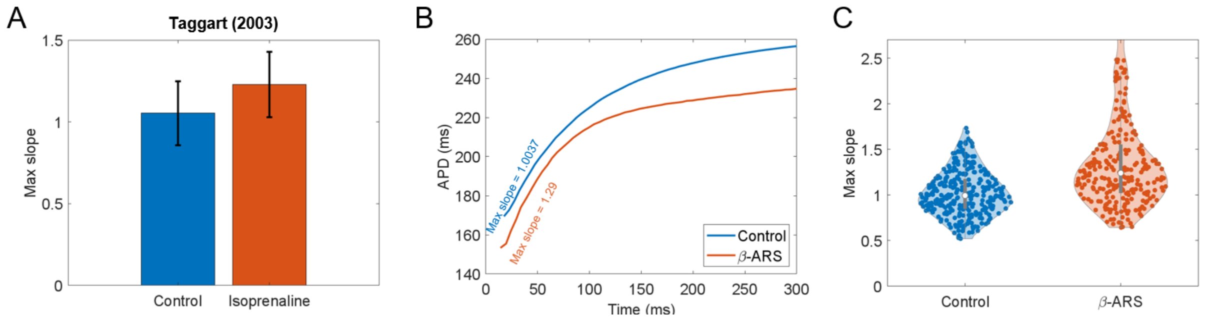

Figure S23. The effect of β-AR stimulation on restitution of T-World. A) *Human data demonstrating steepening of S1-S2 restitution with β-AR stimulation [78]. **B)** Corresponding simulation of the impact of β-AR stimulation on restitution in T-World. **C)** Simulation of a group of models with randomly perturbed parameters, showing overall restitution steepening with β-AR stimulation.*

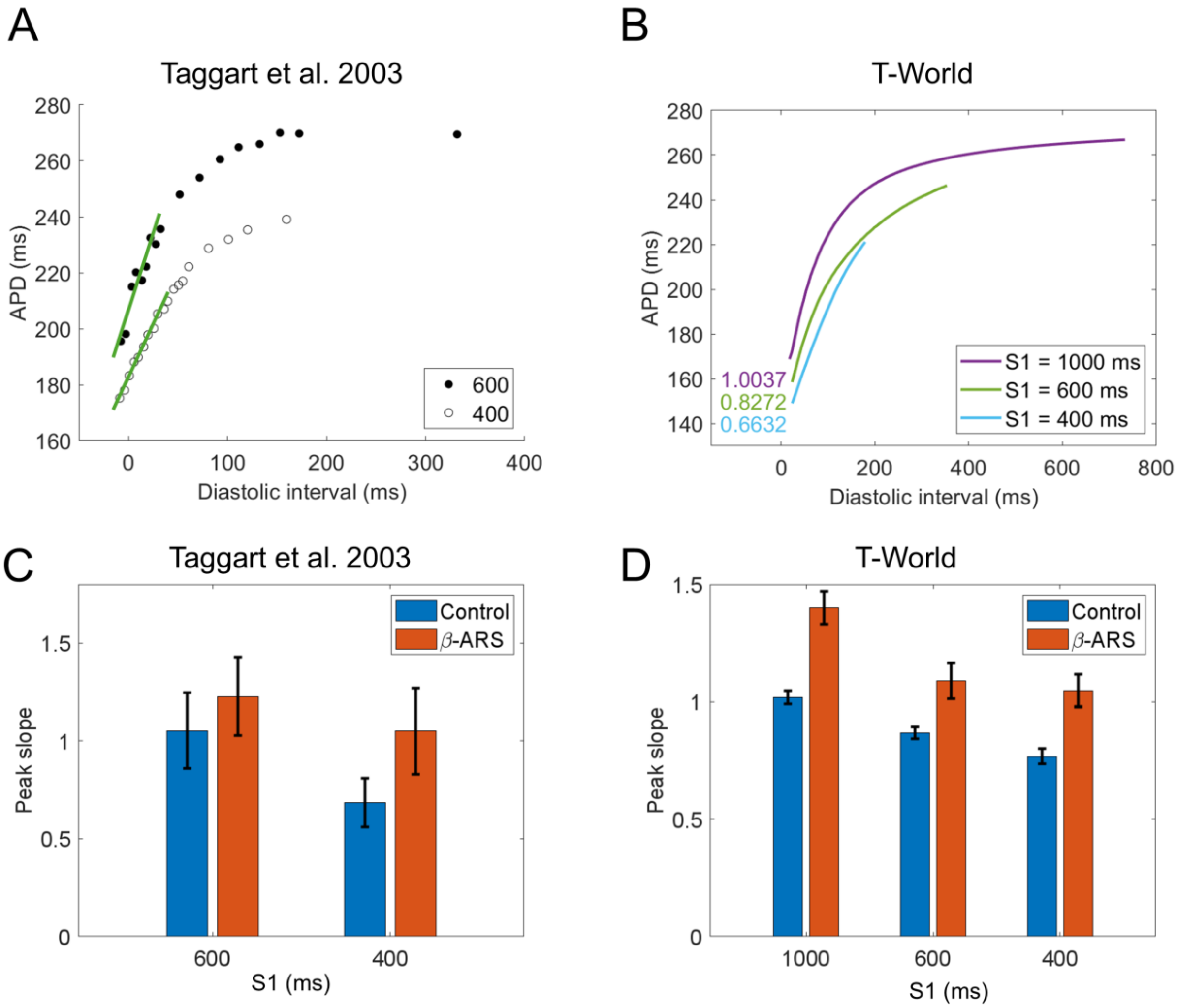

Figure S24. Modulation of S1S2 restitution slope by pre-pacing rate. A*) Examples of restitution curves with S1=600 and 400 ms from the study by Taggart et al. [78]. **B)** Corresponding simulations using T-World (peak slope of each trace is given in bottom left corner of the plot).**C)** Quantitative summary of the peak slope at two S1 basic cycle lengths, with/without β-AR stimulation, based again on Taggart et al. [78]. **D)** Corresponding simulations summarizing 300 T-World models with varied parameters, as described in Methods.*

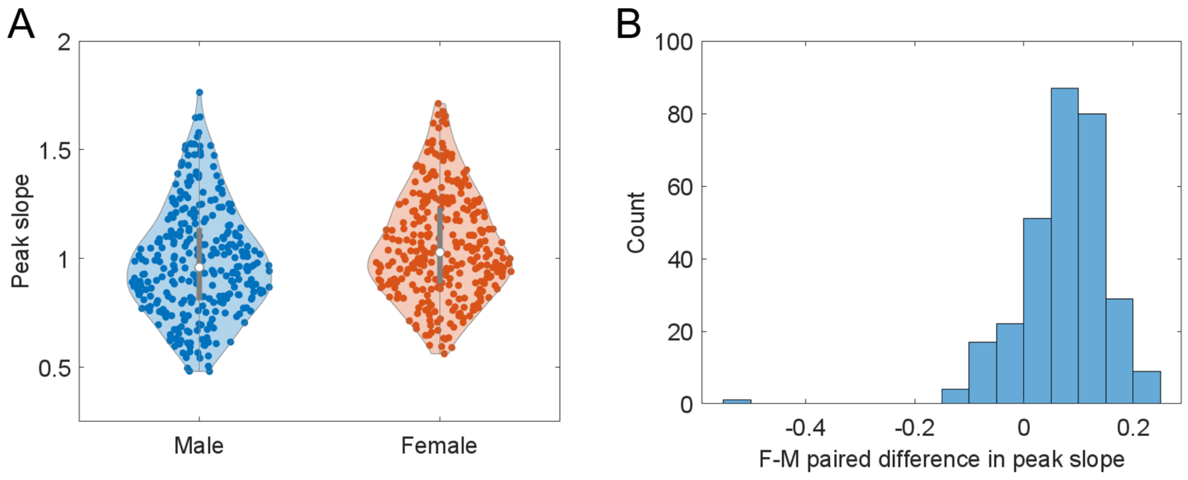

Figure S25. Sex differences in slope. A) *Comparison of peak S1S2 slope in a population of male and female models (n=300)*. *On average, the maximum slope increases by 0.092 when a myocyte is switched from male to female.* ***B)*** *Histogram of differences in peak slope between female (F) and male (M) version (85.3% models showing an increase)*

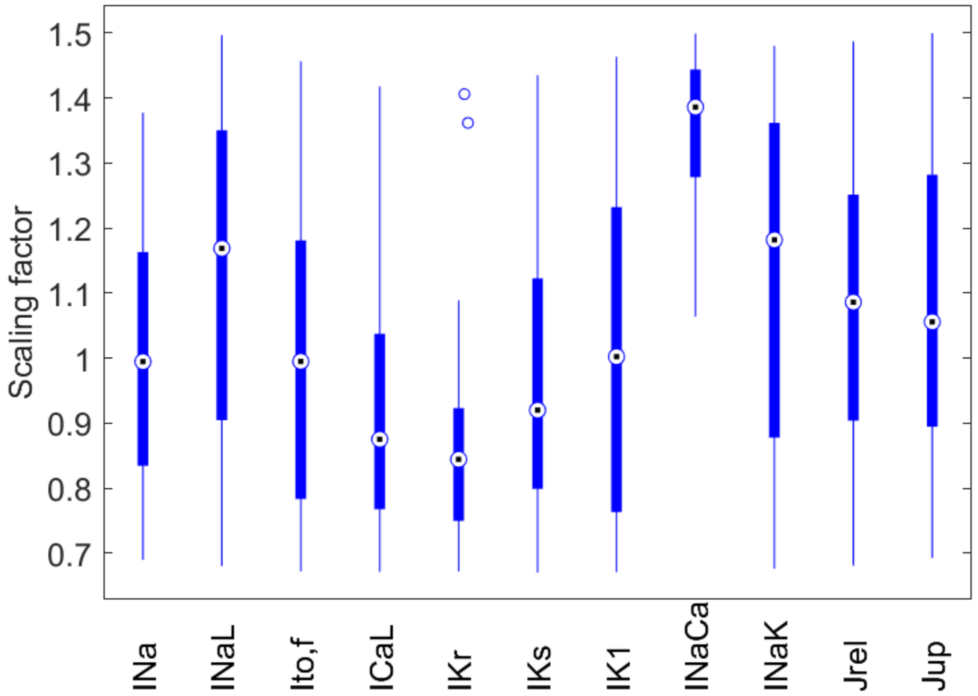

Figure S26. *Boxplots of multiplier ranges in DAD-negative models in DAD stability assessment.*

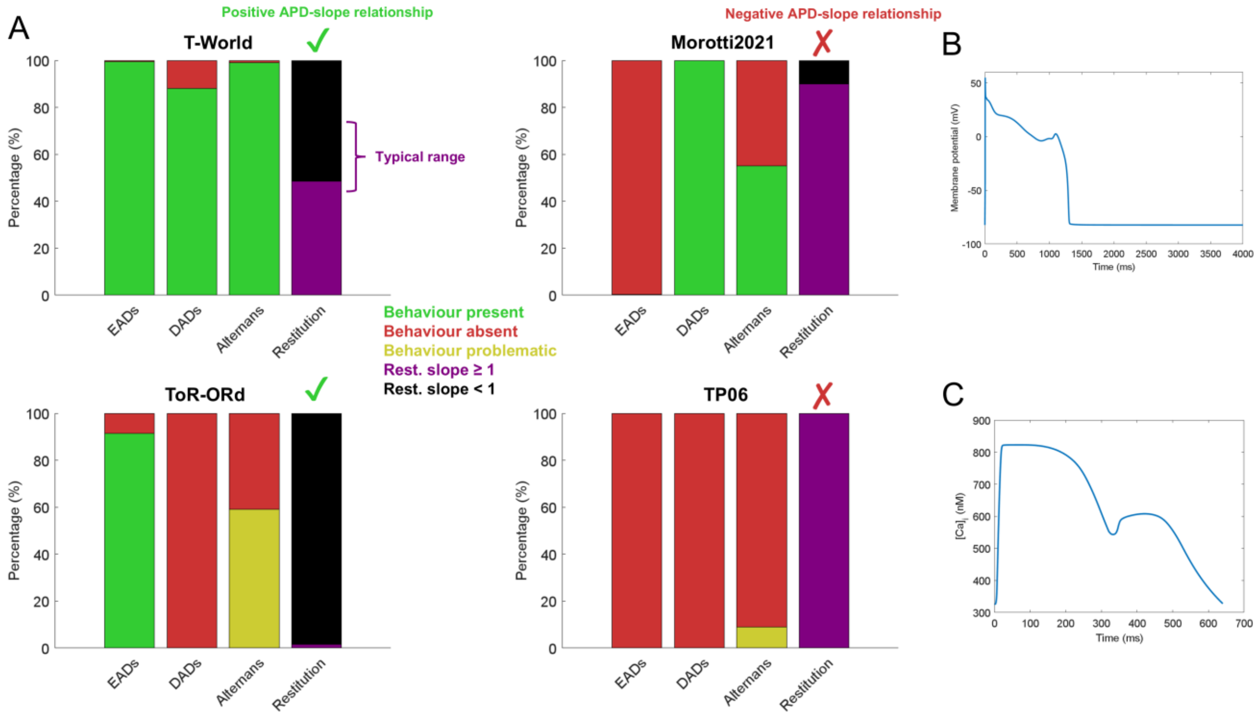

Figure S27. A) *Comparison of stability of arrhythmogenic behaviors between the models tested, and how they lead to model comparison shown in Figure 7E of the main manuscript. Typical range of fraction of models with slope > 1 is based on observations of 44%,[79] 74%,[87] and 61%.[84] of samples in human recordings.*

T-World: *All behaviors are reproduced satisfactorily as discussed in the main manuscript.*

Morotti2021*: 290 models out of 1000 passed calibration criteria in Table S6 (contractility biomarkers were ignored as the model has not been calibrated for those). Only one model has manifested an EAD-like behavior (**Panel B**), which furthermore shows a peculiar “unsmooth” repolarization morphology unlike standard experimental measurements. DADs are reproduced satisfactorily (with a minor caveat that DADs stop occurring even with increasing SR overload as shown in **Figure S16**). Alternans is present in only ca. half of the models (predominantly at very low SERCA levels), and the baseline model does not show alternans without substantial SERCA reduction, which is why label the model orange in **Figure 7E**. While the model restitution is steep, the shape of restitution curve is not particularly data-like (**Figure 6A**) and slope responds incorrectly to changes in APD (**Figure S22**), yielding orange classification.*

ToR-ORd: *615 of 1000 models passed calibration criteria. EADs are satisfactorily present in most models, but DADs are completely absent. Around half of models in the population show alternans, but this responds poorly to SERCA changes (**Figure S20**), yielding orange rating. Restitution is graded as red, since the vast majority of models are showing peak slope below 1 (we consider this a much greater limitation than the somewhat higher-than-observed proportion of models with slope > 1 in Morotti2021 and TP06 models).*

TP06: *A small fraction of models passed the calibration criteria; 313 models out of 6000. EADs and DADs were absent in the population. Only a small minority of models have shown alternans, however, those manifested a highly peculiar CaT morphology (a representative example in **Panel C**), incompatible with experimental recordings (see, e.g., the sharp peaks and near-flat plateaus). Finally, while the restitution is steep in the model, it does not respond correctly to APD perturbation (**Figure S21**), which is a major limitation, yielding orange rating.*
